# SNF2L maintains glutathione homeostasis by initiating SLC7A11 transcription through chromatin remodeling

**DOI:** 10.1038/s41419-024-07221-4

**Published:** 2024-11-12

**Authors:** Jiaguan Zhang, Zeshou Gao, Yi Yang, Zhenhao Li, Binjie Wu, Chunxin Fan, Yuyan Zheng, Ruohan Yang, Fangrong Zhang, Xiaohuang Lin, Daoshan Zheng

**Affiliations:** 1 Key Laboratory of Ministry of Education for Gastrointestinal Cancer, School of Basic Medical Sciences, Fujian Medical University, Fuzhou, China; 2 Department of Urology, The Affiliated People’s Hospital of Fujian University of Traditional Chinese Medicine, Fuzhou, China

**Keywords:** Cancer metabolism, Cell death

## Abstract

SNF2L encodes an ISWI chromatin remodeling factor that promotes gene transcription and is consistently elevated in cancers. Previous studies have shown that inhibiting SNF2L expression in cancer cells leads to significant growth suppression, DNA damage, and cell death. However, the underlying mechanisms remain poorly understood. In this study, we demonstrated that cancer cells lacking SNF2L show significantly decreased glutathione (GSH) levels, leading to elevated reactive oxygen species (ROS) and increased oxidative stress. SNF2L deficiency also heightened the sensitivity of cancer cells to APR-246, a drug that depletes GSH and induces oxidative stress, consequently decreasing cell viability and increasing ROS levels, regardless of p53 status. Mechanistically, we found that NRF2 recruits SNF2L to the SLC7A11 promoter, leading to increased chromatin accessibility and facilitating SLC7A11 transcription. This results in decreased cystine uptake and impaired GSH biosynthesis. These findings suggest that targeting the SNF2L/SLC7A11 axis could enhance the effectiveness of APR-246 by depleting GSH and increasing ROS level in cancer cells, highlighting SNF2L as a promising therapeutic target.

## Introduction

Redox homeostasis is critical for maintaining cellular function and preventing oxidative damage, as it balances the production and detoxification of reactive oxygen species (ROS) [1, 2]. Glutathione (GSH), a major intracellular antioxidant, plays a pivotal role in redox homeostasis by scavenging ROS and facilitating detoxification processes [3, 4]. The cystine/glutamate antiporter, SLC7A11, plays a vital role in maintaining the cellular redox balance and oxidative stress response [5]. Dysregulation of redox homeostasis, often associated with aberrant GSH metabolism and SLC7A11 activity, contributes to various pathologies, including cancer, where SLC7A11 overexpression promotes tumor survival by enhancing resistance to oxidative stress. Targeting the SLC7A11/GSH axis offers promising therapeutic strategies to treat malignancies and other diseases linked to oxidative stress.

Eprenetapopt, also known as APR-246, has undergone extensive clinical trials for various cancer types [6–8]. Its mechanism involves conversion to methylenequinuclidinone (MQ), an active compound that binds to and reactivates cysteine residues in mutant p53, thereby triggering cell death specifically in p53-mutant cancer cells [9, 10]. Recent studies have proposed an additional mode of action, suggesting that APR-246 may induce cell death by depleting GSH [11, 12]. Moreover, emerging evidence suggests that SLC7A11, rather than TP53 mutation status, may serve as a more reliable determinant of the APR-246 response [13]. This multifaceted approach highlights the promising potential of APR-246 as a therapeutic agent against cancer.

Redox homeostasis is significantly influenced by chromatin dynamics [14–16]. Chromatin structure and epigenetic modifications play pivotal roles in regulating metabolism and energy homeostasis through precise control of gene expression and metabolic reprogramming [17–19]. Previous studies have shown that the ARID1A subunit of SWI/SNF chromatin remodeling complexes plays a crucial role in maintaining GSH homeostasis by promoting the transcription of SLC7A11 [12]. This highlights the significance of chromatin remodeling in regulating redox homeostasis. SNF2L, the ATPase subunit of the ISWI chromatin remodeling complex, serves as a transcriptional activator and is widely expressed in human tumors [20–22]. Research indicates that SNF2L has various functions in different cancers. Its functions vary across different cancer types, acting as an oncogene in lung and cervical cancers but as a tumor suppressor in soft tissue and gastric cancers. Previous studies suggested that SNF2L enhances tumorigenesis in breast cancer cells by inhibiting DNA damage and promoting cell growth, while in gastric cancer, it is associated with maintaining cellular homeostasis [20]. This dual function highlights the context-dependent nature of SNF2L’s impact on cancer progression. Despite these findings, the precise role of SNF2L in regulating redox homeostasis is still unclear. The contribution of SNF2L to cellular redox balance and its implications for cancer biology remain to be elucidated.

In our investigation, we revealed a pivotal role for SNF2L in maintaining GSH homeostasis within cancer cells. Depletion of SNF2L leads to a reduction in intracellular cystine levels, consequently impairing GSH biosynthesis and rendering cancer cells more vulnerable to the small molecule drug eprenetapopt (APR-246). Mechanistically, we demonstrated that NRF2, a key transcription factor in stress response pathways, recruits SNF2L to the promoter region of the SLC7A11 gene, thus enhancing chromatin accessibility and initiating its transcription. These findings illuminate the regulatory function of SNF2L in modulating SLC7A11 expression and GSH homeostasis in cancer cells, highlighting its potential as a therapeutic target for cancer treatment.

## Results

### SNF2L deficiency drives the reprogramming of GSH metabolism

**Fig. 1:**
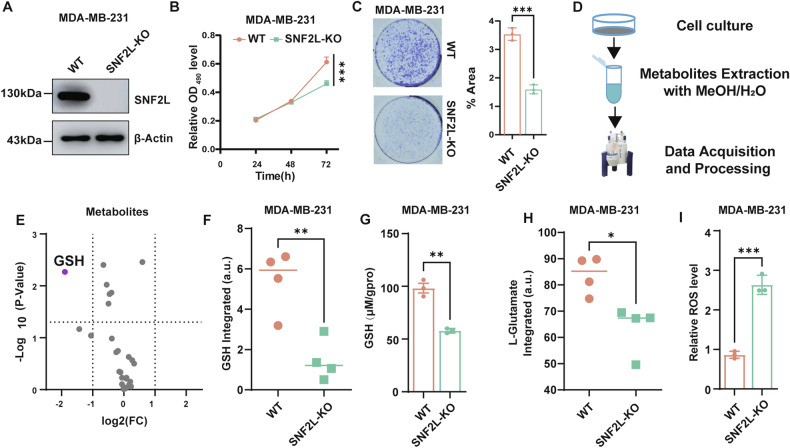
SNF2L deficiency drives the reprogramming of GSH metabolism. **A** Relative expression of SNF2L protein was analyzed using Western blotting in SNF2L-deficient and parental MDA-MB-231 cells. β-Actin was used as the loading control. **B** Cell viability was measured by the MTT assay at the indicated time points (n = 3). **C** Colony formation of SNF2L-deficient and parental MDA-MB-231 cells was measured at 14 days; the percentage of colony area was calculated using ImageJ (n = 3). **D** Flowchart showing the NMR metabolomics approach used in this work. **E** Volcano plot displaying differentially expressed metabolites between SNF2L-deficient and parental MDA-MB-231 cells (n = 4). **F** Intracellular GSH levels were measured using NMR metabolomics in SNF2L-deficient and parental MDA-MB-231 cells (n = 4). **G** Relative intracellular GSH levels were measured in SNF2L-deficient and parental MDA-MB-231 cells (n = 3). **H** Intracellular L-glutamate levels were measured using NMR metabolomics in SNF2L-deficient and parental MDA-MB-231 cells (n = 4) **I** Intracellular ROS levels were detected using DCFH-DA fluorescent probe in SNF2L-deficient and parental MDA-MB-231 cells (n = 3). The error bars are the means±SDs; All Data are representative of at least three biologically independent experiments. All *p* values were calculated using two-tailed unpaired Student’s *t tests*. *p < 0.05, **p < 0.01, ***p < 0.001.

Firstly, through analysis of the publicly available proteomic database UALCAN [23], we observed that SNF2L is elevated in multiple tumors, including breast cancer, liver cancer and lung cancers, which indicates that SNF2L may act as an oncogene in these epithelial-origin cancers (hereafter referred to as ‘cancers’) (Fig. S1A). To explore the role of SNF2L in cancer development, we employed CRISPR/Cas9 technology to generate SNF2L-deficient MDA-MB-231 breast cancer cells, as confirmed by western blotting (Fig. 1A, uncropped original western blots Fig. 1A). Subsequent assays demonstrated a notable decrease in cell growth and colony formation following SNF2L disruption (Fig. 1B, C). Similarly, in Huh-7 liver cancer cells, short hairpin RNA (shRNA)-mediated knockdown of SNF2L dramatically suppressed cell proliferation and colony formation (Fig. S1B–D), further highlighting its potential as an oncogenic factor in various cancer types.

Metabolic reprogramming, which is a hallmark of cancer cells, is closely linked to the chromatin state. To identify metabolic changes associated with SNF2L deficiency, we performed NMR-based metabolomics analysis comparing SNF2L-deficient and parental MDA-MB-231 cells (Fig. 1D, Fig. S1E, F). We employed reduced spectra to identify differential metabolites by searching a database of about 500 metabolites, primarily sourced from The Human Metabolome Database (HMDB). A total of 29 metabolites were successfully identified and annotated. We found only GSH, a key regulator of redox homeostasis, exhibited significant differential expression (fold change > 2, p < 0.05) (Fig. 1E, F, Supplementary Table S1). Subsequently, we confirmed a significant reduction in intracellular GSH levels upon SNF2L deficiency (Fig. 1G). Additionally, our metabolomics analysis revealed a decrease in the level of L-glutamate, a precursor of GSH biosynthesis, in SNF2L-deficient cells compared to parental cells (Fig. 1H). Given the pivotal role of GSH in antioxidant defense, we investigated whether SNF2L deficiency impacted intracellular ROS levels. Consistent with diminished GSH levels, SNF2L-deficient cells exhibited elevated ROS levels detected using DCFH-DA fluorescent probe (Fig. 1I). This finding indicates heightened oxidative stress in these cells.

In summary, our results underscore the importance of the critical role of SNF2L in maintaining GSH homeostasis, which influences metabolic pathways and redox balance in cancer cells. These findings illuminate potential therapeutic avenues targeting SNF2L in cancer treatment.

### Cancer cells lacking SNF2L are susceptible to Eprenetapopt (APR-246)

APR-246 (PRIMA-1^met^) was identified as a targeted drug capable of restoring mutant p53 to its wild-type conformation and restoring transcriptional activity. Recent studies have suggested that APR-246 also binds to GSH, leading to its depletion and subsequent tumor cell death. Given the proposed role of SNF2L in maintaining GSH homeostasis, we sought to investigate whether SNF2L contributes to APR-246-induced cell death.

**Fig. 2:**
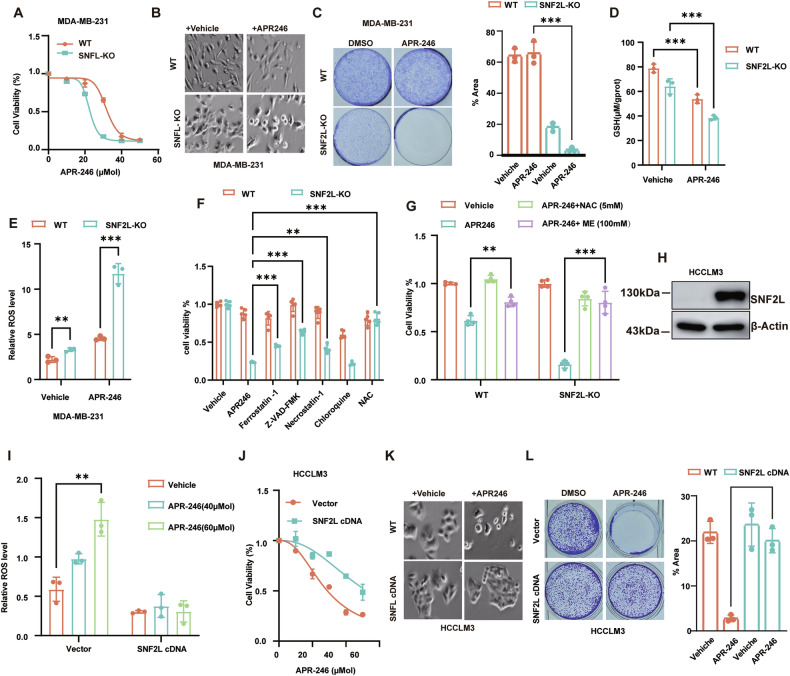
Cancer cells lacking SNF2L are susceptible to eprenetapoot (APR-246). **A** Cell viability of SNF2L-deficient and parental MDA-MB-231 cells was assessed after treatment with APR-246 at the indicated concentrations for 48 hours. **B** Cell morphology of SNF2L-deficient and parental MDA-MB-231 cells was examined after treatment with APR-246 at 20 µM for 18 hours. **C** Colony formation assays were performed for SNF2L-deficient and parental MDA-MB-231 cells after treatment with 10 µM APR-246 for 10 days; the percentage of colony area was calculated using ImageJ (n = 3). **D** Relative GSH levels were measured in SNF2L-deficient and parental MDA-MB-231 cells after treatment with 20 μM APR-246 for 24 hours (n = 3). **E** Relative ROS levels were detected using DCFH-DA fluorescent probe in SNF2L-deficient and parental MDA-MB-231 cells after treatment with 20 μM APR-246 for 24 hours (n = 4). **F** Cell viability of SNF2L-deficient and parental MDA-MB-231 cells was measured by MTT assay after treatment with 20 μM APR-246 for 48 hours and co-treatment with or without 20 µM ferrostatin-1, 20 µM Z-VAD-FMK, 20 µM necrostatin-1, 50 µM chloroquine or 5 mM NAC (n = 5). **G** Cell viability of SNF2L-deficient and parental MDA-MB-231 cells was measured by MTT assay after treatment with 20 μM APR-246 for 48 hours and treatment with or without 5 mM NAC or 100 µM β-ME (n = 3). **H** Relative expression of SNF2L protein was analyzed using Western blotting in the indicated stable cells. β-Actin was used as the loading control. **I** Intracellular ROS levels were detected using DCFH-DA fluorescent probe in parental HCCLM3 cells and HCCLM3 cells stably expressing SNF2L after treatment with 40 µM or 60 µM APR-246 for 24 hours (n = 3). **J** Cell viability was measured using the MTT assay in parental HCCLM3 cells and HCCLM3 cells stably expressing SNF2L after treatment with APR-246 for 48 hours (n = 3). **K** Cell morphology of parental HCCLM3 cells and HCCLM3 cells stably expressing SNF2L was examined after treatment with 40 µM APR-246 for 18 hours. **L** Colony formation assays were performed for parental HCCLM3 cells and HCCLM3 cells stably expressing SNF2L after treatment with 10 µM APR-246 for 10 days; the percentage of colony area was calculated using ImageJ (n = 3). Error bars are the mean±SD. All data are representative of at least three biologically independent experiments. *p* values were calculated using two-tailed unpaired Student’s *t tests*. *p < 0.05, **p < 0.01, ***p < 0.001.

Our results demonstrated that the absence of SNF2L expression significantly reduced the viability of SNF2L-deficient MDA-MB-231 cells treatment with APR-246 (Fig. 2A, B). Additionally, APR-246 treatment markedly inhibited colony formation in SNF2L- deficient cells (Fig. 2C). Furthermore, APR-246 treatment resulted in a further decrease in intracellular GSH levels (Fig. 2D), accompanied by an increase in ROS levels in SNF2L-deficient cells compared to those in parental cells treatment with APR-246 (Fig. 2E).

Next, we aimed to identify the specific cell death pathways activated by APR-246. To do this, we treated the cells with various cell death inhibitors in combination with APR-246. Our results showed that cell death was partially inhibited by the ferroptosis inhibitor Ferrostatin-1, the apoptosis inhibitor Z-VAD-FMK and the necrosis inhibitor Necrostatin-1. This suggests that APR-246 can induce multiple programmed cell death pathways (Fig. 2F), consistent with findings from previous studies [9].

To further verify that SNF2L deficiency leads to cell death through oxidative stress, we treated the cells with ROS scavengers N-acetylcysteine (NAC) and 2-mercaptoethanol (2-ME) together with APR-246. Importantly, treatment with NAC and 2-ME significantly prevented the cell death induced by APR-246 (Fig. 2G). This protective effect, observed with NAC and 2-ME, further supports the concept that SNF2L deficiency enhances oxidative stress in SNF2L-deficient cancer cells. Extending these results, we established SNF2L-overexpressing HCCLM3 liver cancer cells (a SNF2L-deficient cell line) with stable SNF2L cDNA expression (Fig. 2H, uncropped original western blots Fig. 2H). Conversely, ectopic expression of SNF2L protected cancer cells from APR-246-induced ROS elevation (Fig. 2I) and cell death (Fig. 2J, K). This resistance to APR-246 in SNF2L-overexpressing cells was further confirmed by measuring cell survival via colony formation assays (Fig. 2L).

In summary, our results suggest that SNF2L plays a crucial role in modulating cellular responses to APR-246-induced oxidative stress and cell death, potentially through its involvement in maintaining GSH homeostasis.

### SNF2L regulates cancer cell sensitivity to APR-246 independently of mutant p53

**Fig. 3:**
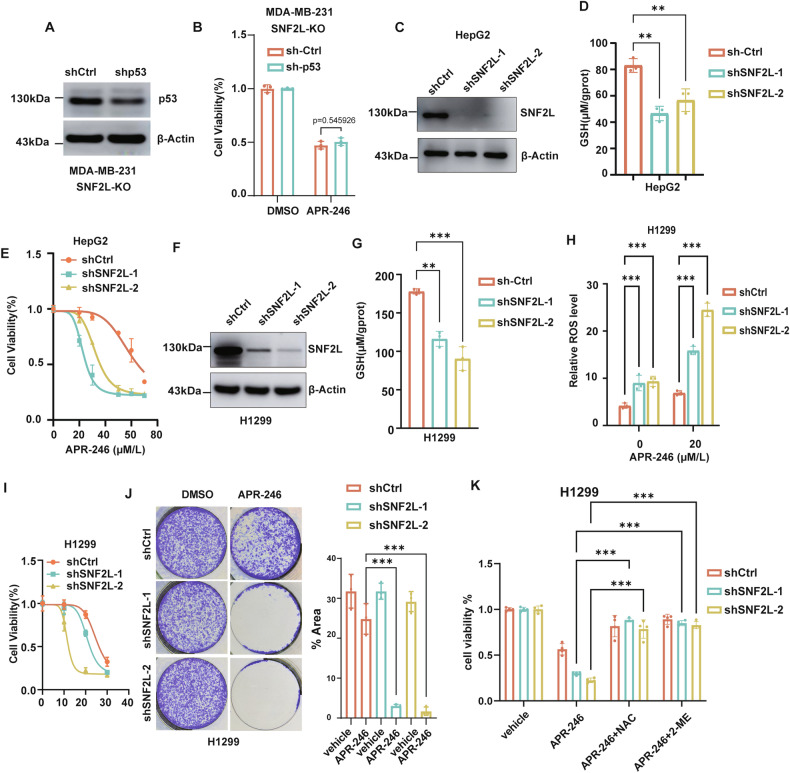
SNF2L regulates cancer cell sensitivity to APR-246 independently of mutant p53. **A** Relative expression of p53 protein was analyzed by western blotting in SNF2L-deficient MDA-MB-231 cells, and β-actin was used as the loading control. **B** Cell viability was measured using the MTT assay in SNF2L-deficient MDA-MB-231 cells with p53 knockdown after treatment with 20 µM APR-246 for 48 hours (n = 3). **C** Relative expression of p53 protein was detected by Western blotting in HepG2 cells, and β-actin was used as the loading control. **D** Relative GSH levels were measured in SNF2L-knockdown and parental HepG2 cells (n = 3). **E** Cell viability of SNF2L-knockdown and parental HepG2 cells was assessed after treatment with APR-246 at the indicated concentrations for 48 hours (n = 4). **F** Relative expression of SNF2L protein was analyzed by Western blotting in SNF2L knockdown H1299 cells, and β-actin was used as the loading control. **G** Relative GSH levels were measured in SNF2L knockdown and parental H1299 cells (n = 3). **H** Relative ROS levels were detected using DCFH-DA fluorescent probe in SNF2L knockdown and parental H1299 cells after treatment with 20 µM APR-246 for 12 hours (n = 4). **I** Cell viability of SNF2L knockdown and parental H1299 cells was measured by MTT assay after treatment with APR-246 for 48 hours. (n = 5) **J** Colony formation assays were performed on SNF2L knockdown and parental H1299 cells following treatment with 10 µM APR-246 for 10 days; the percentage of colony area was calculated using ImageJ (n = 3). **K** Cell viability of SNF2L knockdown and parental H1299 cells was measured by MTT assay after treatment with 20 µM APR-246 for 24 hours, with or without cotreatment with 5 mM NAC or 100 µM β-ME (n = 4). Error bars are the mean±SD. All data are representative of at least three biologically independent experiments. *p* values were calculated using two-tailed unpaired Student’s *t tests* (**B**, **H**), two-way ANOVA with Tukey’s multiple comparisons test (**K**) or one-way ANOVA with Tukey’s multiple comparisons test (**D**, **G**). *p < 0.05, **p < 0.01, ***p < 0.001.

Since APR-246 has been proposed to potentially restore the tumor-suppressive activity of mutant p53, we sought to investigate whether mutant p53 is necessary for SNF2L to modulate APR-246 sensitivity. We treated SNF2L-deficient cells with APR-246 after knocking down mutant p53 (Fig. 3A, uncropped original western blots Fig. 3A). Surprisingly, the deletion of mutant p53 did not significantly affect the viability of SNF2L-deficient cells treated with APR-246 (Fig. 3B). These results suggest that SNF2L modulates APR-246 sensitivity independently of mutant p53. Additionally, we eliminated the expression of SNF2L in HepG2 cells, which harbor a wild-type p53 gene (Fig. 3C, uncropped original western blots Fig. 3C). Consistently, we discovered that the GSH level was decreased in HepG2 cells with SNF2L deletion (Fig. 3D). Additionally, the viability of HepG2 cells treated with APR-246 was considerably reduced by the deletion of SNF2L (Fig. 3E). These results indicate that SNF2L might influence cellular redox status and modulate sensitivity to APR-246 through a mutant p53-independent mechanism.

To further test this hypothesis, we knocked down SNF2L expression using shRNA in p53-null H1299 lung cancer cell lines (Fig. 3F, uncropped original western blots Fig. 3F). Consistently, knockdown of SNF2L in H1299 cells led to a significant decrease in intracellular GSH levels (Fig. 3G), resulting in a synergistic increase in intracellular ROS levels (Fig. 3H) and heightened susceptibility to APR-246-induced stress was identified through cell survival assays (Fig. 3I, J). Additionally, both NAC and 2-ME dramatically prevented the cell death induced by APR-246 (Fig. 3K). In summary, these findings further support the notion that SNF2L enhances APR-246 sensitivity by exacerbating oxidative stress in cancer cells, regardless of their mutant p53 status.

### SNF2L maintains GSH homeostasis by regulating SLC7A11 expression

**Fig. 4:**
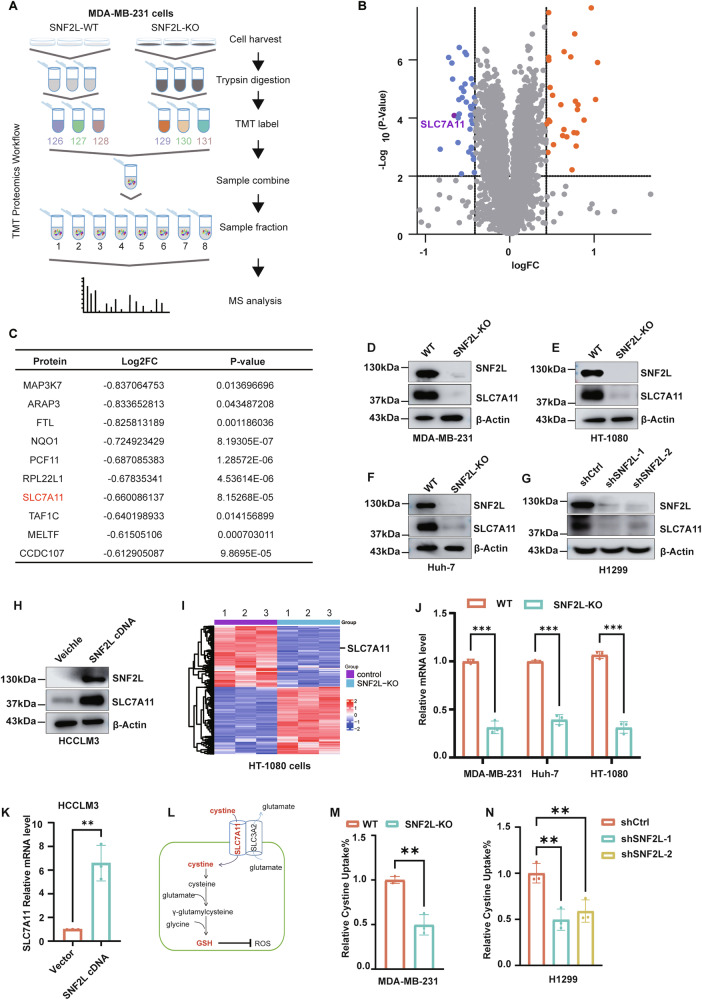
SNF2L maintains GSH homeostasis by regulating SLC7A11 expression. **A** Flowchart illustrating the TMT-based quantitative proteomics approach by mass spectrometry used in this study. **B** Volcano plot displaying differentially expressed proteins between SNF2L-deficient and parental MDA-MB-231 cells identified by mass spectrometry (n = 3). **C** List of the top 10 most strongly differentially expressed proteins between SNF2L-deficient and parental MDA-MB-231 cells identified by mass spectrometry. **D–G** Relative expression of SNF2L and SLC7A11 proteins was analyzed by Western blotting in the MDA-MB-231 (**D**), HT-1080 (**E**), Huh-7 (**F**) and H1299 (**G**) cell lines, with β-actin used as the loading control (n = 3). **H** Relative expression of SNF2L and SLC7A11 was analyzed by Western blotting in HCCLM3 cells, and β-actin was used as the loading control (n = 3). **I** Heatmap of RNA-seq expression data showing differentially regulated SLC7A11 genes in SNF2L-deficient and parental HT-1080 cells (n = 3). **J** The relative mRNA levels of SLC7A11 were measured by RT-qPCR in the indicated cells (n = 3). **K** The relative mRNA levels of SLC7A11 were measured by RT-qPCR in parental HCCLM3 cells and HCCLM3 cells stably expressing SNF2L (n = 3). **L** Diagram showing the regulatory network of GSH metabolism by the SLC7A11 gene. **M** Relative cystine uptake capacity was measured in SNF2L-deficient and parental MDA-MB-231 cells. **N** Relative cystine uptake capacity was measured in SNF2L knockdown and parental H1299 cells. Error bars are the mean±SD, all data are representative of at least three biologically independent experiments. *p* values were calculated using two-tailed unpaired Student’s *t tests* (**J**, **K**, **M**) *or* one-way ANOVA with Tukey’s multiple comparisons test (**N**). *p < 0.05, **p < 0.01, ***p < 0.001.

To identify SNF2L target proteins involved in GSH homeostasis in cancer cells, we conducted a mass spectrometry-based quantitative proteomic screening of SNF2L-deficient and parental MDA-MB-231 cells (Fig. 4A). Among the 5477 quantified proteins, we identified 22 proteins with significantly elevated expression and 14 proteins with reduced expression upon loss of SNF2L (fold change>1.5, p < 0.05) (Supplementary Table S2). Notably, solute carrier family 7 member 11 (SLC7A11), a critical regulator of GSH homeostasis in tumor cells, was substantially downregulated in the absence of the SNF2L protein (Fig. 4B, C). Western blotting confirmed the dramatic reduction of SLC7A11 in SNF2L-deficient MDA-MB-231 cells (Fig. 4D, uncropped original western blots Fig. 4D), indicating the role of SNF2L in regulating SLC7A11 levels in cancer cells. To determine whether the regulation of SLC7A11 by SNF2L is consistent across different cancer cells, we disrupted SNF2L expression in HT-1080 fibrosarcoma cells, Huh-7 liver cancer cells and H1299 lung cancer cells. Indeed, SLC7A11 protein levels were significantly decreased in Huh-7, HT-1080 and H1299 cells upon SNF2L deletion (Fig. 4E–G, uncropped original western blots Fig. 4E–G). Conversely, SLC7A11 protein levels were elevated in HCCLM3 cells overexpressing SNF2L (Fig. 4H, uncropped original western blots Fig. 4H). To further confirm SNF2L’s regulation of SLC7A11 expression, H1299 cells were infected with serial dilutions of lentiviral particles encoding SNF2L. We observed a corresponding increase in SLC7A11 expression (Fig. S2A).

Given the role of SNF2L as a transcription activator, we validated the transcriptional alteration of SLC7A11 in HT-1080 fibrosarcoma cells using transcriptome sequencing. The transcription level of the SLC7A11 gene was reduced by SNF2L deficiency, consistent with the proteomic results (Fig. 4I, Supplementary Table S3). Additionally, we confirmed the downregulation of SLC7A11 mRNA in viable cancer cells with SNF2L deletion (Fig. 4J), whereas SLC7A11 mRNA was elevated in HCCLM3 cells overexpressing SNF2L (Fig. 4K). Together, our results suggest that SNF2L modulates SLC7A11 expression across multiple cancer cell types through gene transcription.

SLC7A11 is crucial for the uptake of cystine, a substrate for GSH biosynthesis (Fig. 4L). Therefore, we investigated whether SLC7A11 downregulation led to compromised cystine uptake in SNF2L-deficient cells using a cystine uptake assay. As expected, SNF2L-deficient cells exhibited significantly reduced cystine absorption, accompanied by a notable decrease in GSH biosynthesis (Fig. 4M, N). Furthermore, we confirmed that cystine uptake capacity increased in HCCLM3 cells stably expressing SNF2L (Fig. S2B). These findings suggest that SNF2L depletion diminishes cystine uptake, thereby impairing GSH biosynthesis, highlighting the regulatory role of SNF2L in maintaining GSH homeostasis in cancer cells.

Next, we validated the SNF2L/SLC7A11/GSH regulatory axis in patient-derived primary breast cancer cells. Western blotting confirmed a dramatic reduction in SLC7A11 in primary breast cancer cells with SNF2L knocked down by shRNA (Fig. S3A). Furthermore, our results indicated decreased levels of GSH in these cells (Fig. S3B). Additionally, we confirmed that SNF2L disruption enhances the sensitivity of patient-derived primary breast cancer cells to APR-246 (Fig. S3C). These findings highlight the potential role of the SNF2L/SLC7A11/GSH regulatory axis as a critical factor in modulating cellular responses to therapy.

### SNF2L regulates cancer cell sensitivity to APR-246 through SLC7A11

**Fig. 5:**
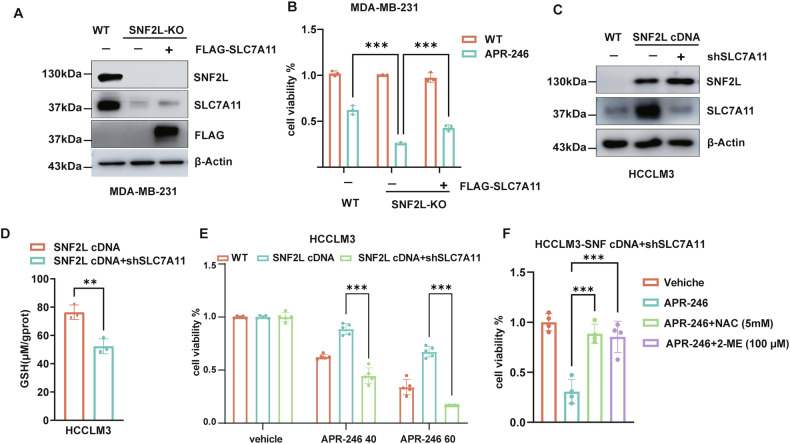
SNF2L regulates cancer cell sensitivity to APR-246 through SLC7A11. **A** Relative expression of SNF2L, SLC7A11 and FLAG-SLC7A11 proteins was analyzed by Western blotting in the indicated MDA-MB-231 cells, and β-actin was used as the loading control. **B** Cell viability was measured using the MTT assay in the indicated MDA-MB-231 cells after treatment with 20 µM APR-246 for 48 hours (n = 3). **C** Relative expression of SNF2L and SLC7A11 was analyzed by Western blotting in the indicated HCCLM3 cells, and β-actin was used as the loading control. **D** Relative GSH levels were measured in the indicated HCCLM3 cells (n = 3). **E** Cell viability was measured using the MTT assay in the indicated HCCLM3 cells after treatment with vehicle or 40 µM or 60 µM APR-246 for 48 hours (n = 5). **F** Cell viability was measured using the MTT assay in the indicated HCCLM3 cells after treatment with 40 µM APR-246 for 48 hours, with or without cotreatment of 5 mM NAC or 100 µM β-ME (n = 4). Error bars are the mean±SD, All data are representative of at least three biologically independent experiments. *p* values were calculated using two-tailed unpaired Student’s *t tests* (**B**, **D**, **E**) *or* one-way ANOVA with Tukey’s multiple comparisons test (**F**). *p < 0.05, **p < 0.01, ***p < 0.001.

To further elucidate whether SNF2L modulates the sensitivity of cancer cells to APR-246 through SLC7A11, we ectopically expressed SLC7A11 in SNF2L-deficient MDA-MB-231 cells (Fig. 5A, uncropped original western blots Fig. 5A). Remarkably, we observed that the ectopic expression of SLC7A11 partially rescued the cells from APR-246-mediated cell death (Fig. 5B). Conversely, we employed short hairpin RNA (shRNA)-mediated deletion to knock down SLC7A11 expression in HCCLM3 cells in which SNF2L was ectopically expressed (Fig. 5C, uncropped original western blots Fig. 5C). Interestingly, SLC7A11 knockdown decreased the GSH level in HCCLM3 cells (Fig. 5D). Furthermore, SLC7A11 knockdown resulted in a notable decrease in cell viability compared to that of control cells (Fig. 5E). Additionally, the administration of NAC and 2-ME markedly prevented the cell death induced by APR-246 (Fig. 5F), suggesting that the loss of SLC7A11 exacerbates oxidative stress in cancer cells. These results collectively highlight the critical role of SNF2L-SLC7A11 axis in mediating the response of cancer cells to APR-246. Further research is warranted to understand the underlying molecular mechanisms in which SNF2L regulated SLC7A11 expression.

### SNF2L enhances SLC7A11 transcription by regulating chromatin accessibility

**Fig. 6:**
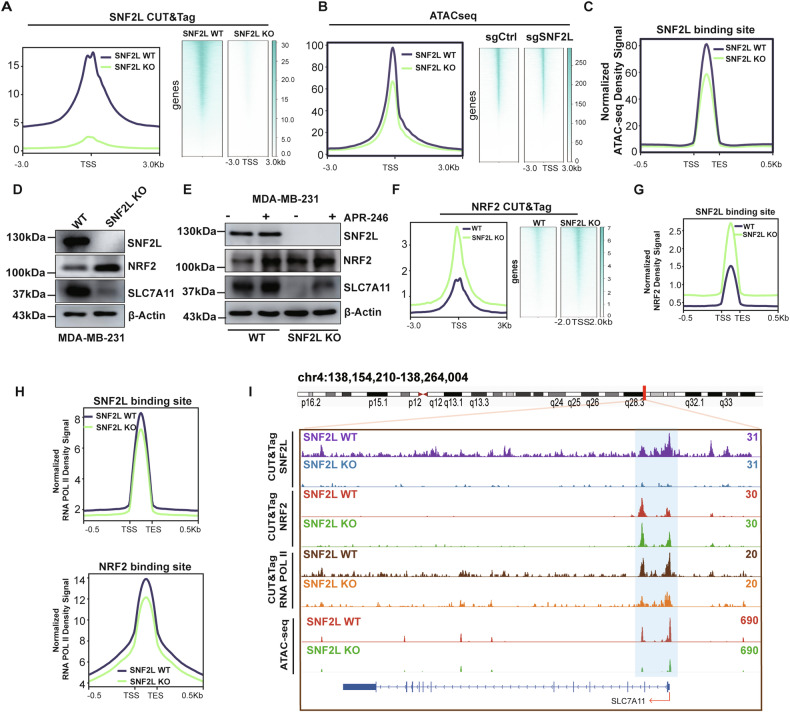
SNF2L increases SLC7A11 expression by regulating chromatin accessibility. **A** The enrichment of SNF2L peaks around the TSS (±3 kb) in SNF2L-deficient and parental MDA-MB-231 cells was identified by CUT&Tag. **B** The enrichment of SNF2L peaks around the TSS (±3 kb) in SNF2L-deficient and parental MDA-MB-231 cells was identified by ATAC-seq. **C** Normalized ATAC-seq density signals at SNF2L binding sites identified by CUT&Tag. **D** Relative expression of SNF2L, NRF2 and SLC7A11 proteins were analyzed by western blotting in SNF2L-deficient and parental MDA-MB-231 cells, and β-actin was used as the loading control. **E** Relative expression of SNF2L, NRF2 and SLC7A11 proteins were analyzed by western blotting in SNF2L-deficient and parental MDA-MB-231 cells after treatment with vehicle or 10 µM APR-246 for 12 hours. β-Actin was used as the loading control. **F** Enrichment of NRF2 peaks around the TSS (±3 kb) in SNF2L-deficient and parental MDA-MB-231 cells was identified by CUT&Tag. **G** Normalized NRF2 density signals at SNF2L binding sites identified by CUT&Tag. **H** Normalized RNA POL II density signals at the SNF2L and NRF2 binding sites identified by CUT&Tag. **I** The distribution of SNF2L, NRF2, and RNA POL II signaling peaks in the promoter region of SLC7A11 corresponded with the chromosomal region accessibility was identified by CUT&Tag and ATAC-seq.

Previous investigations have indicated that SNF2L binds to promoters and influences nucleosome positioning near the transcription start sites of genes [24]. To elucidate how SNF2L controls gene transcription via chromatin remodeling, we first conducted SNF2L Cleavage Under Targets and Tagmentation (CUT&Tag) assays to map genome-wide SNF2L binding sites in parental MDA-MB-231 cells with SNF2L- deficient cells serving as a negative control. CUT&Tag identified 25,690 different peaks in parental MDA-MB-231 cells compared to SNF2L-deficient cells. Notably, the majority of SNF2L binding sites (54.13%) were predominantly located within the promoter region, specifically within +/-1 kb of the transcription start sites (Fig. S4A). In contrast, no SNF2L binding signals were detected in SNF2L-deficient cells, indicating a complete loss of binding activity in the absence of SNF2L (Fig. 6A.I). This suggests that SNF2L plays a crucial role in gene regulation in cancer cells.

Given the critical role of SNF2L in gene transcriptional activation via chromatin remodeling, we investigated whether SNF2L regulates genome-wide chromatin accessibility. A transposase-accessible chromatin assay (ATAC-seq) was performed to assess genome-wide accessibility in SNF2L-deficient cells and parental MDA-MB-231 cells. Genome-wide analysis revealed 35,909 differentially accessible chromatin regions. Notably, after SNF2L deletion, chromatin accessibility was reduced across genome-wide regions (Fig. 6B) and SNF2L binding sites (Fig. 6C, I), indicating that SNF2L directly maintains a subset of open chromatin regions where SNF2L binding is abundant. Additionally, We also observed a strong positive correlation between differential RNA-seq profiles and ATAC-seq signals (Fig. S4B), indicating a close relationship between chromatin accessibility and gene expression. Furthermore, we investigated whether SNF2L interacts with the SLC7A11 promoter and promotes its transcription by enhancing local chromatin accessibility. Through an integrated analysis of the transcriptome, ATAC-seq, and CUT&Tag results, we identified SLC7A11 as one of 141 genes regulated by SNF2L via chromatin remodeling (Fig. S4C). These findings strongly suggest that SNF2L influences the transcription of SLC7A11 mRNA by modulating the accessibility of its chromatin environment.

To verify these findings, we disrupted the SNF2L binding site using CRISPR/Cas9 in MDA-MB-231 cells (Fig. S5A), where we observed a decrease in SLC7A11 expression (Fig. S5B). Notably, the loss of the SNF2L binding site significantly reduced the viability of MDA-MB-231 cells treated with APR-246 (Fig. S5C).

Studies have shown that transcription factors recruit coactivators and ATP-dependent nucleosome remodeling complexes to facilitate the recruitment of RNA polymerase II and the formation of preinitiation complexes at the promoters of target genes [25]. Our aim was to identify transcription factors involved in regulating the transcription of SLC7A11 via SNF2L-mediated chromatin remodeling. For this purpose, we used HOMER to search for transcription factor motifs in the ATAC-seq datasets. Nuclear factor erythroid 2-related factor 2 (NRF2), the master regulator of redox homeostasis, was identified in the ATAC-seq datasets (Fig. S4D). Next, We conducted a coimmunoprecipitation (Co-IP) assay to explore whether SNF2L interacts with NRF2. HEK293T cells were transfected with expression vectors containing FLAG- and HA-tags for SNF2L and NRF2, respectively. NRF2 was detected in the immunoprecipitated complexes of FLAG-tagged SNF2L (Fig. S4E, uncropped original western blots Figure S4E), and SNF2L was also detected in the immunoprecipitated complexes of HA-tagged NRF2 (Fig. S4F, uncropped original western blots Fig. S4F), indicating specific interactions between SNF2L and NRF2.

To elucidate the functional implications of the SNF2L-NRF2 interaction, we investigated NRF2 expression levels in SNF2L-deficient cells. Interestingly, we noted increased NRF2 expression but reduced SLC7A11 levels in SNF2L-deficient cells (Fig. 6D, uncropped original western blots Fig. 6D). Furthermore, following APR-246 treatment, NRF2 expression was upregulated in both wild-type and SNF2L-deficient cells, with only a slight increase in SLC7A11 gene expression noted in wild-type cells (Fig. 6E, uncropped original western blots Fig. 6E). This observation suggests the essential role of SNF2L in regulating SLC7A11 expression; even in its absence, complete restoration of SLC7A11 expression is not achieved despite the increase in NRF2 expression. In summary, these findings underscore the importance of the SNF2L-NRF2 interaction in maintaining cellular redox balance and its intricate role in the transcriptional initiation of the SLC7A11 gene.

To explore the mechanism by which NRF2 might regulate SLC7A11 transcription in cooperation with SNF2L, we conducted a genomic assay to examine genome-wide binding changes of NRF2 following SNF2L loss. The intensity of genome-wide NRF2 binding increased following SNF2L deletion (Fig. 6F), which was consistent with the western blot results. Remarkably, the normalized NRF2 density signal at SNF2L binding sites dramatically increased following SNF2L deletion (Fig. 6G). These results indicate that NRF2 might be compensatory binding to more sites in the absence of SNF2L, possibly attempting to maintain SLC7A11 transcription and redox homeostasis. However, the normalized RNA pol II density decreased at both the SNF2L and NRF2 binding sites throughout the genome (Fig. 6H), as well as at the SLC7A11 gene promoter region (Fig. 6I). These findings suggests that RNA pol II binding and transcription initiation are inhibited throughout the genome and SLC7A11 promoter despite the increased NRF2 binding.

The results provide significant insights into the complex regulatory dynamics between NRF2 and SNF2L. While NRF2 increases its binding to the genome in response to SNF2L loss, this compensatory mechanism appears insufficient to sustain the recruitment of RNA pol II and the subsequent transcriptional activity of SLC7A11. This suggests that SNF2L plays a critical role in facilitating the proper chromatin structure required for effective transcriptional initiation by RNA pol II. Together, we propose a new working model in which NRF2 depends on SNF2L to enhance chromatin accessibility and initiate the transcription of the SLC7A11 gene to maintain redox homeostasis.

## Discussion

In our study, we revealed the pivotal role of SNF2L, a chromatin remodeling factor, in maintaining redox homeostasis. Our findings revealed that SNF2L deficiency significantly impacts cancer cell viability by decreasing intracellular GSH levels via metabolic reprogramming. Furthermore, we found that APR-246, known for reducing intracellular GSH levels and reactivating mutant p53, induces oxidative stress and accelerates cell death in SNF2L-deficient cells. Additionally, SNF2L deficiency reduces SLC7A11 transcriptional activity, impairing cystine uptake in cells. Finally, we established a critical interaction between the transcription factor NRF2, a key regulator of the antioxidant response, and SNF2L. We demonstrated that NRF2 recruits SNF2L to the promoter region of the SLC7A11 gene, thereby enhancing chromatin accessibility and augmenting the expression of SLC7A11 and GSH biosynthesis, thereby increasing antioxidant capacity.

Previous research has shown that chromatin remodeling by the SWI/SNF factor ARID1A can deplete GSH and increase ROS levels by facilitating NRF2-mediated transcription of SLC7A11. This process induces oxidative stress and promotes cell death, especially in cancer cells with compromised antioxidant defenses [12]. In our study, we further elucidated the relationship between chromatin dynamics and cellular antioxidant responses. Our results demonstrated that the loss of SNF2L led to a significant increase in NRF2 expression. However, this increase in NRF2 expression did not restore SLC7A11 levels in SNF2L-deficient cells, highlighting the critical role of SNF2L in the transcription of SLC7A11. Based on these findings, we hypothesize that ARID1A’s regulation of SLC7A11 transcription is dependent on SNF2L. Further studies are required to investigate the Interplay between ARID1A and SNF2L.

This finding suggested that while NRF2 upregulation is a compensatory response to oxidative stress in SNF2L-deficient cells, the absence of SNF2L impairs the transcriptional machinery necessary for SLC7A11 expression. This highlights a more complex regulatory network in which SNF2L plays a crucial role beyond chromatin remodeling, potentially involving direct or indirect interactions with other transcription factors or coregulators specific to SLC7A11. Exploring these interactions has significant implications for cancer biology and therapy. Since NRF2 and SLC7A11 are vital for cellular antioxidant responses and often dysregulated in cancers, understanding the precise role of SNF2L could identify novel therapeutic targets. For example, targeting specific components of the chromatin remodeling machinery involved in SLC7A11 transcription may enhance the efficacy of treatments designed to modulate oxidative stress in cancer cells.

Additionally, we investigated the effects of APR-246, a small molecule that reactivates mutant p53 and has shown potential in inducing cancer cell death. APR-246 is converted into the active compound MQ, which binds to and reactivates cysteine residues in mutant p53, triggering apoptosis in p53-mutant cancer cells. Recent studies suggest that APR-246 may also induce cell death by depleting intracellular GSH levels. Our findings demonstrate that SNF2L deficiency increases cancer cell susceptibility to APR-246. Specifically, SNF2L-deficient cells, already exhibiting reduced SLC7A11 transcription and lower GSH levels, experience heightened oxidative stress upon APR-246 treatment. This suggests a synthetic lethal interaction between SNF2L loss and APR-246 treatment, where the combined effect leads to increased oxidative stress and enhanced cell death. More importantly, our findings indicate that the sensitivity of SNF2L-deficient cells to APR-246 is independent of mutant p53, consistent with previous studies that suggest APR-246 exerts its anticancer effects through multiple mechanisms beyond p53 reactivation [9, 26]. This finding is particularly intriguing because it indicates that alternative pathways or mechanisms may contribute to the efficacy of APR246 in these cells. Further research could focus on identifying these pathways and determining how SNF2L deficiency interacts with other cellular processes to influence drug sensitivity. Given that GSH is derived from cysteine, SNF2L’s regulation of SLC7A11 may not only impact oxidative stress but also intersect with broader metabolic pathways. Cancer cells with low SNF2L expression may exhibit impaired metabolic adaptability, making them more vulnerable to therapies that disrupt redox balance or metabolic function.

Our findings have significant therapeutic implications for cancer treatment. Cancer cells with low SNF2L expression, characterized by decreased GSH levels and elevated ROS, may be particularly susceptible to ROS-inducing drugs such as APR-246. This vulnerability indicates that targeting the SNF2L/SLC7A11 axis could offer an effective therapeutic strategy, especially for patients who are resistant to conventional chemotherapy and have limited treatment options. In tumors with pre-existing mutations or deficiencies in antioxidant pathways, such as those involving ARID1A or NRF2/Keap1, inhibiting SNF2L could induce synthetic lethality. Thus, targeting the SNF2L/SLC7A11 axis holds promise for advancing personalized cancer therapies, particularly in subtypes with low SNF2L expression and high oxidative stress.

However, this study has several limitations. Most findings are derived from cell models, which may not fully replicate the complex tumor microenvironment in vivo. Therefore, further validation through mouse models is essential to assess the efficacy and safety of targeting the SNF2L/SLC7A11 axis in a more physiologically relevant context. Additionally, the potential side-effects of increasing oxidative stress, particularly in healthy tissues, must be thoroughly evaluated before advancing to preclinical trials.

Future studies should focus on elucidating the precise molecular mechanisms by which SNF2L regulates the SLC7A11/GSH axis and its interactions with various oxidative stress pathways. Understanding these mechanisms will provide valuable insights into the role of SNF2L in cellular redox balance and its implications for cancer therapy. Additionally, research should explore the potential of combining SNF2L inhibition with other therapeutic strategies, such as immunotherapy or targeted therapies. Investigating these combinations could enhance treatment efficacy, particularly in cancer patients exhibiting drug resistance. Moreover, future investigations might include the development of specific inhibitors or modulators of SNF2L, assessing its effects in both preclinical models and clinical trials.

## Methods

### Cell lines

MDA-MB-231 (HTB-26), 293T (CRL-3216), NCI-H1299 (CRL-5803), and HT-1080 (CCL-121) cells were obtained from ATCC. HuH-7 (JCRB0403) was obtained from JCRB. HCCLM3 cells were obtained from the Liver Cancer Institute of Zhongshan Hospital, Fudan University. The cells were cultured in Dulbecco’s modified Eagle’s high glucose medium (DMEM) supplemented with 10% FBS, 1% penicillin/streptomycin, and 1% L-glutamine in an incubator at 37 °C and 5% CO_2_. Commonly misidentified cell lines listed in the Cellosaurus database were not used in this study.

### Constructs and reagents

SNF2L-deficient cell lines were established using CRISPR/Cas9 technology. The sgRNAs were cloned and inserted into the lentiviral LentiCRISPR v2 vector from Addgene (plasmid #52961). shRNA sequences were designed, annealed, and ligated into the AgeI/EcoRI-digested pLKO.1 vector. Gene knockdown cell lines were generated by shRNA lentivirus infection and puromycin selection. The pEnCMV-SNF2L-3xFLAG, pEnCMV-SLC7A11-3xFLAG and pEnCMV-NRF2-3xHA plasmids were obtained from MiaoLingBio, China. The gRNAs and shRNA sequences used in this study are listed in Supplementary Table S4. APR-246 (#HY-19980), ferrostatin-1 (#HY-100579), acetylcysteine (#HY-B0215), and DCFH-DA (#HY-D0940) were purchased from MedChemExpress. Chloroquine (#T8689), necrostatin-1 (#T1847), and Z-VAD-FMK (#T7020) were obtained from TargetMol.

### Generation of lentiviral particles

Plasmids were prepared using the EndoFree Plasmid Maxi Kit (#12362, Qiagen) according to the manufacturer’s instructions. Lentiviral particles were produced by cotransfecting 293 T cells with the transfer plasmid (1500 ng), packaging plasmid (psPAX2, 800 ng), or envelope plasmid (pMD2. G, 400 ng) using polyethylenimine linear 40000 (PEI) (#40816ES02, Yeasen, Shanghai, China). Transfection was carried out in 6-well culture plates with 80% confluent cells. The DNA-PEI complexes were prepared following the manufacturer’s protocol and added dropwise to the cells. After 6 hours, the medium was replaced with fresh DMEM (#209011, NEST, China) containing 10% FBS (#P30-3306, PAN-Biotech). 72 hours posttransfection, the lentiviral supernatant was collected and filtered through a 0.45 µm PVDF filter (#SLHV033RB, Millipore) to remove cell debris and aliquoted for storage at −80 °C.

### Generation of Stable cell lines cell lines

Cell lines were transduced with the lentiviral supernatant containing 0.8 µg/mL polybrene. 12 hours after transduction, the supernatant was removed, and the cells were selected with puromycin (1 µg/mL) for 48 hours to ensure successful integration of the lentiviral constructs. CRISPR/Cas9 knockout Single cell colony was generated by limiting dilution. Single cells were seeded in 96-well palte and expanded for about 2 weeks. Single cell colonies were screened by western blotting.

### Reduced GSH and ROS detection

Reduced GSH was detected using a reduced GSH colorimetric assay kit (#EEA020, Thermo Fisher Scientific) according to the manufacturer’s protocol. Briefly, cells were cultured and sonicated in cold homogenization buffer (1x PBS, 0.01 M, pH 7.4). 100 μL of supernatant was mixed with 100 μL of acidic reagent and centrifuged at 4500 × *g* for 10 minutes, after which the supernatant was collected for detection. Then, 100 μL of the supernatant and GSH standard were added to a 96-well plate and mixed with 25 μL of DTNB and 100 μL of phosphate solution. The OD value was determined at 405 nm with a microplate reader. The protein concentration of the supernatant was determined as an internal control. Reduced GSH levels were normalized against protein concentration. The level of ROS in cells was measured using the fluorescent probe H2DCFDA (#HY-D0940, MedChemExpress). Briefly, cells were cultured in a 96-well plate and incubated with 10 µM DCFH-DA for 30 minutes at 37 °C in the dark. After washing with PBS, fluorescence intensity (Ex/Em = 490/535 nm) was measured using an Envision Multi-label plate reader (PerkinElmer). The relative ROS levels were normalized to cell numbers and compared to wild-type cells.

### Colony formation assay

The effect of small molecule drug treatment on cell viability was measured by colony formation assays. Briefly, 2000 cells were seeded in 6-well plates and treated with the indicated concentrations of small molecule drugs for 10–14 days, followed by fixation in 50% methanol with 0.01% crystal violet for 20 minutes. Cell colony area percentage was calculated using ImageJ software.

### Western blotting

Cells were lysed at 4 °C in PIPA buffer (20 mM Tris, pH 7.5; 150 mM NaCl; 1% Triton X-100) supplemented with a 1× protease inhibitor cocktail (#P0013, Beyotime, China). The protein concentration was determined using a BCA Protein Assay Kit (#HRX0119, FUJIAN HERUI Biotech, China). 20 µg of protein per sample was boiled, separated by SDS‒PAGE, and then transferred to polyvinylidene difluoride (PVDF) membranes. The membranes were blocked with 5% nonfat milk in Tris-buffered saline for 1 hour at room temperature and incubated with primary antibody overnight at 4 °C with gentle shaking. The blots were then incubated with peroxidase-conjugated secondary antibody for 1 hour at room temperature. Target proteins were detected using an Ultra High Sensitivity ECL Kit (#GK10008, GLPBIO). The primary antibodies and concentrations used were as follows: anti-SLC7A11 (1:2500, #12691, Cell Signaling Technology), anti-SNF2L (1:2000, #61465, Active Motif), anti-β-actin (1:20000, #AC026, ABclonal), anti-NRF2 (1:1000, #80593-1-RR, Proteintech), and anti-P53 (1:5000; #60283-2-Ig, Proteintech).

### RNA isolation and qRT‒PCR

Total RNA was purified using TRIzol reagent (#15596026, Invitrogen) according to the manufacturer’s protocol. Briefly, 1 μg of purified RNA was reverse transcribed with a HiScript III 1st-strand cDNA synthesis kit with gDNA wiper (#R312, Vazyme). Real-time PCR was performed in triplicate using ChamQ Universal SYBR qPCR Master Mix (#Q711, Vazyme). GAPDH was used as an internal control. Gene expression levels were quantified using the ΔΔCt method. All primers used are listed in Supplementary Table S4.

### RNA-seq and data analysis

Total RNA was purified using RNeasy Mini kits (#74106, QIAGEN), subjected to gene expression profiling via mRNA-seq with the Illumina NovaSeq 6000 platform and sequenced to generate 150-bp paired-end reads. The raw sequencing reads were subjected to quality control using FastQC (version 0.11.9). Low-quality bases and adapter sequences were trimmed using TrimGalore (version 0.6.7). Cleaned reads were aligned to the reference genome (hg38) using HISAT2 (version 2.2.1). Aligned reads were quantified at the gene level using featureCounts (version 2.0.1). Differential gene expression analysis was performed using DESeq2 (version 1.42.1) in R. Genes with an adjusted p value < 0.05 were considered significantly differentially expressed.

### Cystine uptake assay

Cystine uptake was determined using a cystine uptake assay kit (#UP50, Dojindo Laboratories) according to the manufacturer’s protocol with optimizations. Cells were seeded in a 96-well microplate and incubated overnight. After washing three times with pre-warmed cystine-free, serum-free medium (#21013024, Thermo Fisher Scientific), incubate for 5 minutes in the same medium. Treat with pre-warmed CA uptake solution (or blank medium) for 30 minutes, then wash with ice-cold PBS. Add methanol and the working solution, incubate for 30 minutes, and measure fluorescence intensity (Ex/Em = 490/535 nm) using an Envision Multi-label plate reader (PerkinElmer). Fluorescence intensity was normalized to cell numbers using Hoechst 33342, and the relative cystine uptake ratios in SNF2L KD/KO cells were compared to wildtype cells.

### ATAC-seq and data analysis

ATAC-seq was performed using the High-Sensitivity Open Chromatin Profile Kit 2.0 (N248, Novoprotein Scientific) according to the manufacturer’s protocol. Briefly, cells were collected, and nuclei were lysed using lysis buffer (10 mM Tris-HCl, pH 7.5; 10 mM NaCl; 3 mM MgCl2; 0.1% NP-40; 0.1% NP-40; 0. 01% digitonin) isolated. The nuclei were then resuspended in PBS supplemented with 0.1% BSA. For each sample, 50,000 nuclei were used for the transposition reaction. Nuclei were centrifuged for 10 minutes at 4 °C at 500 × g and resuspended in transposition mix containing 2 μl of transposome mix and 25 μl of TD buffer in a total volume of 50 μl. The transposition reaction was carried out for 30 minutes at 37 °C in a thermal cycler. After transposition, the DNA was purified using tagment DNA extract beads. The purified DNA was PCR amplified using 2xHiFi AmpliMix and indexed primers. The PCR conditions were as follows: 72 °C for 3 minutes, 98 °C for 30 seconds, followed by 12 cycles of 98 °C for 15 seconds, 63 °C for 8 seconds, and 72 °C for 3 seconds, with a final extension at 72 °C for 2 minutes. Amplified libraries were purified with Agencourt AMPure XP beads (#A63880, Beckman Coulter) and quantified with a Qubit dsDNA HS Assay Kit (#Q33230,Thermo Fisher Scientific). Sequencing was performed on an Illumina NovaSeq 6000 platform with paired-end 150-bp reads. Sequencing reads were trimmed for adapter sequences and low-quality bases using TrimGalore (version 0.6.7). Trimmed reads were aligned to the reference genome (hg38) using Bowtie2 (version 2.5.3) with default parameters. Duplicate reads were removed using Picard Tools (version 2.23.4). Peaks were called using MACS2 (version 2.2.9.1) with a q-value cut-off of 0.01. The resulting peaks were annotated using HOMER (version 4.11) and visualized using IGV (version 2.11.3). Differential accessibility analysis was performed using DiffBind (version 3.12.0). Differential peaks were identified using EdgeR (version 4.0.16).

### CUT&Tag sequencing and data analysis

CUT&Tag sequencing was performed using the Hyperactive Universal CUT&Tag Assay Kit (TD903, Vazyme) according to the manufacturer’s protocol. Briefly, Concanavalin A beads were prepared by washing 10 μL of bead slurry with 100 μL of binding buffer three times. The beads were resuspended in 10 μl of binding buffer. A total of 100,000 cells were used for each CUT&Tag experiment. The cells were resuspended in 100 μl of washing buffer and mixed with the prepared concanavalin A beads. The mixture was incubated for 10 minutes at room temperature with gentle rotation to allow the cells to bind to the beads. The cells were then incubated with 1 μg of primary antibody in 50 μl of antibody buffer at 4 °C overnight without rotation. The cells were incubated with secondary antibody in 50 μL of Dig-Wash Buffer at room temperature for 1 hour with gentle rotation. The cells were washed three times with Dig-Wash Buffer to remove unbound antibodies. The cells were incubated with 0.04 μM pA/G-Tnp transposome in 100 μL of Dig-300 Buffer at room temperature for 1 hour with gentle rotation. After tagmentation, the cells were washed three times with Dig-300 Buffer. Tagmentation was activated by adding 50 µl of tagmentation buffer and incubating the mixture at 37 °C. for 1 hour. DNA was extracted using DNA extraction beads. The purified DNA was PCR amplified using 2XCAM PCR Mix and indexed primers. The PCR conditions were as follows: 72 °C for 3 minutes, 95 °C for 3 minutes, followed by 11 cycles of 98 °C for 10 seconds, 60 °C for 5 seconds, and 72 °C for 1 minute, with a final extension at 72 °C for 1 minute. Amplified libraries were purified using VAHTS DNA Clean Beads (#N411, Vazyme) and quantified using the Qubit dsDNA HS Assay Kit (Thermo Fisher Scientific). Sequencing was performed on an Illumina NovaSeq 6000 platform with paired-end 150-bp reads. Sequencing reads were trimmed for adapter sequences and low-quality bases using TrimGalore (version 0.6.7). Trimmed reads were aligned to the reference genome (hg38) using Bowtie2 (version 2.5.3) with default parameters. Duplicate reads were removed using Picard Tools (version 2.23.4). Peaks were called using SEACR_1.3. The resulting peaks were visualized using IGV (version 2.11.3). Differential peak analysis was performed using DiffBind (version 3.12.0). Differential peaks were identified using EdgeR (version 4.0.16).

### Coimmunoprecipitation (Co-IP)

A total of 5 × 10^6^ 293 T cells were seeded in 60 mm dishes. The following day, the cells were transfected with FLAG-SNF2L and HA-NRF2 plasmids and then incubated for 24 hours. Afterward, the cells were harvested and sonicated in RIPA buffer containing a proteinase inhibitor. Immunoprecipitation was performed with 1 mg of total protein using anti-HA and anti-FLAG antibodies conjugated with Protein A/G Dynabeads overnight with gentle rotation. On the second day, the beads were washed five times with RIPA buffer. The eluted samples were analysed by western blotting using anti-HA and anti-FLAG antibodies.

### Mass spectrometry and data analysis

Mass spectrometry was performed as described previously [27]. Briefly, cells were lysed by adding five cell pellet volumes of lysis buffer (100 mM triethyl-ammonium bicarbonate and 1% SDS). Lysates were centrifuged at 16,000 × *g* for 10 minutes at 4 °C, and the protein concentration of the supernatant was determined using BCA assays. A total of 100 μg of protein from each sample was precipitated using a 5-fold volume of ice-cold acetone. The pellet was resolved in 100 mM triethyl-ammonium bicarbonate (TEAB) and subjected to tryptic digestion after reduction with dithiothreitol (DTT) (10 mM) at 60 °C for 30 minutes and alkylation with iodoacetamide (IAA) (40 mM) in the dark for 10 minutes. Trypsin was added to the proteins at a 1:50 (w:w) ratio, and the mixture was incubated at 37 °C overnight. The peptides were labeled with a set of TMT-6plex isobaric tags following the manufacturer’s instructions. The sample labels used were as follows: Con_1, 126; Con_2, 127; Con_3, 128; KO_1, 129 N; KO_2, 130; and KO_3, 131. The labeled peptides were then mixed, desalted, and vacuum-dried. Using a high-pH reversed-phase peptide fractionation kit (Thermo Fisher Scientific, #84868), the labeled peptides were fractionated into eight fractions and dried by vacuum centrifugation for further processing. For LC‒MS/MS analysis, the prefractionated peptides were dissolved in 0.1% formic acid (solvent A) and directly loaded onto a reversed-phase analytical column (Acclaim PepMap C18, 75 μm × 25 cm). The gradient comprised an increase from 2% to 30% solvent B (0.1% formic acid in 98% acetonitrile) over 50 minutes, 30% to 50% in 5 minutes, and an increase to 80% in 1 minute, followed by holding at 80% for the last 4 minutes, all at a constant flow rate of 200 nl/min on an EASY-nLC 1200 UPLC system. The peptides were subjected to an NSI source followed by tandem mass spectrometry (MS/MS) in an Orbitrap Exploris 480 MS coupled online to the UPLC. The spray voltage was set to 2.3 kV, the funnel RF level was set to 50, and the capillary temperature was heated to 320 °C. For the DDA experiments, the full MS resolution was set to 60,000 at m/z 200, and the full MS AGC target was 300% with an IT of 25 ms. The mass range was set to 350–1600. The AGC target value for fragment spectra was set to 200%, with a resolution of 15,000 and injection times of 50 ms and Top12. The intensity threshold was maintained at 2E5. The isolation width was set to 1.6 m/z, and the normalized collision energy was set to 30%. Mass spectrometric files were processed using MaxQuant 2.0.3.0, and the data were searched against the human UniProt database consisting of reviewed canonical sequences (total entry 20594, downloaded in April 2022). TMT 6-plex-based MS2 reporter ion quantification was chosen, with the reporter mass tolerance set to 0.003 Da and the PIF (precursor intensity fraction) filter value set to 0.5. The tolerances for the parent mass were set to 10 ppm, with the fragment mass tolerance set to 0.02 Da. Specific tryptic cleavages were selected, allowing for a maximum of 2 missed cleavages. A maximum of 5 modifications per peptide were allowed. The minimal peptide length was set to 7. The maximum false discovery rate (FDR) for protein, peptide, and PSM identification was set to <0.01. The protein expression values obtained from MaxQuant were normalized using median normalization by column and then log2 transformed before differential expression analysis.

### Nuclear magnetic resonance metabolomics

NMR metabolomics was performed as described previously [28]. Briefly, the cell samples were transferred to a tube (containing 1.4 mm ceramic spheres; MP Biomedicals LLC, Santa Ana, CA, USA) to obtain metabolites, and then 400 μL of ice-cold methanol and 200 μL of Milli-Q H_2_O were added to each tube for homogenization by a FastPrep-24 tissue homogenizer (MP Biomedicals LLC, Santa Ana, CA, USA). After centrifugation at 12,000 rpm for 30 minutes at 4 °C, the supernatant was collected and evaporated to obtain a dry metabolite pellet (Eppendorf Concentrator Plus, Hamburg, Germany). For the NMR experiments, the samples were redissolved in 500 μL of NMR buffer (5 mM Tris (trimethylsilyl) phosphate (TMSP), 0.08 M Na_2_HPO4, 0.04% (w/v) NaN3 dissolved in D_2_O, 8 M HCl, and 5 M NaOH were used to adjust the pH to 7.4). NMR spectra were recorded and processed using MATLAB 2014a to obtain aligned and normalized datasets as previously described [29]. Briefly, Bruker Avance III HD 600-MHz NMR spectrometer with a TXI probe head was used for the NMR experiments for 1H NMR metabolic profiling and analysis. To collect 1H 1D NMR spectra with water suppression, the Carr-Purcell-Meiboom-Gill (CPMG) pulse sequence was utilized (cpmgpr1d, 512 scans, 73,728 points in F1, 12019.230 Hz spectral width, 1024 transients, recycle delay 4 s). Chemical shifts were referenced to the internal 3-(trimethylsilyl) propionic acid-2,2,3,3-d4 sodium salt (TSP) signal for 1H 1D spectra. The spectral quality in each spectrum was assessed directly after acquisition, based on the shape of the internal TSP signal. NMR spectral data were processed in Bruker Topspin version 4.0.2 using one-dimensional exponential window multiplication of the Free Induction Decay (FID), Fourier transformation, and phase correction. After the NMR data were imported into Matlab2014a. TSP was used as the internal standard for chemical-shift referencing (set to 0 ppm); regions around the water, TSP, and methanol signals were excluded. Reduced spectra and normalized spectra were generated by Matlab2014a, among which the normalized spectra were used to quantify metabolites by signal integration. In this study, we conducted non-targeted metabolomics analysis and utilized reduced spectra to identify differential metabolites by searching through a database of approximately 500 metabolites. Metabolic identification and annotation databases are primarily derived from The Human Metabolome Database (HDMB). For each quantified metabolite, a characteristic peak without interfering signals was selected, the start and end points limiting the range of the peak were defined to integrate the area of the peak by summing the values for each point.

### Statistics and reproducibility

All cell culture experiments were conducted with at least three independent repeats. For western blots, the experiments were repeated at least three times, and representative data were shown. Results are presented as means ± SD. Statistical analysis was performed using a two-tailed unpaired Student’s t-test, one-way ANOVA with Tukey’s multiple comparisons test, or two-way ANOVA with Tukey’s multiple comparisons test. Correlation between two groups was assessed using Pearson’s test. Statistically significant differences are indicated as follows: *p < 0.05, **p < 0.01, ***p < 0.001.

## Supplementary information

figure legend for supplementary figure

FigS1. SNF2L deficiency drives the reprogramming of GSH metabolism.

FigS2. SNF2L maintains GSH homeostasis by regulating SLC7A11 expression.

FigS3. SNF2L maintains GSH homeostasis in primary breast cancer cell.

FigS4. SNF2L increases SLC7A11 expression by regulating chromatin accessibility.

FigS5. SNF2L enhances SLC7A11 expression by binding to its promoter.

Supplementary Table

uncropped original western blots

## Data Availability

The NGS sequencing data supporting the findings of this study have been deposited in the SRA database with accession numbers PRJNA1119646 and PRJNA1119922. The mass spectrometry proteomics data has been deposited in the ProteomeXchange Consortium through the PRIDE partner repository, with the dataset identifier PXD052912.
