# Supplementary material for: SNF2L maintains glutathione homeostasis by initiating SLC7A11 transcription through chromatin remodeling
Source: Cell Death Dis. 2024 Nov 12;15(11):820. doi: 10.1038/s41419-024-07221-4 (PMC11557580; doi:10.1038/s41419-024-07221-4)
Supplement: Supplementary file 1 — figure legend for supplementary figure [file 41419_2024_7221_MOESM1_ESM.docx]

**FigS1. SNF2L deficiency drives the reprogramming of GSH metabolism.**

1. SNF2L protein expression between primary tumor and normal tissues of multiple cancers based on the CPTAC database.(B) The protein level of SNF2L in Huh-7 cells was measured by western blotting. β-Actin was used as the loading control. (C) Cell growth was measured by the MTT assay following SNF2L knockout in Huh-7 cells. (D) Colony formation of Huh-7 cells was measured. (E) Reduced spectra based on normalized NMR spectra of WT and SNF2L-KO cells. (F) Score plots of PLS−DA of WT and SNF2L-KO cells.. * *p* < 0.05, ** *p* < 0.01, *** *p* < 0.001.

**FigS2. SNF2L maintains GSH homeostasis by regulating SLC7A11 expression.**

(A) The relative expression of SNF2L and SLC7A11 proteins was analyzed using Western blotting in H1299 cells infected with a gradient of lentivirus expressing SNF2L. β-Actin served as the loading control. (B) Relative cystine uptake capacity was measured in parental HCCLM3 cells and HCCLM3 cells stably expressing SNF2L (n = 3).

**FigS3. SNF2L maintains GSH homeostasis in primary breast cancer cell.**

(A)The relative expression of SNF2L and SLC7A11 proteins was analyzed using Western blotting in primary breast cancer cell infected with lentivirus expressing shSNF2L. β-Actin served as the loading control. (B) Relative GSH levels were measured in SNF2L knockdown and parental primary breast cancer cell (n = 3). (C) Cell viability was measured using the MTT assay in SNF2L knockdown and parental primary breast cancer cell after treatment with 5 µM APR-246 for 24 hours (n = 3).

**FigS4.** **SNF2L increases SLC7A11 expression by regulating chromatin**

**accessibility.**

(A)The genome-wide distribution of SNF2L peaks identified by CUT&Tag. (B) Analysis of the correlation between RNA-seq and ATAC-seq promoter peaks, Correlation was evaluated using Pearson’s test. (C) Venn diagrams showing the number of studies identified by the databases (RNA-seq, ATAC-seq and CUT&Tag). (D) HOMER motif analysis with ATAC-seq of different peaks. (E, F) The protein levels of SNF2L and SLC7A11 were detected by western blotting in 293T cells.

**FigS5.** **SNF2L enhances SLC7A11 expression by binding to its promoter.**

(A) Diagram illustrating the sgRNA targeting site in the promoter region of the SLC7A11 gene. (B) The relative expression level of SLC7A11 protein was analyzed by Western blotting in MDA-MB-231 cells. β-Actin was used as the loading control. (C) Cell viability in MDA-MB-231 cells was measured using the MTT assay after treatment with 20 µM APR-246 for 24 hours (n = 3).
