# Supplementary figures and images for: SNF2L maintains glutathione homeostasis by initiating SLC7A11 transcription through chromatin remodeling
Source: Cell Death Dis. 2024 Nov 12;15(11):820. doi: 10.1038/s41419-024-07221-4 (PMC11557580; doi:10.1038/s41419-024-07221-4)

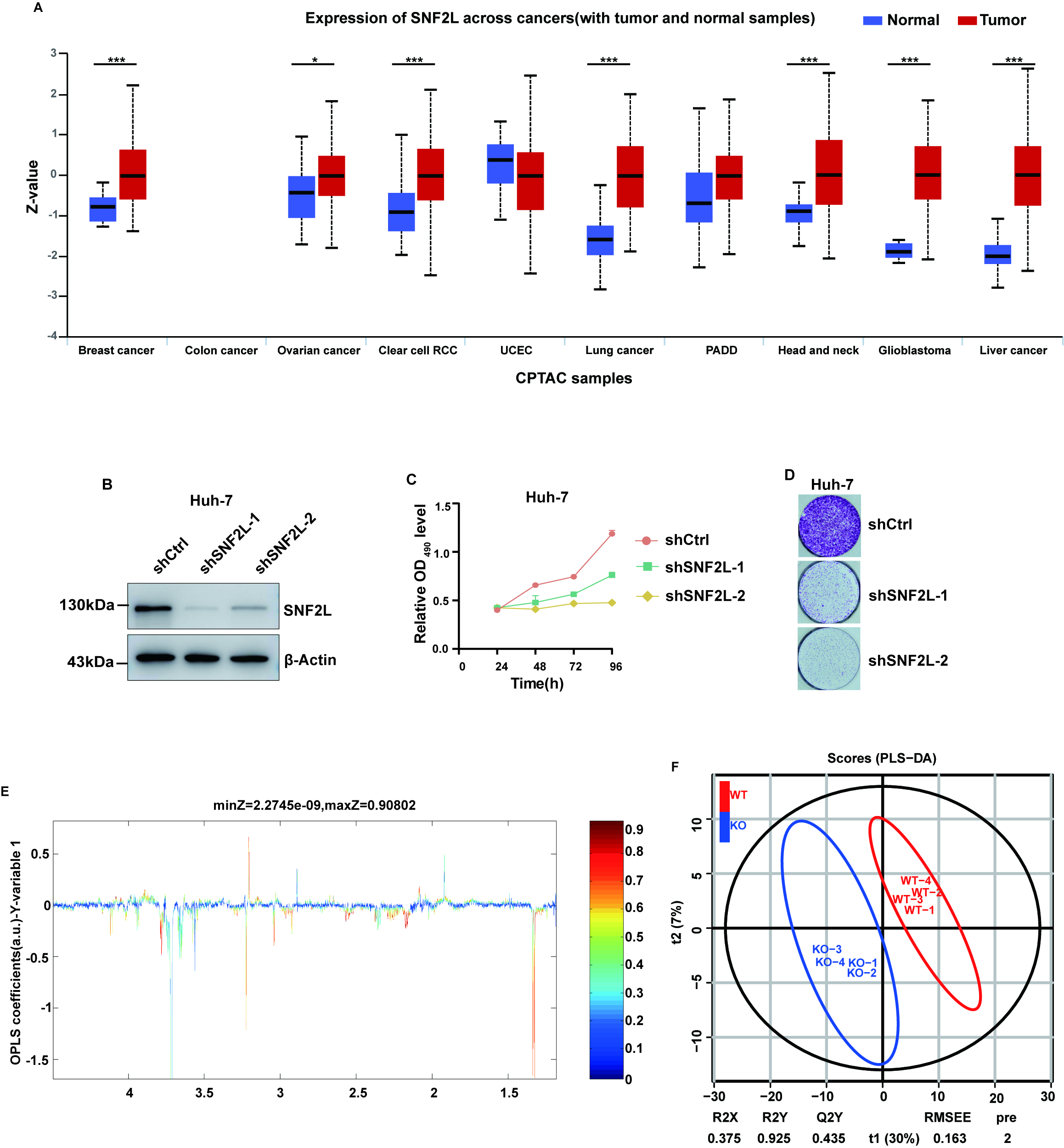

Supplement: Supplementary file 2 — FigS1. SNF2L deficiency drives the reprogramming of GSH metabolism. [file 41419_2024_7221_MOESM2_ESM.tif]

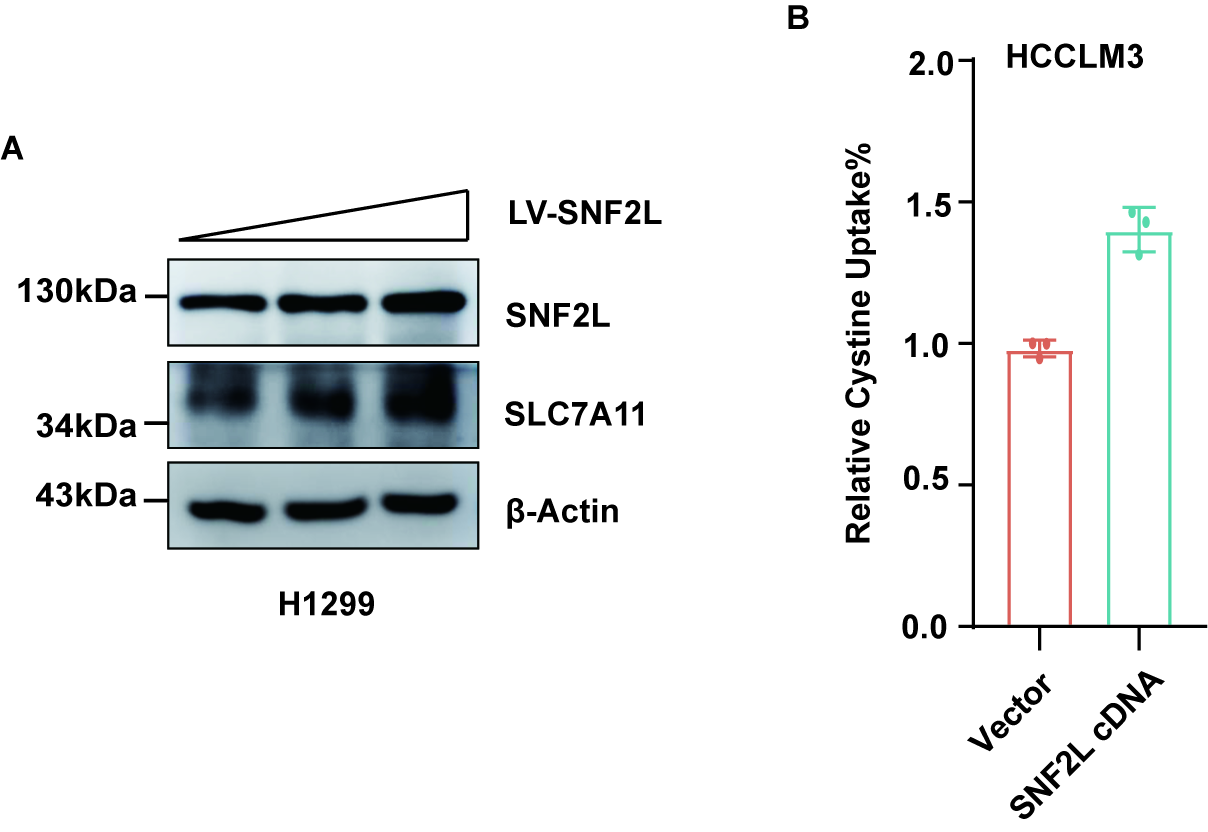

Supplement: Supplementary file 3 — FigS2. SNF2L maintains GSH homeostasis by regulating SLC7A11 expression. [file 41419_2024_7221_MOESM3_ESM.tif]

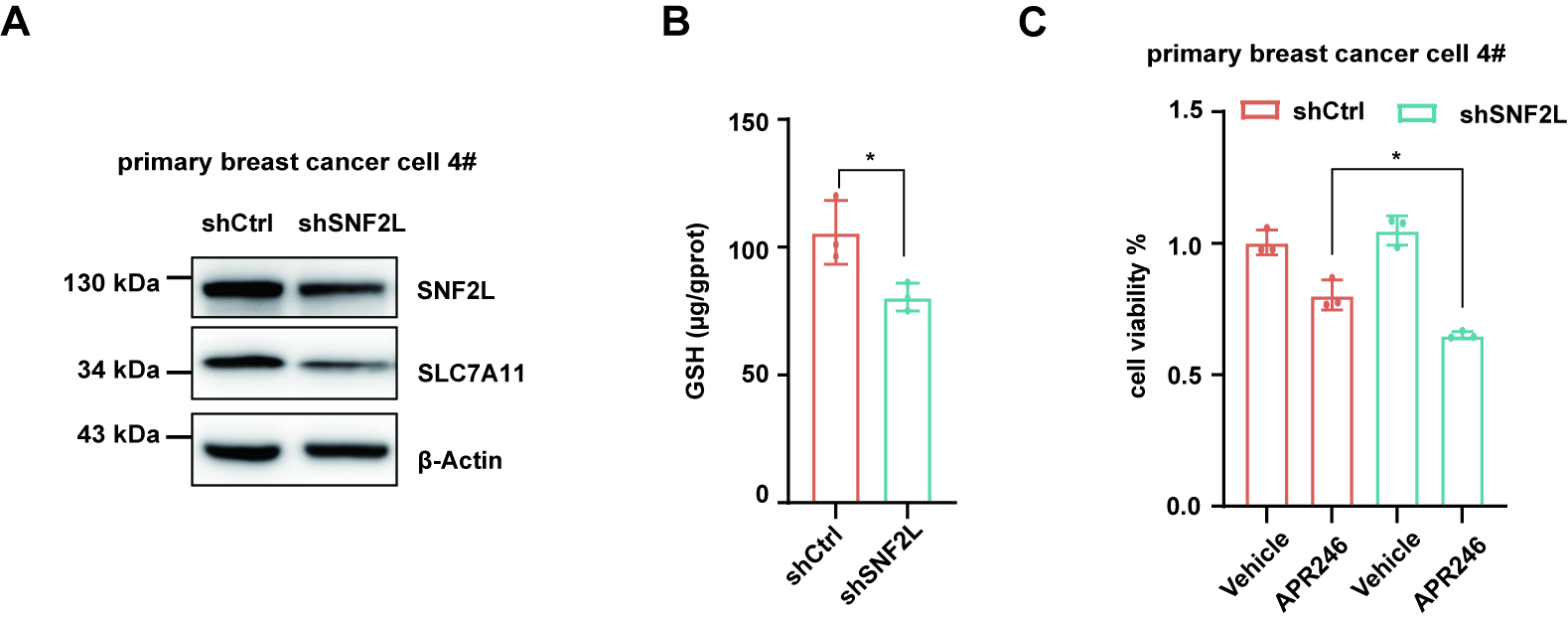

Supplement: Supplementary file 4 — FigS3. SNF2L maintains GSH homeostasis in primary breast cancer cell. [file 41419_2024_7221_MOESM4_ESM.tif]

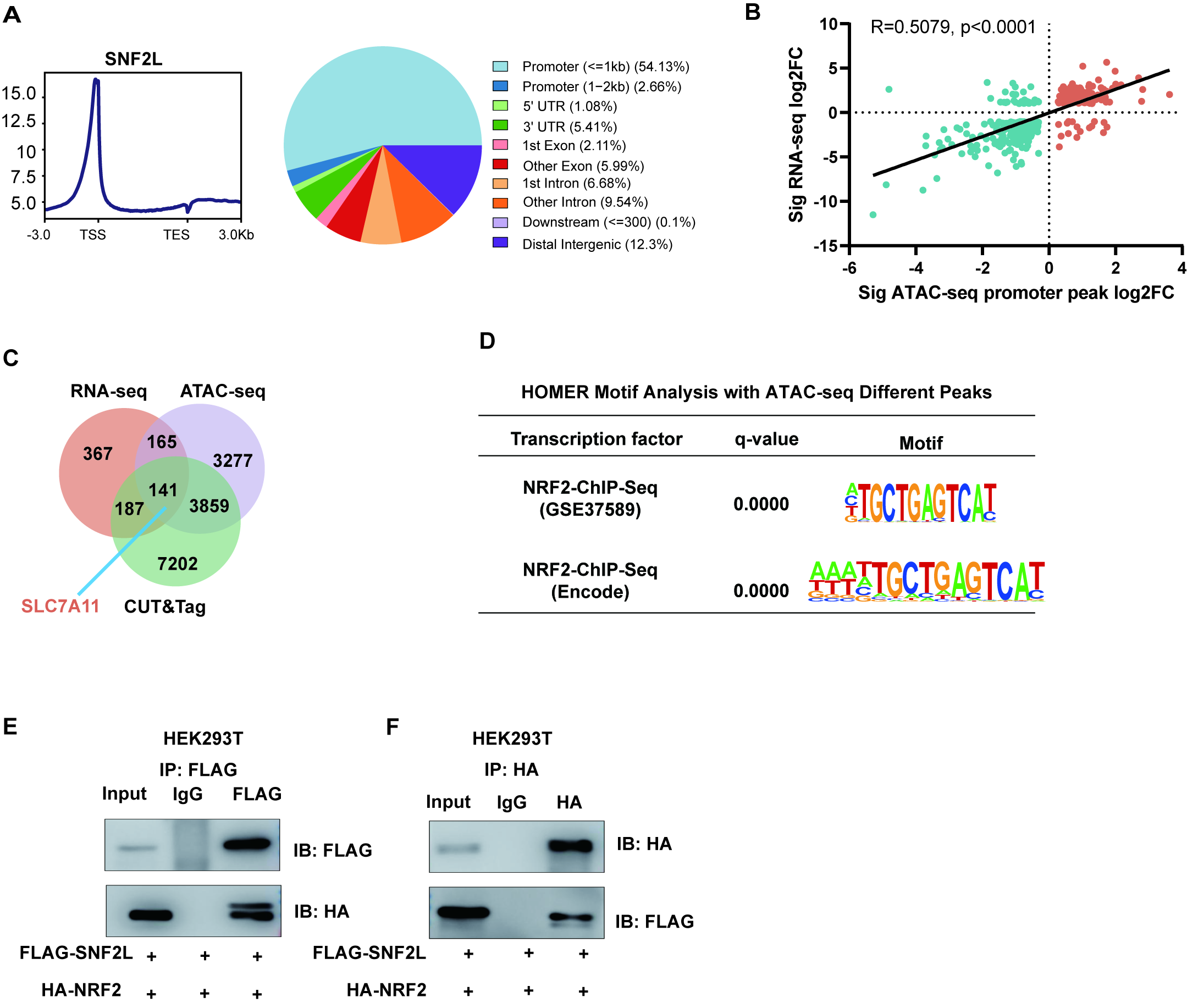

Supplement: Supplementary file 5 — FigS4. SNF2L increases SLC7A11 expression by regulating chromatin accessibility. [file 41419_2024_7221_MOESM5_ESM.tif]

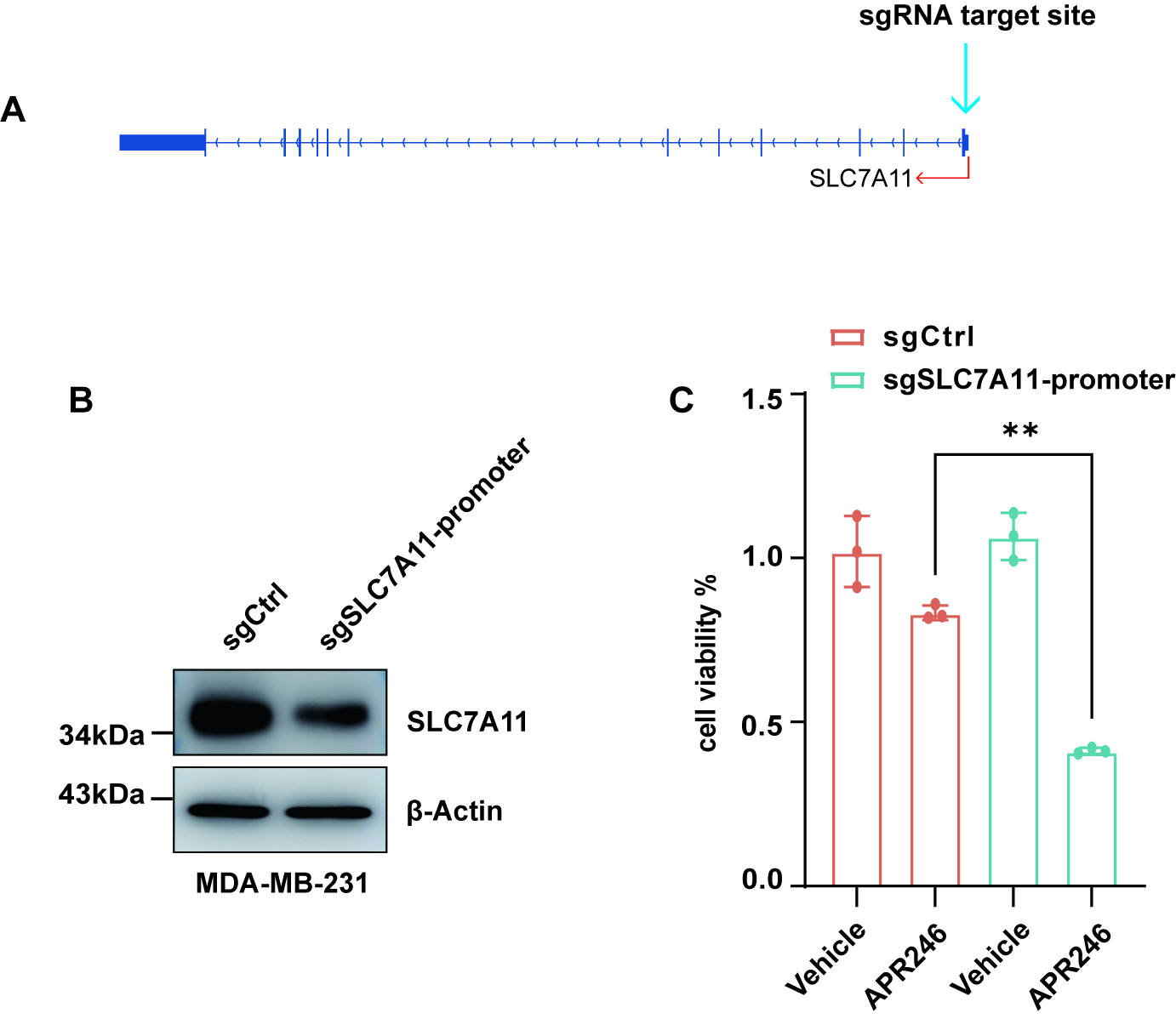

Supplement: Supplementary file 6 — FigS5. SNF2L enhances SLC7A11 expression by binding to its promoter. [file 41419_2024_7221_MOESM6_ESM.tif]
