# Supplementary material for: SNF2L maintains glutathione homeostasis by initiating SLC7A11 transcription through chromatin remodeling
Source: Cell Death Dis. 2024 Nov 12;15(11):820. doi: 10.1038/s41419-024-07221-4 (PMC11557580; doi:10.1038/s41419-024-07221-4)
Supplement: Supplementary file 7 — Supplementary Table [file 41419_2024_7221_MOESM7_ESM.pdf]

Supplementary Table S1. Metabolites identified by NMR-based metabolomics analysis

Sample	WT1	WT2	WT3	WT4	KO1	KO2	KO3	KO4	log2(FC)	-Log10(P value)
Glutathione	3.19	6.61	5.53	6.34	2.90	1.06	0.50	1.36	-1.90	2.27
Formic acid	3.73	14.92	5.96	11.23	3.02	3.79	3.57	2.85	-1.44	1.17
L-Glutamine	5.22	9.16	9.82	6.29	6.81	1.86	5.94	0.00	-1.06	1.04
L-Lactic acid	119.14	158.61	143.99	169.22	98.56	103.87	81.07	91.28	-0.66	2.40
Creatine	41.75	56.72	53.47	55.48	39.97	35.73	27.41	38.10	-0.55	2.02
myo-Inositol	8.04	12.10	10.28	10.11	7.77	6.71	6.05	8.19	-0.50	1.66
Phosphorylcholine	93.08	119.86	100.25	101.60	83.43	81.68	57.43	78.92	-0.46	1.84
L-Glutamate	74.74	89.75	81.22	89.27	69.48	67.46	49.60	67.26	-0.40	1.87
Citric acid	12.54	20.27	18.49	17.64	12.70	11.35	11.51	17.05	-0.39	0.99
L-Threonine	16.94	23.57	26.88	25.65	18.39	19.67	17.63	22.70	-0.25	0.72
L-Isoleucine	10.46	13.09	12.03	14.75	9.92	11.93	10.40	11.80	-0.19	0.75
Glycine	119.01	160.62	98.28	113.94	99.73	108.38	131.74	103.07	-0.15	0.35
Glycerophosphocholine	40.35	44.50	51.72	55.61	39.70	43.11	41.75	53.20	-0.11	0.34
L-Valine	13.15	14.85	21.78	22.98	16.39	17.50	15.83	20.02	-0.06	0.11
L-Alanine	20.32	19.78	23.34	24.75	18.88	20.81	20.94	23.94	-0.06	0.23
Glycerol	15.97	20.85	16.76	15.28	15.18	16.14	19.54	17.42	-0.01	0.03
Succinic acid	13.70	25.91	20.18	20.11	23.32	18.09	17.50	20.67	-0.01	0.01
L-Tyrosine	4.37	4.27	4.83	6.64	3.72	5.64	4.32	6.60	0.01	0.02
L-Leucine	28.52	28.95	39.75	39.81	26.10	35.75	35.74	41.72	0.02	0.04
UDP-sugars	-1.07	0.02	-3.17	-1.52	-2.76	-0.45	-1.20	-1.59	0.06	0.03
Taurine	9.68	12.38	11.89	11.65	11.83	13.10	9.28	13.89	0.08	0.21
Acetic acid	92.55	87.14	97.86	113.94	101.64	88.16	124.91	98.80	0.08	0.23
L-Phenylalanine	10.65	8.07	8.74	9.01	11.22	9.64	8.85	10.87	0.15	0.63
L-Histidine	0.00	1.75	2.28	0.97	2.08	1.72	0.83	1.00	0.17	0.10
Imidazole	2.41	1.47	4.33	1.53	2.46	1.18	3.55	4.02	0.20	0.15
D-Glucose	188.10	4.06	6.32	4.47	101.10	60.58	62.95	12.89	0.23	0.06
L-Aspartic acid	4.72	6.54	5.54	6.56	7.72	5.49	5.72	9.12	0.26	0.57
L-Lysine	3.20	5.57	8.34	9.50	6.96	8.31	8.18	9.93	0.33	0.50
Choline	14.61	12.79	18.71	16.35	20.14	24.85	24.83	24.31	0.59	2.46

Supplementary Table S2. Differentially expressed proteins identified using mass spectrometry-based quantitative proteomics
（fold change>1.5, p<0.05).

Protein name	Peptides	WT1	WT2	WT3	KO1	KO2	KO3	Log2FC	P-value
HOXD12	1	-3.75	-3.87	-3.92	-0.67	-0.67	-0.65	3.18	0.00
HTRA4	1	-4.66	-4.81	-4.73	-3.73	-2.25	-3.77	1.67	0.04
MAGEA1	5	-1.00	-1.03	-0.98	0.00	0.05	0.06	1.04	0.00
SERPINB7	4	-1.32	-1.36	-1.47	-0.39	-0.35	-0.35	1.02	0.00
PAGE1	8	1.13	1.15	1.14	2.10	2.11	2.10	0.97	0.00
FST	4	-1.30	-1.13	-1.12	-0.28	-0.32	-0.29	0.88	0.00
SERPINE2	7	0.18	0.06	0.10	0.89	0.95	0.93	0.81	0.00
MYH16	7	-2.59	-2.69	-2.61	-1.86	-1.85	-1.76	0.80	0.00
LRRC20	3	-4.00	-4.05	-3.89	-3.24	-3.08	-3.24	0.79	0.00
WRN	1	-4.55	-4.27	-4.40	-3.56	-3.70	-3.58	0.79	0.00
OTUD4	7	-0.88	-0.99	-0.88	-0.15	-0.13	-0.13	0.78	0.00
VCY1B	2	-4.42	-4.40	-4.42	-3.65	-3.64	-3.64	0.77	0.00
OSBPL10	3	-4.29	-4.16	-4.15	-3.46	-3.51	-3.35	0.76	0.00
SVIL	3	-4.69	-5.00	-4.83	-4.29	-4.11	-3.92	0.74	0.01
SBSN	10	1.18	1.21	1.21	1.90	1.92	1.94	0.72	0.00
VAT1L	2	-4.05	-4.17	-4.00	-3.47	-3.36	-3.36	0.68	0.00
TP53BP2	3	-3.37	-3.02	-3.43	-2.57	-2.86	-2.42	0.66	0.02
TTYH2	1	-1.74	-2.22	-1.56	-1.04	-1.10	-1.39	0.65	0.04
LIMA1	37	3.86	3.88	3.88	4.50	4.51	4.53	0.64	0.00
ZNF114	1	-8.32	-8.21	-8.32	-7.57	-7.70	-7.65	0.64	0.00
NRG1	2	-3.89	-3.74	-3.72	-3.13	-3.19	-3.15	0.63	0.00
CDCP1	3	-3.76	-3.68	-3.75	-3.15	-3.10	-3.12	0.60	0.00
H1-2	15	6.35	6.44	6.43	5.80	5.85	5.77	-0.60	0.00
TSN	8	2.43	2.44	2.41	1.83	1.83	1.82	-0.60	0.00
H1-4	15	6.36	6.44	6.43	5.80	5.85	5.77	-0.60	0.00
CEP97	2	-1.30	-1.28	-1.27	-1.94	-1.88	-1.85	-0.60	0.00
CCDC107	1	-1.81	-1.87	-1.86	-2.42	-2.43	-2.53	-0.61	0.00
MELTF	3	-2.47	-2.51	-2.66	-3.16	-3.22	-3.11	-0.62	0.00
TAF1C	1	-5.80	-6.14	-6.31	-6.67	-6.72	-6.73	-0.64	0.01
SLC7A11	2	-2.34	-2.26	-2.36	-2.94	-2.97	-3.03	-0.66	0.00
RPL22L1	4	1.89	1.87	1.85	1.23	1.18	1.18	-0.68	0.00
PCF11	2	-2.05	-2.03	-2.01	-2.70	-2.73	-2.72	-0.69	0.00
NQO1	11	3.92	3.90	3.90	3.17	3.16	3.20	-0.72	0.00
FTL	2	-1.88	-1.71	-2.02	-2.64	-2.78	-2.66	-0.83	0.00
ARAP3	1	-4.13	-3.36	-3.34	-4.24	-4.46	-4.51	-0.83	0.04
MAP3K7	4	1.08	0.68	0.44	0.02	-0.13	-0.14	-0.84	0.01

Supplementary Table S3. Differentially expressed genes identified by RNA-seq in both SNF2L-deficient and parental HT-1080 cells (fold change>2, p<0.05).

gene	WT1	WT2	WT3	KO1	KO2	KO3	log2(FC)
NFASC	5.46	5.79	14.20	421.47	427.27	428.38	5.65
UNC13A	0.91	0.00	0.00	7.51	14.84	11.58	5.22
COLEC12	0.00	0.97	0.00	9.65	8.48	9.92	4.86
RIOX1	0.00	0.97	0.00	7.51	8.48	10.75	4.79
CCDC198	0.00	0.97	0.00	7.51	8.48	8.27	4.65
PNMA6E	0.91	0.00	2.37	25.74	27.57	23.98	4.56
TSPY13P	0.00	1.93	2.37	33.25	33.93	30.60	4.51
LINC02474	0.91	0.00	0.00	6.43	7.42	6.62	4.49
CLEC2L	8.19	4.83	2.37	96.52	115.56	103.37	4.36
REC114	0.00	0.97	0.00	6.43	4.24	6.62	4.16
DKFZP434A062	0.91	0.00	0.00	6.43	5.30	4.13	4.13
ZNF570	0.91	6.76	0.00	37.54	36.05	42.18	3.92
ZNF253	0.00	1.93	0.00	12.87	8.48	7.44	3.90
RPS23P5	0.91	0.97	0.00	9.65	8.48	8.27	3.82
TBX20	0.00	0.97	1.18	8.58	10.60	10.75	3.80
ZNF582	0.91	0.00	2.37	15.01	12.72	15.71	3.73
CACNG7	7.28	5.79	4.73	53.62	88.00	91.79	3.71
ZNF559-ZNF177	0.91	0.97	0.00	7.51	7.42	9.10	3.68
KCTD9P6	10.00	8.69	5.92	91.16	128.29	78.56	3.60
HCN4	1.82	1.93	4.73	24.67	33.93	43.83	3.59
THBS1-IT1	0.91	1.93	1.18	19.30	13.78	14.89	3.58
QPRT	24.56	19.30	28.40	280.98	289.44	272.08	3.54
ACNATP	0.00	0.97	0.00	3.22	5.30	2.48	3.51
C1orf116	7.28	4.83	7.10	77.22	65.73	75.26	3.51
MYEOV	122.78	138.00	140.84	1453.17	1519.29	1404.21	3.45
LRRC26	0.00	1.93	2.37	16.09	13.78	16.54	3.43
PDE1C	74.58	76.24	56.81	760.37	669.00	728.57	3.38
MLXP1	10.91	4.83	4.73	66.49	71.03	69.47	3.34
KCNK13	0.91	4.83	0.00	15.01	19.08	22.33	3.30
ESR1	10.00	12.55	14.20	125.48	114.50	109.99	3.25
CCR3	0.91	2.90	0.00	13.94	10.60	11.58	3.25
ANKRD20A8P	15.46	13.51	8.28	126.55	95.42	127.35	3.23
DCC	70.94	34.74	82.84	587.70	542.83	607.00	3.20
COLEC10	3.64	1.93	0.00	21.45	16.96	12.40	3.19
LINC00923	0.91	0.97	0.00	7.51	5.30	4.13	3.18
MAGI1-IT1	0.91	0.97	0.00	6.43	6.36	4.13	3.18
ZNF93	0.00	0.97	2.37	9.65	12.72	7.44	3.16
SPIB	0.91	0.97	0.00	6.43	5.30	4.96	3.16
FMOD	0.00	0.00	1.18	3.22	3.18	4.13	3.15
TMEM130	0.91	0.00	2.37	9.65	9.54	9.92	3.15
TBC1D10C	0.91	0.00	0.00	3.22	3.18	1.65	3.15
PCDH11X	0.00	2.90	0.00	8.58	11.66	4.96	3.12
ZNF790	1.82	3.86	1.18	21.45	15.90	22.33	3.12
RPL3L	0.91	0.00	1.18	4.29	6.36	6.62	3.04
CSDC2	0.00	1.93	1.18	6.43	8.48	10.75	3.04
TRPM2	112.77	81.06	84.03	739.99	804.70	738.49	3.04
RBMY2FP	1.82	2.90	0.00	9.65	13.78	14.06	2.99
LINC02263	4.55	0.00	2.37	18.23	18.02	18.19	2.98
ATP1A3	3.64	2.90	3.55	28.96	22.26	28.12	2.98
FAM197Y9	0.00	0.00	1.18	2.14	2.12	4.96	2.96
ANKRD2	0.00	2.90	1.18	7.51	11.66	12.40	2.95
UCA1	525.67	519.18	609.50	4295.15	4186.78	4013.33	2.92
REN	0.00	0.97	1.18	4.29	5.30	6.62	2.92
SPTBN4	1.82	1.93	0.00	8.58	9.54	9.92	2.90
SERPINB7	483.83	522.07	519.55	3854.38	3900.53	3646.15	2.90
SNX18P14	0.91	0.97	7.10	21.45	22.26	23.16	2.90
CLDN9	0.91	0.00	0.00	3.22	1.06	2.48	2.89
INKA2-AS1	0.91	0.00	0.00	3.22	1.06	2.48	2.89
WRN	602.06	636.91	616.60	4605.09	4482.58	4631.91	2.89
GABRG2	13.64	12.55	17.75	89.01	101.78	133.14	2.88

PRND	0.91	1.93	1.18	6.43	12.72	9.10	2.81
PLEKHB1	0.91	3.86	0.00	8.58	14.84	9.92	2.81
BCAM	134.60	103.26	120.72	868.68	811.06	792.25	2.79
SULT1B1	23.65	14.48	9.47	117.97	112.38	95.93	2.78
NGFR	140.06	135.10	143.20	977.00	978.58	912.16	2.78
CYP4F32P	39.11	29.92	46.16	234.87	290.50	246.44	2.74
BGN	20.92	20.27	22.49	135.13	146.31	143.89	2.74
LINC01470	28.19	21.23	20.12	143.71	166.45	153.82	2.74
LINC00665	14.55	32.81	28.40	166.23	174.94	161.26	2.73
PLEKHA7	70.94	80.10	56.81	446.14	439.99	482.96	2.72
BMS1P4-AGAP5	0.91	0.00	0.00	3.22	1.06	1.65	2.71
LINC02256	0.91	0.00	0.00	2.14	2.12	1.65	2.70
PPP1R1B	1.82	1.93	1.18	7.51	13.78	10.75	2.70
MEGF11	9.09	4.83	4.73	40.75	41.35	38.04	2.69
VIRMA-DT	0.91	0.97	0.00	4.29	5.30	2.48	2.69
ZNF114	50.02	39.57	49.71	300.29	294.74	282.00	2.65
CNTNAP2	0.00	0.97	0.00	2.14	2.12	1.65	2.62
ST6GALNAC5	7.28	2.90	1.18	24.67	26.51	18.19	2.61
PLAC8	10.91	5.79	4.73	39.68	38.17	52.93	2.61
ZNF699	16.37	21.23	23.67	140.49	97.54	133.14	2.60
PRDM1	50.02	57.90	54.44	337.82	305.34	339.06	2.60
MIR9-1HG	0.91	2.90	1.18	7.51	12.72	9.92	2.60
CDH18	1.82	1.93	1.18	7.51	12.72	9.10	2.57
KCNN2	10.91	7.72	11.83	49.33	63.61	66.99	2.56
NBEAP1	1.82	1.93	3.55	15.01	9.54	18.19	2.55
LMCD1	30.01	22.20	22.49	140.49	141.01	154.65	2.55
SLC38A3	0.00	0.97	2.37	6.43	5.30	7.44	2.53
VGF	385.61	371.53	369.25	2015.13	2299.60	2167.51	2.52
CITED1	2.73	5.79	2.37	23.59	23.32	15.71	2.52
ZNF470	13.64	16.41	15.39	92.23	86.94	81.87	2.52
OLFM5P	0.91	0.97	0.00	3.22	4.24	3.31	2.52
NPR1	3.64	0.97	7.10	19.30	22.26	25.64	2.52
TSPEAR	1.82	1.93	3.55	15.01	15.90	10.75	2.51
GALNT12	8.19	4.83	7.10	34.32	42.41	38.04	2.51
MIR99AHG	11.82	9.65	11.83	58.98	62.55	67.81	2.51
PDGFB	72.76	63.69	52.07	336.75	339.27	357.26	2.45
DOCK3	75.49	98.43	57.99	391.44	406.06	458.15	2.44
PYCARD	0.91	0.00	0.00	2.14	1.06	1.65	2.42
VCX3A	3.64	0.00	3.55	12.87	10.60	14.89	2.42
TSSC2	335.59	343.55	312.44	1817.80	1734.51	1711.85	2.41
UNC5A	21.83	4.83	20.12	81.51	89.06	76.91	2.40
PEX11G	1.82	0.00	1.18	5.36	5.30	4.96	2.38
TCF7L1	52.75	41.50	50.89	229.50	250.21	272.90	2.37
KCND3	0.00	3.86	3.55	8.58	13.78	15.71	2.36
OR7E12P	31.83	29.92	31.95	181.24	132.53	157.95	2.33
MRGPRX4	9.09	16.41	8.28	71.85	47.71	50.45	2.33
EXTL1	2.73	0.00	1.18	9.65	4.24	5.79	2.33
SERPINB2	8.19	7.72	2.37	27.88	33.93	29.77	2.33
TUBB4A	49.11	38.60	69.83	243.45	254.45	291.10	2.32
ZNF542P	131.87	117.73	113.62	627.38	601.14	589.64	2.32
ARHGEF16	2.73	3.86	0.00	13.94	10.60	8.27	2.32
SMILR	0.00	3.86	3.55	11.80	11.66	13.23	2.31
FAM86GP	61.84	84.92	78.11	359.27	375.32	375.45	2.30
SYNPO2L	0.00	3.86	3.55	11.80	13.78	10.75	2.29
CKMT1A	8.19	10.62	9.47	48.26	45.59	44.66	2.29
TNFAIP6	3.64	1.93	0.00	8.58	9.54	9.10	2.29
ANGPT2	4.55	2.90	4.73	19.30	24.38	15.71	2.29
FBXO44	14.55	22.20	22.49	87.94	101.78	99.24	2.29
KIF26A	94.58	88.78	76.93	411.82	416.66	437.47	2.28
TCN1	9.09	11.58	13.02	46.12	51.95	64.50	2.27
FAM71F1	0.91	2.90	2.37	6.43	11.66	11.58	2.27
ZNF101P2	0.00	0.00	1.18	2.14	1.06	2.48	2.26
PHACTR3	0.00	0.00	1.18	1.07	2.12	2.48	2.26
SULF2	76.39	66.59	59.17	309.94	309.58	348.16	2.26
ADAMTS12	10.00	8.69	15.39	57.91	51.95	52.10	2.25
MYCT1	8.19	9.65	10.65	35.39	55.13	44.66	2.25

CCDC92B	0.00	1.93	0.00	3.22	4.24	1.65	2.24
SEMA3G	22.74	23.16	22.49	113.68	102.84	105.85	2.24
OTOGL	7.28	4.83	3.55	27.88	21.20	23.98	2.22
CDH4	200.99	178.53	168.06	813.99	855.59	876.60	2.22
BNIP3P4	0.91	4.83	1.18	11.80	9.54	10.75	2.21
CNN1	3.64	0.97	3.55	12.87	14.84	9.92	2.21
GIPC3	44.56	39.57	23.67	149.07	191.90	154.65	2.20
TMEM132B	22.74	8.69	8.28	64.35	60.43	57.06	2.20
RTL5	0.91	0.00	3.55	5.36	4.24	10.75	2.19
STKLD1	5.46	3.86	2.37	21.45	12.72	19.02	2.19
SHC4	34.56	23.16	29.59	117.97	118.74	159.61	2.18
KISS1	2.73	5.79	2.37	13.94	22.26	12.40	2.16
ASB9	44.56	62.73	57.99	241.30	261.87	234.86	2.16
LINC00654	0.91	0.00	0.00	2.14	1.06	0.83	2.15
MIA	0.91	0.00	0.00	2.14	1.06	0.83	2.15
NEFL	1141.37	1141.61	1061.59	4854.97	5001.03	4858.51	2.14
NFE2	1.82	0.00	2.37	5.36	6.36	6.62	2.13
TNFRSF11B	34.56	46.32	39.06	160.87	192.96	171.18	2.13
STPG3-AS1	0.00	0.00	2.37	2.14	3.18	4.96	2.12
SMCP	1.82	0.00	0.00	3.22	2.12	2.48	2.10
DLL4	4.55	7.72	2.37	16.09	28.63	18.19	2.10
SOWAHCP5	3.64	2.90	7.10	23.59	11.66	23.16	2.10
PEX5L	23.65	41.50	22.49	134.06	132.53	108.33	2.10
NME5	1.82	0.97	2.37	8.58	4.24	9.10	2.09
SAA1	17.28	20.27	20.12	81.51	78.46	81.87	2.07
LINC00958	3.64	7.72	1.18	19.30	15.90	17.37	2.07
PPIAP30	0.00	0.97	0.00	1.07	2.12	0.83	2.06
SLCO2A1	5.46	6.76	3.55	22.52	21.20	21.50	2.05
ACTBP7	31.83	21.23	31.95	109.39	128.29	114.12	2.05
PTPRR	56.39	66.59	53.26	233.79	260.81	231.55	2.04
PTGES3L	0.91	2.90	0.00	5.36	5.30	4.96	2.04
LINC02298	0.91	2.90	3.55	10.72	9.54	9.92	2.04
ADAM11	89.13	63.69	101.78	332.46	384.86	322.52	2.03
FRAS1	130.05	98.43	104.15	502.98	377.44	473.03	2.02
ZNF569	31.83	29.92	39.06	144.78	122.98	142.24	2.02
CMC4	6.37	3.86	1.18	13.94	15.90	16.54	2.02
RAB3A	24.56	18.34	23.67	90.09	89.06	90.97	2.02
MEGF10	1.82	1.93	1.18	5.36	6.36	8.27	2.02
CGN	88.22	89.75	84.03	374.28	335.03	351.47	2.02
HOATZ	2.73	0.97	1.18	9.65	4.24	5.79	2.01
PIANP	13.64	26.06	10.65	53.62	86.94	62.02	2.01
RSPO4	15.46	13.51	11.83	52.55	50.89	60.37	2.01
FAM43B	25.46	22.20	17.75	72.93	93.30	95.93	2.00
STXBP2	11.82	17.37	29.59	68.64	96.48	69.47	2.00
ITGB8	89.13	72.38	78.11	327.10	279.90	341.54	1.98
PLD4	3.64	0.97	1.18	9.65	7.42	5.79	1.98
PLCH1	7.28	5.79	9.47	31.10	33.93	23.16	1.97
B3GAT1	138.24	119.66	107.70	457.94	447.41	515.21	1.96
GRIK4	1.82	8.69	3.55	20.38	19.08	14.89	1.95
LINC02220	4.55	3.86	1.18	11.80	12.72	12.40	1.94
LINC01446	21.83	37.64	26.04	119.04	101.78	108.33	1.94
FGFR4	24.56	24.13	43.79	91.16	149.49	114.12	1.94
SEMA3D	148.24	125.45	100.60	496.54	448.47	488.74	1.94
HSD11B2	11.82	9.65	7.10	39.68	26.51	41.35	1.91
FIBCD1	40.02	32.81	36.69	107.24	162.21	138.11	1.90
AGBL5-IT1	0.91	2.90	1.18	4.29	8.48	5.79	1.90
ZNF433-AS1	6.37	2.90	2.37	17.16	12.72	13.23	1.89
GRIK2	12.73	5.79	10.65	39.68	28.63	38.04	1.87
LINC02575	22.74	37.64	40.24	109.39	126.17	129.84	1.86
RPL29P30	0.00	5.79	4.73	8.58	18.02	11.58	1.86
MYO1D	231.00	197.83	222.50	793.61	780.32	782.32	1.86
PAX6	27.28	30.88	29.59	105.10	90.12	119.09	1.84
DCT	160.07	136.07	146.75	589.85	466.49	525.96	1.84
GCK	5.46	4.83	10.65	21.45	24.38	28.94	1.84
EPHB1	173.71	136.07	150.30	544.80	538.59	559.87	1.84
PRSS3	55.48	53.08	57.99	189.82	216.28	186.90	1.83

PRKCG	2.73	4.83	1.18	10.72	9.54	10.75	1.83
TMEM59L	70.94	67.55	65.09	222.00	247.03	253.06	1.83
GALNT18	208.27	175.63	186.99	649.90	671.12	697.14	1.82
MFAP5	2.73	2.90	0.00	6.43	8.48	4.96	1.82
MIR3689D2	0.00	0.97	1.18	2.14	2.12	3.31	1.82
RPS20P22	4.55	3.86	1.18	10.72	10.60	12.40	1.81
ATP6V0D2	15.46	14.48	8.28	50.41	46.65	37.21	1.81
DUSP10	703.92	663.93	729.03	2396.92	2519.07	2435.46	1.81
FNDC5	6.37	10.62	8.28	25.74	29.69	33.08	1.81
INHBA	301.03	284.68	300.61	1062.80	979.64	1047.78	1.80
MAGEA1	540.22	520.14	466.30	1802.79	1803.42	1706.06	1.80
LCE1F	1.82	0.00	2.37	5.36	4.24	4.96	1.80
FST	2602.88	2489.74	2424.98	8734.02	8905.80	8517.06	1.80
FEZ1	50.93	72.38	36.69	185.53	178.12	189.38	1.79
LINC00622	7.28	10.62	8.28	31.10	25.45	33.91	1.79
CRLF1	59.11	58.87	71.01	212.34	209.92	228.25	1.78
NEDD9	24.56	19.30	34.32	78.29	99.66	88.49	1.77
FLRT3	78.21	96.50	75.74	297.07	280.96	273.73	1.77
LINC01605	8.19	10.62	23.67	57.91	36.05	50.45	1.77
HAPLN3	94.58	86.85	87.58	322.81	302.16	289.44	1.77
SKAP1	3.64	3.86	3.55	11.80	11.66	14.06	1.76
OTX1	10.00	4.83	3.55	19.30	21.20	21.50	1.75
MMP15	212.81	212.30	185.81	683.15	660.51	716.16	1.75
RTL3	60.93	36.67	47.34	182.32	147.37	158.78	1.75
MIR3659HG	5.46	1.93	4.73	17.16	12.72	10.75	1.75
BIK	7.28	14.48	9.47	39.68	30.75	33.91	1.74
LMTK3	5.46	3.86	4.73	15.01	16.96	14.89	1.74
P2RX2	0.91	0.00	5.92	7.51	5.30	9.92	1.74
TESPA1	6.37	7.72	4.73	19.30	23.32	19.85	1.73
ZNF738	60.93	44.39	50.89	165.16	181.30	169.53	1.72
ANO2	6.37	15.44	5.92	35.39	30.75	24.81	1.71
PPARGC1A	21.83	20.27	21.30	64.35	77.40	66.16	1.71
SH2D1B	38.20	42.46	28.40	129.77	116.62	109.99	1.71
SYCP2	10.91	11.58	9.47	42.90	30.75	30.60	1.71
SCN5A	115.50	105.19	108.88	369.99	335.03	369.66	1.71
LINC02236	26.37	23.16	26.04	72.93	84.82	88.49	1.70
THBS1	9361.08	9261.25	9573.28	31689.77	28938.53	31034.06	1.70
AACSP1	86.40	88.78	110.06	309.94	341.39	275.38	1.70
TRPC7	2.73	0.00	1.18	5.36	3.18	4.13	1.70
MYH16	1389.66	1416.64	1405.99	4521.44	4559.98	4557.49	1.70
CST7	9.09	7.72	8.28	25.74	22.26	33.08	1.69
GLB1L3	0.91	0.00	1.18	2.14	2.12	2.48	1.69
SEMA5A	85.49	97.47	87.58	303.50	266.11	301.85	1.69
NKILA	18.19	24.13	20.12	69.71	60.43	70.29	1.68
ZNF813	40.02	38.60	26.04	100.81	124.05	110.82	1.68
SH2D4B	0.91	1.93	1.18	4.29	5.30	3.31	1.68
CTB-178M22.2	2.73	0.97	1.18	5.36	5.30	4.96	1.68
KIF26B	27.28	11.58	11.83	63.27	44.53	54.58	1.68
LINC01500	0.00	0.00	1.18	1.07	1.06	1.65	1.68
SEPTIN14P8	1.82	0.97	0.00	3.22	3.18	2.48	1.67
GMFG	19.10	20.27	22.49	57.91	67.85	71.12	1.67
ZNF501	31.83	23.16	11.83	62.20	69.97	79.39	1.66
FBXL16	157.34	159.23	169.24	529.79	484.52	523.48	1.66
AADACP1	0.91	0.97	0.00	2.14	2.12	1.65	1.66
ZNF883	58.21	62.73	71.01	203.77	202.50	199.30	1.66
RGMA	5.46	1.93	3.55	11.80	12.72	9.92	1.65
SIGLEC14	17.28	18.34	11.83	46.12	58.31	44.66	1.65
SPRR2D	1.82	2.90	0.00	4.29	6.36	4.13	1.65
UBQLNL	0.00	0.97	1.18	1.07	3.18	2.48	1.65
PRR16	62.75	50.18	42.61	152.29	169.63	165.40	1.65
SCG2	110.95	148.61	104.15	349.62	405.00	380.41	1.64
PGAP4	150.06	149.58	149.12	462.23	467.55	465.59	1.64
LINC00163	3.64	3.86	2.37	10.72	10.60	9.10	1.62
COX7A1	4.55	0.97	2.37	8.58	7.42	8.27	1.62
ZNF559	31.83	35.71	28.40	87.94	98.60	107.51	1.62
ATCAY	0.00	2.90	0.00	2.14	4.24	2.48	1.61

CYP4F3	3.64	1.93	4.73	11.80	10.60	9.10	1.61
TMEM45A	20.92	23.16	24.85	83.65	71.03	54.58	1.60
TNS4	32.74	21.23	26.04	85.80	72.09	84.35	1.60
SFN	56.39	49.22	57.99	160.87	190.84	143.07	1.60
S1PR1	331.04	255.73	285.22	952.33	886.34	798.04	1.60
SLC27A3	3.64	3.86	3.55	11.80	11.66	9.92	1.60
TEX19	172.80	170.81	181.07	526.57	555.55	502.80	1.59
MN1	45.47	40.53	42.61	143.71	101.78	139.76	1.58
ATOH8	30.01	45.36	30.77	96.52	125.11	95.93	1.58
SAMD13	10.00	4.83	3.55	21.45	15.90	17.37	1.57
SLC11A1	0.91	3.86	4.73	8.58	10.60	9.10	1.57
PMEPA1	484.74	538.48	452.09	1433.86	1447.19	1478.64	1.56
VWCE	15.46	16.41	17.75	52.55	46.65	47.14	1.56
PAGE1	360.15	385.04	401.20	1076.74	1222.43	1075.90	1.56
ADGRL3	86.40	59.83	60.36	199.48	195.08	210.88	1.55
LRRC17	332.86	358.99	307.71	1001.67	987.06	932.01	1.55
MSI1	47.29	32.81	43.79	107.24	114.50	139.76	1.54
SERPINE2	3038.51	2936.54	2746.89	8376.89	8744.64	8324.38	1.54
ZNF680	23.65	35.71	24.85	75.07	91.18	79.39	1.54
TUB	210.09	197.83	132.55	513.70	520.57	538.36	1.54
CHRNA7	75.49	98.43	91.13	257.39	260.81	250.57	1.54
ZFP82	49.11	44.39	60.36	129.77	143.13	172.84	1.53
LINC02725	1.82	0.97	0.00	4.29	2.12	1.65	1.53
PBX1	16.37	15.44	11.83	48.26	31.81	46.31	1.53
B3GAT1-DT	1.82	0.97	0.00	3.22	3.18	1.65	1.53
EFR3B	114.59	82.03	110.06	275.62	324.43	285.31	1.53
BMF	9.09	10.62	7.10	19.30	32.87	24.81	1.52
SAA2	5.46	1.93	4.73	10.72	11.66	12.40	1.52
KANK3	3.64	1.93	4.73	10.72	6.36	12.40	1.52
LINC01910	49.11	54.04	26.04	105.10	136.77	127.35	1.52
ZNF71	52.75	42.46	50.89	134.06	161.15	121.57	1.51
IL1A	45.47	41.50	34.32	114.75	111.32	119.91	1.51
NXN	57.30	51.15	47.34	140.49	128.29	175.32	1.51
NHLRC4	2.73	1.93	2.37	5.36	6.36	8.27	1.51
CDC37P1	2.73	2.90	4.73	8.58	8.48	12.40	1.51
MIA2-AS1	2.73	2.90	4.73	10.72	9.54	9.10	1.50
HMGB1P41	0.91	0.97	1.18	3.22	2.12	3.31	1.50
SYTL5	6.37	9.65	20.12	35.39	24.38	42.18	1.50
RYR2	106.41	74.31	85.21	265.97	233.25	246.44	1.49
LINC02019	3.64	1.93	1.18	8.58	5.30	4.96	1.48
MPP2	55.48	54.04	54.44	134.06	161.15	161.26	1.48
ROR1	287.39	284.68	306.52	833.29	681.72	891.48	1.45
SERINC2	1606.11	1587.45	1574.05	4164.32	4530.29	4313.53	1.45
TNFSF12	26.37	20.27	35.50	66.49	75.28	81.87	1.44
MMP2	917.65	829.91	923.13	2402.28	2435.31	2432.97	1.44
SMCO3	0.91	0.97	0.00	2.14	2.12	0.83	1.44
PRSS55	0.91	0.97	0.00	2.14	2.12	0.83	1.44
RAB39B	5.46	11.58	9.47	24.67	25.45	21.50	1.43
GDF7	19.10	21.23	15.39	51.48	50.89	47.96	1.43
MYCL	33.65	43.43	33.14	83.65	129.35	84.35	1.43
RCOR2	1.82	9.65	14.20	20.38	22.26	26.46	1.43
SBSN	2094.49	1911.70	1961.05	5510.24	5558.70	4979.24	1.43
PLD5	54.57	63.69	53.26	153.36	155.85	151.34	1.43
ZKSCAN7-AS1	9.09	4.83	3.55	15.01	16.96	14.89	1.42
NAP1L3	35.47	34.74	29.59	89.01	101.78	76.91	1.42
NTNG2	245.55	252.83	237.88	641.32	701.86	630.99	1.42
C8G	2.73	6.76	3.55	12.87	13.78	8.27	1.42
PART1	4.55	1.93	1.18	7.51	6.36	6.62	1.42
SAMD3	13.64	13.51	13.02	38.61	38.17	30.60	1.42
KCNJ11	17.28	37.64	21.30	77.22	66.79	59.54	1.42
HOXC11	10.91	3.86	4.73	19.30	19.08	13.23	1.40
H2BC8	4.55	3.86	3.55	9.65	9.54	12.40	1.40
CCND2	50.02	41.50	40.24	131.91	116.62	99.24	1.40
FAM153B	0.00	2.90	1.18	2.14	5.30	3.31	1.40
GJD3	36.38	34.74	29.59	71.85	101.78	90.97	1.39
RBFOX3	6.37	5.79	11.83	22.52	18.02	22.33	1.39

LYPD1	403.80	379.25	396.47	974.86	1062.33	1033.72	1.38
TRPC7-AS1	0.00	0.00	3.55	2.14	2.12	4.96	1.38
ADAMTS7	145.51	102.29	117.17	268.11	336.09	343.20	1.38
DSG2-AS1	1.82	2.90	2.37	5.36	6.36	6.62	1.37
EMSLR	5.46	7.72	16.57	24.67	25.45	26.46	1.36
APBA1	36.38	34.74	28.40	81.51	81.64	92.62	1.36
NRXN3	153.70	109.05	144.39	375.36	331.85	334.93	1.36
SNRPN	27.28	22.20	24.85	65.42	63.61	61.20	1.36
JDP2-AS1	11.82	3.86	4.73	21.45	15.90	14.89	1.36
ZNF528	52.75	54.04	66.28	154.43	135.71	152.16	1.35
ELAVL2	20.92	21.23	31.95	53.62	66.79	68.64	1.35
CREG2	62.75	78.17	74.56	176.95	202.50	167.05	1.34
BTBD11	562.96	549.09	516.00	1370.59	1357.07	1400.08	1.34
NOX4	174.62	178.53	183.44	439.70	456.95	454.84	1.33
UTS2R	8.19	10.62	9.47	20.38	27.57	23.16	1.33
ZNF585A	16.37	23.16	17.75	46.12	42.41	55.41	1.33
TMSB15B	18.19	19.30	14.20	41.83	46.65	41.35	1.33
STRA6	10.00	2.90	4.73	15.01	12.72	16.54	1.33
CDA	158.25	172.74	147.94	368.92	449.53	383.72	1.33
FUCA1	91.86	82.99	63.91	212.34	208.86	176.97	1.33
TARS1-DT	23.65	18.34	30.77	61.13	68.91	52.10	1.32
KRT8P14	321.04	377.32	381.09	945.90	789.86	965.91	1.32
LINGO1	257.38	213.27	246.17	550.17	606.44	635.95	1.32
VAT1L	195.53	202.65	176.34	464.37	501.48	470.55	1.32
ZNF792	18.19	18.34	17.75	49.33	41.35	44.66	1.32
COL1A1	100.95	82.99	113.62	215.56	261.87	261.33	1.31
BCO2	14.55	19.30	7.10	27.88	38.17	35.56	1.31
SEMA3A	318.31	342.58	297.06	839.73	741.09	789.77	1.31
GLT8D2	59.11	65.62	67.46	160.87	149.49	164.57	1.31
ABCA13	54.57	45.36	40.24	110.46	114.50	120.74	1.30
SGCG	2.73	1.93	2.37	5.36	5.30	6.62	1.30
PRG4	0.91	1.93	0.00	2.14	3.18	1.65	1.30
SOD2-OT1	5.46	0.97	1.18	7.51	5.30	5.79	1.29
ZNF853	48.20	51.15	40.24	113.68	126.17	100.89	1.29
CLIP4	62.75	70.45	85.21	198.40	177.06	157.13	1.29
EDA	58.21	55.97	37.87	126.55	110.26	132.32	1.28
MARCHF3	210.09	213.27	198.83	496.54	504.66	505.28	1.28
MAOA	460.19	510.49	472.21	1162.53	1229.85	1098.23	1.27
SERPINF1	50.02	35.71	42.61	130.84	82.70	95.93	1.27
ARID3B	445.64	405.31	439.08	1045.64	1043.25	1021.32	1.27
AMOT	275.57	257.66	239.07	676.71	581.00	603.70	1.27
KCNK2	67.30	60.80	34.32	124.40	129.35	137.28	1.27
ADAMTS15	34.56	47.29	35.50	97.59	92.24	92.62	1.27
MOB3B	217.36	208.44	260.37	578.05	530.11	542.50	1.27
LRRC4B	7.28	9.65	5.92	20.38	18.02	16.54	1.27
KLHDC8B	60.02	51.15	55.62	140.49	122.98	136.45	1.26
WNT10B	86.40	65.62	81.66	175.88	200.38	182.76	1.26
MITF	285.57	320.39	320.73	743.21	731.55	736.84	1.25
TMEM121B	10.91	19.30	18.94	25.74	47.71	43.83	1.25
ZNF10	12.73	22.20	16.57	43.97	46.65	32.25	1.25
KCNH1-IT1	15.46	12.55	13.02	33.25	38.17	26.46	1.25
TRIM54	4.55	3.86	3.55	9.65	10.60	8.27	1.25
FAM27B	10.91	7.72	3.55	18.23	21.20	13.23	1.25
TMEFF1	15.46	11.58	11.83	34.32	29.69	28.12	1.24
TSPEAR-AS2	112.77	126.42	106.51	243.45	295.80	277.04	1.24
RIPOR3	2.73	1.93	1.18	5.36	4.24	4.13	1.23
WNT7B	1044.97	1054.76	1069.88	2510.60	2519.07	2419.74	1.23
SNHG18	30.01	38.60	24.85	79.36	69.97	70.29	1.23
CORO1A	148.24	144.75	137.29	300.29	367.89	341.54	1.23
MAST1	9.09	15.44	18.94	28.96	38.17	34.73	1.23
ZNF641	67.30	66.59	80.48	161.94	177.06	162.91	1.23
ATP2A3	58.21	48.25	56.81	129.77	119.80	132.32	1.23
LINC01704	11.82	8.69	18.94	26.81	33.93	31.43	1.22
BEX1	218.27	210.37	236.70	476.17	586.30	490.40	1.22
FGFBP3	72.76	82.03	85.21	173.74	181.30	205.09	1.22
VAMP8	2.73	3.86	4.73	8.58	9.54	8.27	1.22

PDP1	2176.34	2151.98	1912.53	5056.59	4464.56	5022.25	1.22
DACT1	10.91	8.69	3.55	19.30	18.02	16.54	1.22
LINC00869	28.19	18.34	18.94	47.19	53.01	52.10	1.22
PPL	12.73	7.72	14.20	24.67	24.38	31.43	1.22
LRRC20	428.36	400.48	440.26	936.25	1043.25	955.16	1.21
ARHGAP5-AS1	29.10	28.95	23.67	54.69	60.43	73.60	1.21
MYOM3	139.15	126.42	113.62	282.05	291.56	301.85	1.21
DIPK1B	81.85	87.82	80.48	159.79	226.89	189.38	1.20
MAPK8IP2	24.56	32.81	21.30	55.77	63.61	61.20	1.20
PCSK5	117.32	130.28	126.63	294.92	275.66	286.13	1.19
COL16A1	70.94	72.38	79.29	191.97	174.94	141.41	1.19
TMEM98	336.50	278.89	334.93	747.50	725.19	696.32	1.19
UCN2	190.08	201.69	201.19	407.53	491.94	453.18	1.19
CNTN3	178.25	143.79	137.29	369.99	341.39	334.10	1.19
ZNF493	14.55	17.37	15.39	28.96	41.35	37.21	1.18
LINC02582	16.37	12.55	13.02	39.68	23.32	32.25	1.18
RASSF2	47.29	39.57	26.04	87.94	84.82	83.52	1.18
NECTIN4	141.88	140.89	115.98	284.20	305.34	315.08	1.18
DRD2	59.11	60.80	60.36	123.33	143.13	142.24	1.18
PTCH1	136.42	111.94	124.27	245.59	308.52	288.62	1.18
SDS	0.91	0.00	3.55	3.22	1.06	5.79	1.17
RNVU1-24	15.46	13.51	10.65	27.88	30.75	30.60	1.17
NKAIN1	28.19	22.20	23.67	50.41	54.07	62.02	1.17
FREM1	4.55	1.93	3.55	7.51	7.42	7.44	1.16
DLX2-DT	0.00	3.86	4.73	3.22	8.48	7.44	1.16
CDCP1	1869.85	1727.38	1871.10	4173.97	3839.03	4166.32	1.16
LRATD1	289.21	261.52	293.51	649.90	612.80	616.10	1.15
TGFBI	924.01	882.02	932.59	1946.49	2127.85	2011.21	1.15
GABRB1	7.28	0.00	7.10	11.80	8.48	11.58	1.15
COLGALT2	180.07	203.62	169.24	415.04	408.18	400.26	1.15
MTUS1	278.29	305.91	326.64	638.11	655.21	719.47	1.14
POTEH	20.92	23.16	33.14	52.55	50.89	66.99	1.14
FBXL22	7.28	9.65	5.92	16.09	16.96	17.37	1.14
TGFB2	553.86	546.20	518.37	1310.53	1065.52	1195.81	1.14
POU3F1	5.46	4.83	5.92	13.94	8.48	13.23	1.14
MISP3	15.46	5.79	21.30	30.03	29.69	33.91	1.14
DAAM1	273.75	315.56	263.92	686.37	546.01	642.56	1.14
LINC01151	5.46	12.55	14.20	16.09	22.26	32.25	1.13
CYP1A1	34.56	19.30	27.22	52.55	61.49	63.68	1.13
BRSK2	67.30	44.39	57.99	116.90	101.78	152.99	1.13
KRT7	107.32	121.59	111.25	211.27	278.84	254.71	1.13
DOC2A	14.55	10.62	22.49	36.46	33.93	33.91	1.13
PTPRN2	14.55	11.58	9.47	26.81	25.45	25.64	1.13
TMEM231P1	4.55	3.86	1.18	8.58	7.42	4.96	1.13
SLC18A2	20.01	27.99	18.94	47.19	47.71	51.27	1.13
MID1	1096.81	1070.20	1028.46	2366.89	2232.81	2374.26	1.13
GSTT2	8.19	7.72	8.28	17.16	19.08	16.54	1.13
GASK1B	12.73	13.51	8.28	31.10	23.32	20.67	1.12
IRF5	43.65	27.02	40.24	71.85	81.64	87.66	1.12
MMP9	95.49	82.03	108.88	188.75	200.38	232.38	1.12
SUB1P1	6.37	3.86	3.55	13.94	8.48	7.44	1.12
ZNF410	8.19	13.51	4.73	20.38	21.20	15.71	1.12
NKD1	18.19	27.02	28.40	49.33	57.25	52.93	1.12
SMC1B	45.47	45.36	53.26	111.53	94.36	105.85	1.11
ZNF138	60.02	74.31	65.09	140.49	168.57	122.39	1.11
PADI3	8.19	11.58	17.75	19.30	28.63	33.08	1.11
RBPMS	335.59	336.79	358.60	729.26	773.96	721.95	1.11
GULP1	401.07	368.64	370.43	834.36	776.08	847.65	1.11
ATP8B5P	0.91	1.93	1.18	2.14	3.18	3.31	1.10
DAAM2	71.85	82.99	71.01	145.85	159.03	179.45	1.10
HSD3BP5	15.46	20.27	18.94	40.75	45.59	30.60	1.10
COL8A1	141.88	121.59	142.02	282.05	306.40	278.69	1.10
AOAH	9.09	18.34	7.10	24.67	27.57	21.50	1.09
OTULINL	140.97	106.15	123.08	225.21	275.66	289.44	1.09
LINC00472	257.38	315.56	239.07	598.43	569.33	565.65	1.09
STXBP6	166.43	182.39	204.74	403.24	406.06	369.66	1.09

SLIT2	95.49	71.41	72.19	193.04	134.65	180.28	1.09
RPL13AP20	136.42	161.16	152.67	357.13	310.64	287.79	1.09
HHAT	121.87	169.84	156.22	308.87	329.73	310.12	1.08
MIR1281	2.73	2.90	4.73	6.43	6.36	9.10	1.08
BMP2	57.30	49.22	34.32	99.74	96.48	100.89	1.08
GPRC5B	249.19	231.60	231.96	495.47	476.04	530.92	1.08
HYAL1	25.46	20.27	21.30	43.97	55.13	42.18	1.08
BMERB1	19.10	20.27	18.94	46.12	31.81	44.66	1.07
C1QL4	114.59	85.89	104.15	242.37	181.30	216.67	1.07
EPB41L4B	371.06	358.02	345.58	771.09	720.95	755.86	1.06
FRMD5	845.80	897.46	860.40	1886.44	1763.14	1796.20	1.06
KCNH1	30.01	31.85	33.14	58.98	63.61	76.08	1.06
PARD6G	234.64	242.22	222.50	498.69	481.34	482.13	1.06
FAM13C	23.65	22.20	11.83	47.19	40.29	33.08	1.06
S100A4	68.21	79.13	66.28	161.94	156.91	127.35	1.06
RTN4RL1	266.47	224.85	235.52	486.89	522.69	505.28	1.06
CLU	1292.34	1292.16	1313.68	2621.06	2841.37	2653.78	1.06
PDZD7	48.20	32.81	53.26	87.94	98.60	92.62	1.06
LINC01694	271.93	227.74	207.11	516.92	451.65	497.01	1.05
HBG2	73.67	80.10	56.81	154.43	134.65	147.20	1.05
NR4A1	60.93	48.25	50.89	110.46	102.84	118.26	1.05
B3GALT5	289.21	244.15	282.86	572.69	532.23	582.19	1.05
SYNE3	140.97	135.10	117.17	291.71	267.17	253.06	1.05
PKP2	141.88	135.10	151.49	307.79	294.74	282.00	1.05
CLMAT3	18.19	10.62	18.94	32.17	23.32	43.00	1.04
AK5	61.84	92.64	68.64	166.23	167.51	125.70	1.04
HSD17B2	52.75	61.76	44.97	109.39	96.48	121.57	1.04
MIR4653	4.55	3.86	4.73	7.51	9.54	9.92	1.04
LIMA1	3435.03	3433.52	3514.98	7186.47	7076.93	7016.10	1.04
PPM1E	111.86	138.00	101.78	242.37	210.98	267.11	1.03
OR51K1P	34.56	37.64	33.14	70.78	75.28	69.47	1.03
LINC02783	2.73	6.76	3.55	8.58	10.60	7.44	1.03
PAX8-AS1	771.22	763.33	680.51	1533.60	1558.51	1407.52	1.02
GREB1	195.53	200.72	173.97	352.84	371.07	430.03	1.02
VAV3	30.92	41.50	39.06	72.93	77.40	75.26	1.02
ITGB1BP2	10.91	7.72	5.92	20.38	15.90	13.23	1.01
PDZRN3	105.50	84.92	100.60	193.04	201.44	191.03	1.01
RPL12P14	58.21	39.57	47.34	91.16	100.72	100.06	1.01
TSPAN18	206.45	226.78	241.43	451.50	436.81	465.59	1.00
AMIGO1	167.34	163.09	146.75	288.49	343.51	325.00	1.00
PRAG1	1516.07	1426.29	1422.56	2905.26	2813.81	3031.71	1.00
AOC2	70.03	82.99	65.09	143.71	161.15	132.32	1.00
ST3GAL5	68.21	104.22	75.74	148.00	157.97	191.03	1.00
PPM1H	65.48	70.45	69.83	122.26	144.19	145.55	1.00
MIR29B2CHG	25.46	24.13	23.67	17.16	9.54	9.92	-1.00
SLFN11	691.19	551.99	615.42	328.17	301.10	299.37	-1.00
RPL39P5	1.82	3.86	2.37	1.07	2.12	0.83	-1.00
KATNAL1	544.77	569.36	485.23	311.01	238.55	248.09	-1.00
RCBTB2	106.41	96.50	104.15	62.20	47.71	43.00	-1.01
STEAP2	260.11	234.50	250.90	136.20	98.60	136.45	-1.01
DACH1	35.47	29.92	28.40	20.38	10.60	15.71	-1.01
EIF5A2	1086.81	1095.29	1024.91	538.37	526.93	530.92	-1.01
LINC00452	68.21	81.06	60.36	40.75	32.87	30.60	-1.01
LINC00862	4.55	2.90	1.18	3.22	1.06	0.00	-1.01
LRRIQ3	14.55	19.30	18.94	7.51	9.54	9.10	-1.01
GALNT15	25.46	23.16	36.69	12.87	12.72	16.54	-1.02
MT-TV	44.56	54.04	69.83	24.67	28.63	29.77	-1.02
ALDH1L1	1024.96	1008.44	1013.07	456.86	556.61	488.74	-1.02
HULC	13.64	23.16	20.12	4.29	13.78	9.92	-1.02
IQCH-AS1	33.65	27.99	35.50	13.94	18.02	15.71	-1.03
KDM4D	69.12	73.34	74.56	37.54	30.75	38.04	-1.03
PLXNA1	15160.71	13907.80	14555.79	7241.17	6553.18	7431.24	-1.04
ECSCR	55.48	48.25	68.64	23.59	25.45	34.73	-1.04
XXYLT1	699.38	669.72	641.45	307.79	342.45	326.66	-1.04
ADGRF4	58.21	49.22	53.26	23.59	25.45	28.94	-1.04
CRYL1	162.79	169.84	163.32	66.49	82.70	90.97	-1.05

SPSB3	12.73	9.65	8.28	8.58	2.12	4.13	-1.05
MAP1A	154.61	138.00	140.84	80.43	64.67	63.68	-1.05
MIR3667HG	218.27	216.16	182.26	96.52	106.02	93.45	-1.06
LMLN	204.63	207.48	173.97	96.52	83.76	100.89	-1.06
DHX58	61.84	52.11	57.99	26.81	23.32	32.25	-1.06
CFH	7.28	4.83	4.73	4.29	2.12	1.65	-1.06
BRPF3-AS1	5.46	7.72	7.10	3.22	3.18	3.31	-1.06
FAM230C	95.49	95.54	117.17	51.48	45.59	50.45	-1.06
ZDHHC23	405.62	380.22	407.12	200.55	168.57	201.78	-1.06
LINC01711	111.86	152.47	142.02	60.06	65.73	68.64	-1.06
FAHD2B	24.56	20.27	34.32	8.58	12.72	16.54	-1.06
CPQ	77.30	72.38	87.58	34.32	36.05	43.00	-1.07
ZNF185	1132.28	1127.14	1118.40	575.90	504.66	532.57	-1.07
ZNF620	32.74	29.92	27.22	9.65	18.02	14.89	-1.08
RHBDL2	24.56	16.41	18.94	9.65	9.54	9.10	-1.08
N4BP2L1	12.73	15.44	17.75	5.36	6.36	9.92	-1.09
PNRC1	277.39	255.73	253.27	107.24	137.83	124.87	-1.09
HTATSF1P2	100.04	85.89	99.41	48.26	42.41	43.00	-1.09
ALG1L6P	80.94	66.59	92.31	41.83	26.51	43.83	-1.10
SAMD5	147.33	172.74	145.57	83.65	69.97	63.68	-1.10
VEGFA	2491.01	2421.22	2355.15	1090.68	1110.04	1190.02	-1.10
LINC02742	85.49	88.78	97.05	45.04	46.65	34.73	-1.10
RRS1-DT	10.00	7.72	8.28	7.51	2.12	2.48	-1.10
NR3C2	102.77	87.82	115.98	51.48	34.99	56.23	-1.10
SERPINB1	562.96	565.50	547.96	250.95	254.45	272.90	-1.11
CFAP58	41.84	53.08	43.79	18.23	25.45	20.67	-1.11
PAPPA2	442.91	477.68	489.97	202.69	217.34	232.38	-1.11
NAALADL2	27.28	26.06	27.22	16.09	9.54	11.58	-1.11
INTS6-AS1	12.73	16.41	10.65	5.36	9.54	3.31	-1.13
DAPK1	55.48	55.01	53.26	26.81	30.75	17.37	-1.13
ZSCAN5C	0.91	0.97	3.55	0.00	0.00	2.48	-1.13
BMT2	144.60	137.03	130.18	61.13	65.73	61.20	-1.13
ADTRP	150.97	129.31	151.49	64.35	68.91	63.68	-1.13
TATDN2P2	160.97	148.61	149.12	80.43	49.83	78.56	-1.14
NFIB	1344.18	1207.23	1217.82	585.56	565.09	563.17	-1.14
FAM111A-DT	38.20	45.36	40.24	16.09	24.38	15.71	-1.14
GAS6	4525.48	4272.12	4341.06	2025.85	2060.00	1867.32	-1.14
DLX6-AS1	30.92	27.99	28.40	11.80	16.96	10.75	-1.14
EHHADH	86.40	92.64	78.11	35.39	40.29	40.52	-1.15
SPTLC3	106.41	142.82	126.63	41.83	71.03	56.23	-1.15
CFHR3	3.64	1.93	4.73	2.14	0.00	2.48	-1.16
SNORD19	4.55	2.90	4.73	2.14	0.00	3.31	-1.16
CSF1R	80.03	87.82	74.56	25.74	48.77	33.91	-1.16
APOL1	235.55	253.80	249.72	125.48	100.72	103.37	-1.17
SLC7A11	1788.91	1859.58	1811.93	855.81	720.95	855.10	-1.17
IL12A	122.78	135.10	107.70	57.91	47.71	57.06	-1.17
POU6F1	64.57	62.73	55.62	28.96	23.32	28.94	-1.17
ARHGAP30	501.11	447.77	462.75	219.85	208.86	193.51	-1.18
LINC01363	2.73	0.97	1.18	2.14	0.00	0.00	-1.19
APOL3	48.20	47.29	50.89	19.30	23.32	21.50	-1.19
POU2F2	197.35	157.30	162.14	65.42	80.58	80.22	-1.19
MPZL3	116.41	89.75	97.05	48.26	48.77	35.56	-1.19
ZNF701	17.28	23.16	18.94	7.51	8.48	9.92	-1.20
ABCC3	222.82	218.09	244.98	86.87	103.90	108.33	-1.20
BAHCC1	89.13	102.29	111.25	45.04	41.35	45.48	-1.20
GABRE	152.79	209.41	222.50	89.01	69.97	95.10	-1.20
EVA1A-AS	2.73	1.93	3.55	1.07	0.00	2.48	-1.21
ARAP2	276.48	286.61	314.81	139.42	114.50	125.70	-1.21
P2RY1	526.58	538.48	544.41	257.39	214.16	221.63	-1.22
SLC12A8	66.39	57.90	54.44	25.74	18.02	33.08	-1.22
RNF5P1	17.28	14.48	8.28	9.65	4.24	3.31	-1.22
KIT	18.19	9.65	13.02	8.58	3.18	5.79	-1.22
ST7-AS1	10.91	13.51	9.47	4.29	5.30	4.96	-1.22
ZNF391	16.37	15.44	20.12	7.51	3.18	11.58	-1.22
LINC01615	16.37	20.27	21.30	5.36	9.54	9.92	-1.22
LNCSRLR	10.00	6.76	7.10	6.43	2.12	1.65	-1.22

C3orf70	147.33	138.96	158.59	58.98	65.73	64.50	-1.23
RNF152P1	0.91	2.90	1.18	0.00	2.12	0.00	-1.23
KRCC1	165.52	209.41	191.73	78.29	88.00	74.43	-1.24
SENP8	37.29	50.18	50.89	16.09	24.38	18.19	-1.24
GPR160	89.13	77.20	72.19	27.88	31.81	41.35	-1.24
FAM167A	546.59	509.53	587.01	249.88	212.04	231.55	-1.24
C1GALT1C1L	17.28	14.48	13.02	5.36	8.48	4.96	-1.25
DMBT1	96.40	97.47	74.56	38.61	31.81	42.18	-1.25
NPFFR2	262.83	241.25	227.23	97.59	93.30	115.78	-1.25
CAB39L	95.49	137.03	102.96	45.04	54.07	41.35	-1.26
RAB32	676.64	605.06	594.11	260.60	277.78	243.96	-1.26
ARMCX4	283.75	323.28	289.96	112.61	116.62	144.72	-1.26
CGB8	50.02	50.18	46.16	17.16	21.20	22.33	-1.27
CCNG2	335.59	364.78	284.04	127.62	119.80	160.43	-1.27
ATRNL1	14.55	14.48	13.02	7.51	3.18	6.62	-1.28
FPR2	4.55	2.90	2.37	2.14	1.06	0.83	-1.28
SLC7A11-AS1	11.82	5.79	10.65	5.36	2.12	4.13	-1.28
TMEM47	65.48	91.68	69.83	37.54	38.17	17.37	-1.29
SH3GL3	40.93	53.08	40.24	18.23	20.14	16.54	-1.29
CCDC68	5.46	2.90	3.55	2.14	1.06	1.65	-1.29
RP1L1	36.38	36.67	33.14	20.38	6.36	16.54	-1.29
PCDH18	32.74	33.78	39.06	15.01	13.78	14.06	-1.30
INPP5D	111.86	108.08	94.68	37.54	40.29	49.62	-1.30
BEND6	84.58	85.89	95.86	33.25	40.29	33.91	-1.31
RNF133	1.82	5.79	2.37	0.00	3.18	0.83	-1.32
APLF	38.20	40.53	33.14	16.09	18.02	10.75	-1.32
GNAL	58.21	61.76	66.28	26.81	25.45	22.33	-1.32
FAM229B	30.01	36.67	36.69	13.94	11.66	15.71	-1.32
NEURL1B	58.21	48.25	55.62	16.09	28.63	19.02	-1.35
LINC01358	105.50	107.12	118.35	33.25	55.13	41.35	-1.35
RPL11P3	1.82	0.97	3.55	0.00	0.00	2.48	-1.35
NUPR1	21.83	25.09	28.40	8.58	11.66	9.10	-1.36
ADGRG2	10.00	18.34	15.39	3.22	6.36	7.44	-1.36
RETREG1	20.92	37.64	26.04	9.65	14.84	8.27	-1.37
PLEKHG4	143.69	121.59	129.00	51.48	53.01	47.96	-1.37
RNF224	5.46	8.69	8.28	0.00	5.30	3.31	-1.38
LINC00601	82.76	87.82	115.98	26.81	49.83	33.08	-1.39
MCF2L2	69.12	82.03	62.73	25.74	28.63	27.29	-1.39
MELTF	2864.80	2835.22	2967.02	1085.32	1083.54	1127.17	-1.39
L1CAM	20.92	32.81	23.67	9.65	10.60	9.10	-1.40
IQSEC1	95.49	103.26	102.96	42.90	33.93	37.21	-1.40
PCDHA6	2.73	0.97	3.55	1.07	0.00	1.65	-1.41
SEPSECS-AS1	16.37	16.41	20.12	7.51	7.42	4.96	-1.41
SYTL3	260.11	194.93	235.52	83.65	81.64	94.28	-1.41
DGKG	285.57	251.87	273.39	97.59	114.50	91.79	-1.42
SLC22A23	503.84	465.14	487.60	154.43	191.90	198.48	-1.42
OR9A3P	3.64	0.97	1.18	2.14	0.00	0.00	-1.43
BMP2K-DT	3.64	0.97	1.18	2.14	0.00	0.00	-1.43
ESR2	6.37	5.79	10.65	3.22	1.06	4.13	-1.44
RNU6-623P	1.82	0.97	2.37	1.07	0.00	0.83	-1.44
GAPDHP70	1.82	0.97	2.37	1.07	0.00	0.83	-1.44
FAM241B	111.86	129.31	124.27	41.83	37.11	55.41	-1.44
RTN1	502.02	470.93	487.60	170.52	178.12	187.72	-1.45
PTGES	92.76	117.73	87.58	34.32	36.05	38.87	-1.45
LINC02100	1.82	3.86	2.37	0.00	2.12	0.83	-1.45
TFDP1P2	13.64	12.55	7.10	5.36	4.24	2.48	-1.46
TNFSF15	21.83	17.37	21.30	7.51	4.24	9.92	-1.48
SH2D4A	306.49	304.94	313.63	116.90	130.41	83.52	-1.48
HERC2P9	31.83	20.27	16.57	13.94	6.36	4.13	-1.49
NPIPB9	3.64	4.83	3.55	1.07	3.18	0.00	-1.50
SLC16A10	133.69	116.77	86.40	34.32	46.65	38.04	-1.50
RPAP3-DT	1.82	4.83	2.37	0.00	3.18	0.00	-1.50
CCN3	97.31	89.75	80.48	32.17	36.05	25.64	-1.51
MAT1A	13.64	12.55	10.65	6.43	3.18	3.31	-1.51
WDR31	16.37	8.69	16.57	6.43	3.18	4.96	-1.51
CPLX1	4.55	5.79	8.28	1.07	2.12	3.31	-1.52

OASL	26.37	28.95	20.12	10.72	10.60	4.96	-1.52
RBPMS2	94.58	87.82	94.68	18.23	47.71	30.60	-1.52
MT-TE	10.00	12.55	13.02	0.00	7.42	4.96	-1.52
EGLN3	9.09	13.51	8.28	2.14	5.30	3.31	-1.52
RNF150	39.11	46.32	41.42	8.58	14.84	20.67	-1.52
C1R	34.56	32.81	36.69	9.65	18.02	8.27	-1.53
APCDD1L	107.32	109.05	102.96	30.03	37.11	43.00	-1.54
DNAH11	128.23	138.96	179.89	60.06	40.29	53.75	-1.54
COX5BP6	8.19	9.65	10.65	0.00	3.18	6.62	-1.54
CCDC191	34.56	35.71	27.22	11.80	11.66	9.92	-1.55
LINC01182	78.21	90.71	74.56	24.67	29.69	28.94	-1.55
CXCL3	85.49	65.62	94.68	24.67	27.57	31.43	-1.55
LAMC3	39.11	36.67	34.32	6.43	16.96	14.06	-1.56
LINC02051	1.82	1.93	3.55	0.00	0.00	2.48	-1.56
RPS17	12.73	17.37	17.75	5.36	4.24	6.62	-1.56
HFE	187.35	172.74	157.40	50.41	58.31	66.16	-1.57
HSPB1	3.64	2.90	4.73	2.14	0.00	1.65	-1.57
Y_RNA	5.46	4.83	5.92	2.14	0.00	3.31	-1.57
PTENP1	7.28	11.58	8.28	2.14	5.30	1.65	-1.58
CAMK1D	100.04	87.82	98.23	32.17	34.99	27.29	-1.60
INHBB	362.87	353.20	324.28	112.61	118.74	110.82	-1.60
SHISA2	3.64	3.86	4.73	1.07	2.12	0.83	-1.61
EGF	41.84	39.57	30.77	16.09	11.66	9.10	-1.61
PTGIR	50.93	42.46	57.99	16.09	12.72	20.67	-1.61
PECAM1	26.37	25.09	27.22	8.58	6.36	10.75	-1.61
AGGF1P1	20.01	21.23	16.57	3.22	10.60	4.96	-1.62
HEY2	22.74	15.44	21.30	3.22	5.30	10.75	-1.63
ICA1	40.93	39.57	43.79	11.80	15.90	12.40	-1.63
SYBU	55.48	59.83	44.97	9.65	21.20	20.67	-1.64
ZFP90	69.12	61.76	62.73	16.09	20.14	25.64	-1.65
RPS6KA2	336.50	337.76	315.99	107.24	108.14	100.89	-1.65
TFCP2L1	18.19	14.48	13.02	9.65	3.18	1.65	-1.66
GJB2	326.50	335.83	334.93	105.10	117.68	91.79	-1.66
TIRAP	50.93	42.46	44.97	13.94	11.66	17.37	-1.69
GBP2	151.88	114.84	131.37	45.04	28.63	49.62	-1.69
GOLGA2P11	30.01	34.74	37.87	11.80	11.66	8.27	-1.69
MIR4477A	1.82	1.93	2.37	0.00	1.06	0.83	-1.70
FAM83A	5.46	4.83	4.73	2.14	0.00	2.48	-1.70
TAGLN3	146.42	153.44	171.61	46.12	50.89	47.96	-1.70
SMIM14	82.76	110.01	80.48	23.59	31.81	28.12	-1.71
LARGE1	64.57	64.66	75.74	25.74	12.72	23.98	-1.71
TAGLN	152.79	138.00	150.30	39.68	54.07	40.52	-1.72
C1S	89.13	88.78	84.03	19.30	24.38	35.56	-1.72
SEPTIN3	8.19	9.65	10.65	0.00	5.30	3.31	-1.73
SNORA5A	3.64	1.93	3.55	1.07	0.00	1.65	-1.74
ACSS1	8.19	9.65	11.83	1.07	5.30	2.48	-1.74
PHEX	64.57	64.66	68.64	23.59	22.26	12.40	-1.76
DDAH2	31.83	33.78	35.50	10.72	5.30	13.23	-1.79
MIR3677	1.82	4.83	3.55	0.00	2.12	0.83	-1.79
HSPB8	231.91	233.53	201.19	68.64	59.37	63.68	-1.80
ULK2	36.38	60.80	52.07	6.43	11.66	24.81	-1.80
FES	100.04	116.77	121.90	27.88	33.93	34.73	-1.81
ZNF77	9.09	18.34	14.20	3.22	5.30	3.31	-1.82
NUDT9P1	4.55	2.90	5.92	1.07	1.06	1.65	-1.82
IGF2BP2-AS1	6.37	4.83	5.92	1.07	2.12	1.65	-1.82
FUT9	101.86	117.73	99.41	41.83	26.51	21.50	-1.83
PANX2	280.11	282.75	220.13	68.64	89.06	56.23	-1.87
KCNRG	6.37	6.76	4.73	2.14	1.06	1.65	-1.88
EFL1P2	3.64	2.90	3.55	1.07	0.00	1.65	-1.89
PRXL2A	1.82	0.97	1.18	1.07	0.00	0.00	-1.89
TUBA4B	1.82	0.97	1.18	1.07	0.00	0.00	-1.89
ACOT11	45.47	70.45	43.79	11.80	18.02	13.23	-1.89
ADAM12	74.58	79.13	81.66	22.52	22.26	18.19	-1.90
RANBP20P	4.55	5.79	4.73	1.07	2.12	0.83	-1.91
LINC02641	1.82	2.90	2.37	0.00	1.06	0.83	-1.91
SLC44A3	7.28	5.79	8.28	2.14	1.06	2.48	-1.91

CMPK2	56.39	75.27	52.07	17.16	19.08	12.40	-1.92
LYRM4-AS1	56.39	41.50	63.91	10.72	18.02	14.06	-1.92
HECW1	50.93	50.18	44.97	12.87	11.66	14.06	-1.92
PRUNE2	16.37	20.27	18.94	5.36	8.48	0.83	-1.92
ABI3BP	80.94	77.20	59.17	27.88	7.42	21.50	-1.94
GABRR2	8.19	14.48	15.39	2.14	5.30	2.48	-1.94
CXCL8	1575.19	1579.73	1442.68	382.86	390.16	415.14	-1.95
CDRT1	13.64	11.58	8.28	2.14	3.18	3.31	-1.96
SGMS2	542.04	524.97	551.51	136.20	137.83	129.84	-2.00
SIRPB1	55.48	57.90	62.73	18.23	14.84	10.75	-2.01
DLG3	206.45	214.23	224.86	47.19	61.49	50.45	-2.02
PRDM6	7.28	9.65	10.65	3.22	1.06	2.48	-2.03
NIPAL2	84.58	82.03	72.19	20.38	22.26	14.89	-2.05
MRPS18CP7	3.64	1.93	2.37	1.07	0.00	0.83	-2.06
IL6	17.28	21.23	15.39	3.22	6.36	3.31	-2.06
ANXA10	35.47	27.99	22.49	8.58	5.30	6.62	-2.07
RPL7AP50	7.28	4.83	5.92	2.14	2.12	0.00	-2.08
ANAPC1P1	2.73	2.90	2.37	0.00	1.06	0.83	-2.08
RACGAP1P1	2.73	2.90	2.37	0.00	1.06	0.83	-2.08
CADM4	16.37	22.20	17.75	4.29	3.18	5.79	-2.09
ARHGAP6	27.28	22.20	28.40	4.29	10.60	3.31	-2.10
CNIH2	40.93	44.39	42.61	9.65	12.72	7.44	-2.10
IGFL3	7.28	5.79	7.10	1.07	1.06	2.48	-2.13
DNAJC12	1.82	2.90	3.55	0.00	1.06	0.83	-2.13
CECR2	15.46	14.48	17.75	2.14	2.12	6.62	-2.13
KCNF1	21.83	14.48	16.57	4.29	4.24	3.31	-2.16
MT-TT	6.37	5.79	2.37	2.14	1.06	0.00	-2.18
SPA17	83.67	87.82	101.78	15.01	21.20	23.98	-2.18
RPSAP17	2.73	0.97	1.18	1.07	0.00	0.00	-2.19
PRRG4	14.55	19.30	18.94	3.22	4.24	4.13	-2.19
OLFML3	40.02	32.81	36.69	11.80	2.12	9.92	-2.20
LINC01293	8.19	10.62	9.47	2.14	3.18	0.83	-2.20
TRAF1	111.86	98.43	111.25	15.01	27.57	27.29	-2.20
KRT79	66.39	56.94	44.97	7.51	22.26	6.62	-2.21
PDLIM3	133.69	122.56	98.23	33.25	19.08	23.16	-2.23
MGAT4A	65.48	74.31	84.03	21.45	9.54	16.54	-2.24
MACROH2A2	22.74	31.85	30.77	2.14	7.42	8.27	-2.26
CASP1	38.20	40.53	30.77	6.43	10.60	5.79	-2.26
TMEM71	78.21	94.57	74.56	16.09	21.20	14.06	-2.27
ZSCAN31	82.76	66.59	88.76	16.09	15.90	17.37	-2.27
FAM184A	18.19	32.81	24.85	5.36	5.30	4.96	-2.28
SMOC1	78.21	81.06	88.76	17.16	22.26	10.75	-2.31
VANGL2	35.47	38.60	49.71	4.29	7.42	13.23	-2.31
NCLP1	8.19	7.72	7.10	0.00	2.12	2.48	-2.32
STOX1	8.19	6.76	9.47	1.07	2.12	1.65	-2.33
PCDHGB1	16.37	22.20	18.94	7.51	2.12	1.65	-2.35
GCNT2P1	0.91	0.97	2.37	0.00	0.00	0.83	-2.36
RNU6-611P	0.91	0.97	2.37	0.00	0.00	0.83	-2.36
BTBD10P2	0.91	0.97	2.37	0.00	0.00	0.83	-2.36
PLEKHH2	44.56	42.46	49.71	9.65	9.54	7.44	-2.36
WDFY4	10.00	6.76	8.28	2.14	1.06	1.65	-2.37
MAGED1	555.68	521.11	497.07	100.81	83.76	116.60	-2.39
CCDC190	77.30	78.17	71.01	9.65	15.90	17.37	-2.40
SETP20	2.73	3.86	3.55	1.07	0.00	0.83	-2.42
FZD9	4.55	6.76	8.28	1.07	0.00	2.48	-2.46
CEACAM19	31.83	32.81	49.71	6.43	8.48	5.79	-2.47
MPZL2	20.01	27.02	14.20	1.07	7.42	2.48	-2.48
MCF2L	32.74	26.06	30.77	6.43	2.12	7.44	-2.49
NMU	4.55	3.86	3.55	1.07	1.06	0.00	-2.49
FLT1	26.37	17.37	20.12	3.22	3.18	4.96	-2.49
NFKBIL1	1.82	2.90	4.73	0.00	0.00	1.65	-2.51
LINC02009	138.24	144.75	163.32	24.67	28.63	24.81	-2.51
VGLL3	41.84	34.74	33.14	3.22	11.66	4.13	-2.53
SLC37A1	110.95	124.49	137.29	12.87	24.38	27.29	-2.53
CCDC110	10.91	12.55	13.02	3.22	2.12	0.83	-2.56
SLC1A1	12.73	16.41	13.02	1.07	4.24	1.65	-2.60

DCDC2C	30.01	26.06	35.50	5.36	6.36	3.31	-2.61
IGFBP2	179.16	141.86	140.84	30.03	18.02	27.29	-2.62
TMEM217	11.82	7.72	15.39	2.14	1.06	2.48	-2.62
PGBD5	100.95	96.50	85.21	19.30	10.60	15.71	-2.63
ENTPD3	1.82	0.97	2.37	0.00	0.00	0.83	-2.64
NDUFA5P12	1.82	0.97	2.37	0.00	0.00	0.83	-2.64
PCDH12	56.39	31.85	54.44	8.58	4.24	9.92	-2.65
PSTPIP2	61.84	60.80	33.14	11.80	6.36	6.62	-2.65
SLC9A9	15.46	20.27	20.12	3.22	3.18	2.48	-2.65
KCNH4	0.91	1.93	2.37	0.00	0.00	0.83	-2.65
APOBEC3G	63.66	57.90	48.52	9.65	7.42	9.92	-2.66
KCNAB2	617.52	517.25	589.38	95.45	97.54	78.56	-2.67
SQOR	40.93	38.60	39.06	4.29	8.48	5.79	-2.68
CILP	130.96	142.82	166.87	20.38	24.38	23.98	-2.68
RUNDC3B	18.19	31.85	31.95	2.14	6.36	4.13	-2.70
PLCL1	8.19	9.65	8.28	1.07	2.12	0.83	-2.70
SLC44A5	31.83	35.71	44.97	3.22	3.18	10.75	-2.71
DKK1	24.56	35.71	43.79	2.14	5.30	8.27	-2.73
RBM47	11.82	20.27	22.49	1.07	2.12	4.96	-2.74
IGSF9	13.64	11.58	10.65	4.29	1.06	0.00	-2.75
CA9	8.19	5.79	5.92	1.07	1.06	0.83	-2.75
DCHS1	13.64	9.65	15.39	2.14	1.06	2.48	-2.77
SPNS3	39.11	29.92	39.06	5.36	2.12	8.27	-2.78
CHRDL1	9.09	11.58	7.10	2.14	1.06	0.83	-2.78
JAKMIP2	3.64	4.83	4.73	0.00	1.06	0.83	-2.81
SLAIN1	117.32	121.59	131.37	20.38	15.90	16.54	-2.81
BDKRB1	15.46	11.58	23.67	2.14	0.00	4.96	-2.84
DRAIC	2.73	4.83	5.92	0.00	1.06	0.83	-2.84
FOXP2	35.47	37.64	59.17	9.65	2.12	6.62	-2.85
ZNF141	15.46	22.20	15.39	2.14	1.06	4.13	-2.85
PPFIBP2	61.84	50.18	54.44	7.51	6.36	9.10	-2.86
THEMIS2	9.09	11.58	15.39	2.14	1.06	1.65	-2.89
TMPRSS15	28.19	37.64	39.06	5.36	2.12	6.62	-2.90
ZNF555	6.37	8.69	7.10	2.14	0.00	0.83	-2.90
AKR1C3	60.93	73.34	53.26	7.51	8.48	9.10	-2.90
ARHGAP28	11.82	13.51	11.83	1.07	2.12	1.65	-2.94
SDC2	179.16	175.63	176.34	13.94	25.45	29.77	-2.94
PLPPR2	242.83	234.50	198.83	23.59	34.99	28.12	-2.96
PDE3A	30.92	24.13	26.04	3.22	2.12	4.96	-2.98
ST6GAL1	344.69	332.93	343.21	39.68	41.35	47.96	-2.98
RPL35P5	4.55	3.86	4.73	0.00	0.00	1.65	-2.99
CXXC4	21.83	20.27	29.59	2.14	1.06	5.79	-2.99
ADAMTS19-AS1	10.00	6.76	7.10	1.07	1.06	0.83	-3.01
SLC46A1	16.37	21.23	13.02	0.00	2.12	4.13	-3.02
FAM167B	9.09	15.44	15.39	2.14	1.06	1.65	-3.04
SMARCA1	1142.28	1240.04	1223.73	148.00	146.31	142.24	-3.05
RUNDC3A-AS1	10.00	17.37	13.02	1.07	2.12	1.65	-3.06
EBF3	1.82	2.90	2.37	0.00	0.00	0.83	-3.10
NIPAL4	1.82	2.90	2.37	0.00	0.00	0.83	-3.10
SLC4A4	13.64	19.30	9.47	1.07	2.12	1.65	-3.13
CDK5R2	10.00	8.69	7.10	0.00	2.12	0.83	-3.13
ICAM1	17.28	20.27	16.57	1.07	0.00	4.96	-3.16
ILDR2	99.13	108.08	69.83	4.29	11.66	14.89	-3.17
TLR3	69.12	64.66	63.91	5.36	7.42	9.10	-3.18
ADCYAP1R1	20.92	19.30	21.30	3.22	0.00	3.31	-3.24
OTOF	1.82	4.83	3.55	0.00	1.06	0.00	-3.27
COL22A1	59.11	73.34	72.19	9.65	7.42	4.13	-3.27
ADAM21P1	2.73	2.90	2.37	0.00	0.00	0.83	-3.27
PDE7B	43.65	27.02	39.06	5.36	4.24	1.65	-3.29
BCL2A1	103.68	96.50	112.43	9.65	9.54	12.40	-3.31
JUP	63.66	57.90	61.54	9.65	6.36	2.48	-3.31
SYNM	119.14	100.36	123.08	10.72	10.60	13.23	-3.31
CYP4F2	55.48	55.97	56.81	8.58	4.24	4.13	-3.31
NTSR1	5.46	8.69	7.10	1.07	1.06	0.00	-3.32
DAW1	1.82	2.90	3.55	0.00	0.00	0.83	-3.32
CLDN2	5.46	2.90	2.37	1.07	0.00	0.00	-3.32

ASTN1	12.73	8.69	16.57	0.00	2.12	1.65	-3.33
SOX21	20.92	19.30	15.39	0.00	2.12	3.31	-3.36
LINC01287	230.09	226.78	221.31	32.17	14.84	19.02	-3.36
AJAP1	1228.68	1194.69	1230.83	114.75	110.26	122.39	-3.39
DEPDC7	6.37	7.72	5.92	1.07	0.00	0.83	-3.40
ADGRB3	10.00	9.65	11.83	1.07	1.06	0.83	-3.41
TP73	8.19	6.76	8.28	2.14	0.00	0.00	-3.44
CSMD2	50.02	43.43	65.09	4.29	5.30	4.96	-3.45
SOX21-AS1	27.28	19.30	15.39	0.00	3.18	2.48	-3.45
CNDP1	96.40	84.92	104.15	6.43	10.60	8.27	-3.50
SESN3	184.62	216.16	189.36	15.01	19.08	18.19	-3.50
GPM6A	23.65	19.30	18.94	1.07	1.06	3.31	-3.51
GUCY1A1	25.46	24.13	13.02	1.07	4.24	0.00	-3.56
LINC00632	4.55	4.83	3.55	1.07	0.00	0.00	-3.59
BDKRB2	13.64	14.48	11.83	0.00	0.00	3.31	-3.59
NUP210	255.56	238.36	242.62	23.59	15.90	20.67	-3.61
ROR2	75.49	84.92	95.86	6.43	6.36	7.44	-3.66
ZNF285	17.28	20.27	16.57	0.00	4.24	0.00	-3.67
NPY1R	23.65	20.27	11.83	3.22	1.06	0.00	-3.70
TPO	21.83	20.27	27.22	0.00	5.30	0.00	-3.71
CCL2	65.48	52.11	53.26	3.22	6.36	3.31	-3.73
AFP	9.09	7.72	8.28	0.00	1.06	0.83	-3.73
ATF7IP2	19.10	13.51	17.75	0.00	2.12	1.65	-3.74
GSPT2	267.38	233.53	237.88	17.16	14.84	23.16	-3.74
COCH	10.91	9.65	9.47	2.14	0.00	0.00	-3.81
NMRAL2P	78.21	78.17	99.41	8.58	4.24	4.96	-3.85
DUOXA1	3.64	4.83	3.55	0.00	0.00	0.83	-3.86
ADGRE1	21.83	9.65	16.57	3.22	0.00	0.00	-3.90
CACNG4	26.37	11.58	27.22	4.29	0.00	0.00	-3.93
RBM24	3.64	6.76	5.92	0.00	1.06	0.00	-3.94
ITGA11	22.74	30.88	17.75	2.14	0.00	2.48	-3.95
PFKFB2	81.85	80.10	69.83	6.43	1.06	7.44	-3.96
PATJ	19.10	13.51	18.94	0.00	0.00	3.31	-3.96
LONRF2	182.80	176.60	182.26	19.30	8.48	6.62	-3.98
ACTL8	4464.54	4166.94	4168.27	275.62	269.29	247.27	-4.01
NDNF	19.10	34.74	26.04	2.14	1.06	1.65	-4.04
PRICKLE3	98.22	97.47	97.05	5.36	5.30	6.62	-4.08
GMPR	12.73	7.72	8.28	0.00	0.00	1.65	-4.12
PRSS12	14.55	11.58	17.75	0.00	0.00	2.48	-4.14
SEMA6D	9.09	6.76	3.55	1.07	0.00	0.00	-4.18
DDX43	41.84	36.67	33.14	0.00	5.30	0.83	-4.19
HOXA7	16.37	7.72	10.65	1.07	0.00	0.83	-4.19
TSPAN15	23.65	27.02	23.67	2.14	1.06	0.83	-4.20
XK	23.65	23.16	29.59	1.07	2.12	0.83	-4.25
ANGPTL2	13.64	9.65	8.28	0.00	0.00	1.65	-4.25
CCL20	6.37	9.65	4.73	0.00	1.06	0.00	-4.29
CHRNA9	48.20	39.57	49.71	3.22	2.12	1.65	-4.30
SNHG31	5.46	5.79	5.92	0.00	0.00	0.83	-4.38
LUARIS	4.55	10.62	7.10	0.00	1.06	0.00	-4.39
CSPG4	653.90	621.47	595.30	32.17	19.08	35.56	-4.43
PIEZO2	53.66	56.94	49.71	0.00	1.06	5.79	-4.55
ARSI	11.82	17.37	15.39	0.00	1.06	0.83	-4.56
CYB561	82.76	97.47	105.33	4.29	4.24	3.31	-4.59
DLX3	7.28	6.76	5.92	0.00	0.00	0.83	-4.59
LZTS1	43.65	29.92	33.14	3.22	1.06	0.00	-4.64
REEP2	18.19	16.41	13.02	1.07	0.00	0.83	-4.65
GJA3	19.10	15.44	14.20	1.07	0.00	0.83	-4.68
LRRK2	20.92	24.13	18.94	0.00	0.00	2.48	-4.69
TINAGL1	484.74	421.71	459.20	11.80	27.57	13.23	-4.70
S1PR3	7.28	7.72	7.10	0.00	0.00	0.83	-4.74
BIRC3	24.56	39.57	33.14	0.00	1.06	2.48	-4.78
IFT27	6.37	9.65	7.10	0.00	0.00	0.83	-4.80
FBLN1	90.04	81.06	75.74	6.43	0.00	1.65	-4.93
NDN	7.28	12.55	13.02	1.07	0.00	0.00	-4.94
PDZD4	9.09	10.62	13.02	0.00	1.06	0.00	-4.95
TMEM200B	45.47	47.29	59.17	0.00	3.18	1.65	-4.97

PTPRZ1	11.82	11.58	13.02	0.00	1.06	0.00	-5.10
ICAM5	24.56	25.09	16.57	0.00	1.06	0.83	-5.13
NCALD	132.78	99.40	121.90	5.36	1.06	3.31	-5.19
HOXD13	21.83	20.27	18.94	0.00	0.00	1.65	-5.21
XYLT1	63.66	57.90	54.44	0.00	1.06	3.31	-5.33
SALL4	10.91	8.69	15.39	0.00	0.00	0.83	-5.40
MIR4500HG	15.46	15.44	15.39	0.00	1.06	0.00	-5.45
NME4	541.13	491.19	461.56	9.65	8.48	12.40	-5.61
PTPRN	50.02	29.92	34.32	0.00	2.12	0.00	-5.75
SH3BP1	80.03	82.03	49.71	1.07	1.06	1.65	-5.81
P3H2	18.19	17.37	24.85	0.00	1.06	0.00	-5.83
IGFBP3	21.83	26.06	18.94	1.07	0.00	0.00	-5.96
GAGE1	245.55	222.92	242.62	2.14	7.42	1.65	-5.99
ACTN2	22.74	17.37	28.40	1.07	0.00	0.00	-6.00
ADAMTS2	346.50	340.65	307.71	2.14	4.24	9.10	-6.01
NRROS	47.29	50.18	43.79	2.14	0.00	0.00	-6.04
NEBL	24.56	19.30	11.83	0.00	0.00	0.83	-6.07
PDPN	80.94	56.94	63.91	0.00	1.06	1.65	-6.22
AMPH	91.86	99.40	111.25	0.00	1.06	1.65	-6.80
MANEAL	139.15	131.24	114.80	0.00	2.12	0.83	-7.03
GPC4	227.37	235.46	226.05	0.00	3.18	0.83	-7.43
CDH11	1086.81	1045.11	1062.78	2.14	4.24	4.96	-8.14
PHF21B	129.14	112.91	113.62	0.00	0.00	0.83	-8.75
PXDN	1078.62	995.90	1008.34	0.00	1.06	0.00	-11.51

Supplementary Table S4. Oligonucleotides used in this work.

**shRNA**	
shSNF2L1-1F	CCGGGCTGTAACACTCTGATTTCATCTCGAGATGAAATCAGAGTGTTACAGCTTTTTG
shSNF2L1-1R	AATTCAAAAAGCTGTAACACTCTGATTTCATCTCGAGATGAAATCAGAGTGTTACAGC
shSNF2L1-2F	CCGGGTGTATTCATGGTACTCTAAGCTCGAGCTTAGAGTACCATGAATACACTTTTTG
shSNF2L1-2R	AATTCAAAAAGTGTATTCATGGTACTCTAAGCTCGAGCTTAGAGTACCATGAATACAC
shSLC7A11-F	CCGGTATTCTATGAGTCGCACAATTCTCGAGAATTGTGCGACTCATAGAATATTTTTG
shSLC7A11-R	AATTCAAAAATATTCTATGAGTCGCACAATTCTCGAGAATTGTGCGACTCATAGAATA
**sgRNA**	
Hu-SNF2L1-F	caccgCGCCACTATCGTGGTCATAG
Hu-SNF2L1-R	aaacCTATGACCACGATAGTGGCGc
**RT-qPCR primers**	
Hu-SNF2L1-F	TCTCCAAAGGAGGTTACCTGC
Hu-SNF2L1-R	AGACTCCCCTCAGTAAAGTGAC
