# Supplementary material for: SNF2L maintains glutathione homeostasis by initiating SLC7A11 transcription through chromatin remodeling
Source: Cell Death Dis. 2024 Nov 12;15(11):820. doi: 10.1038/s41419-024-07221-4 (PMC11557580; doi:10.1038/s41419-024-07221-4)

Figure 1A

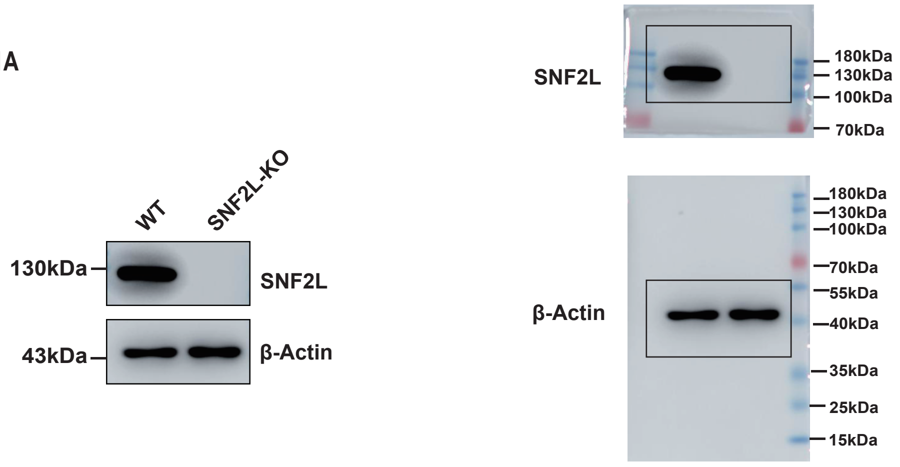

Figure 2H

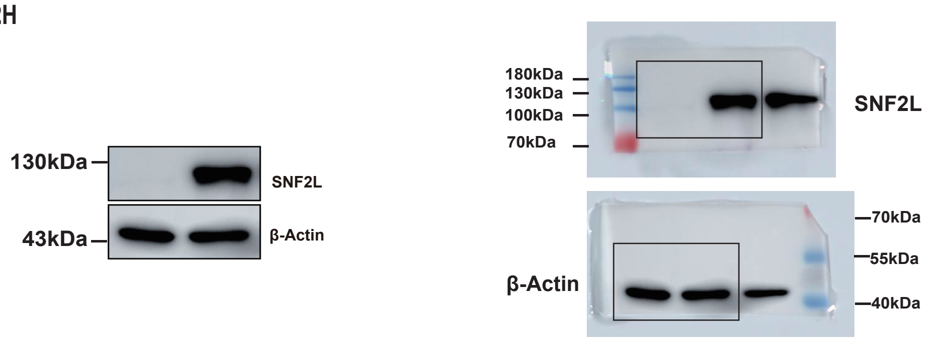

Figure 3A

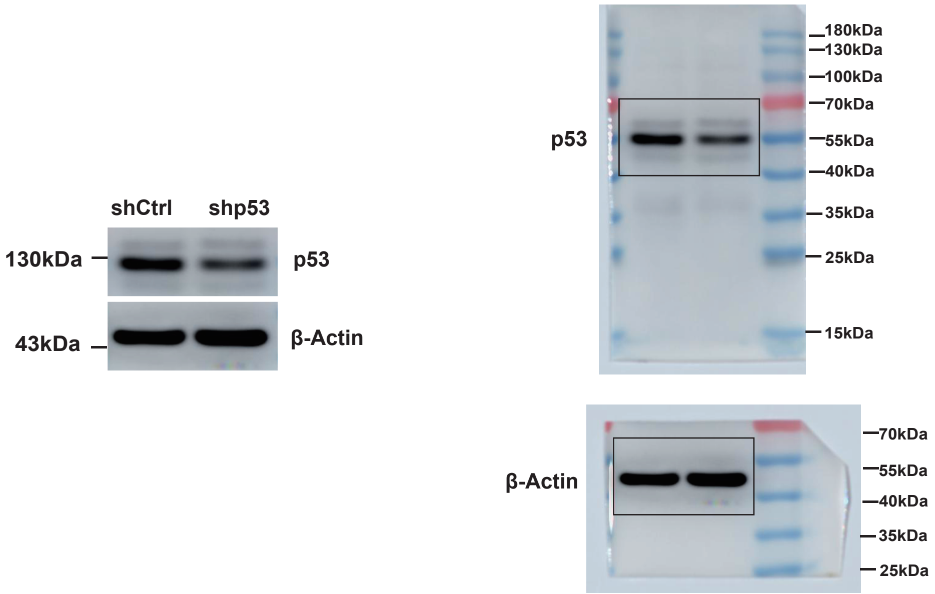

Figure 3C

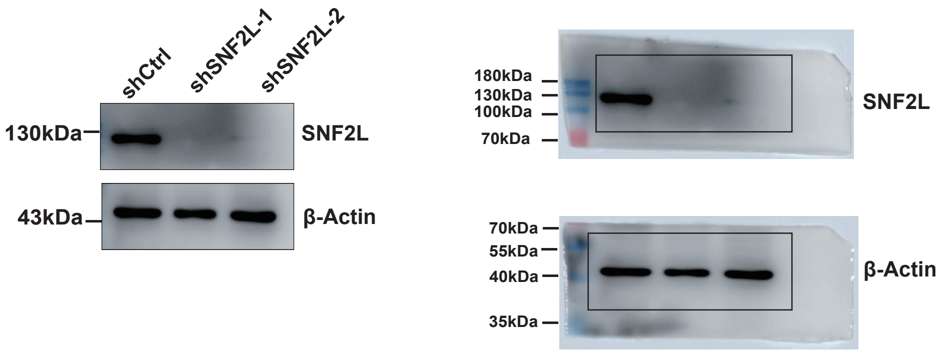

Figure 3F

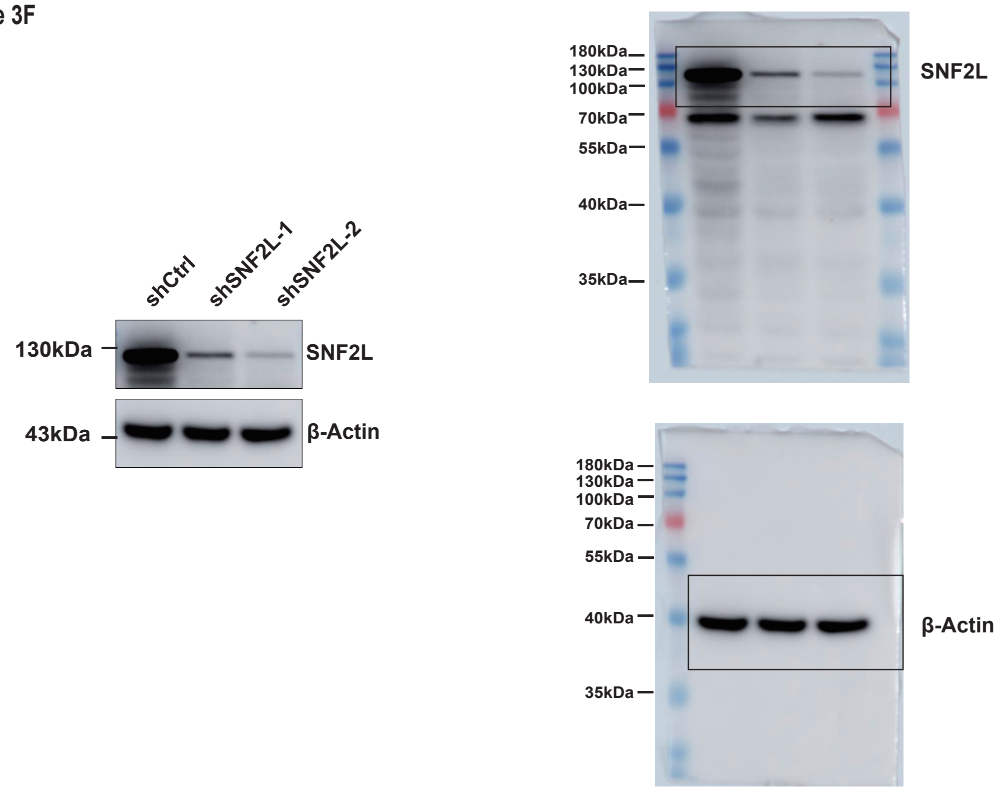

Figure 4D

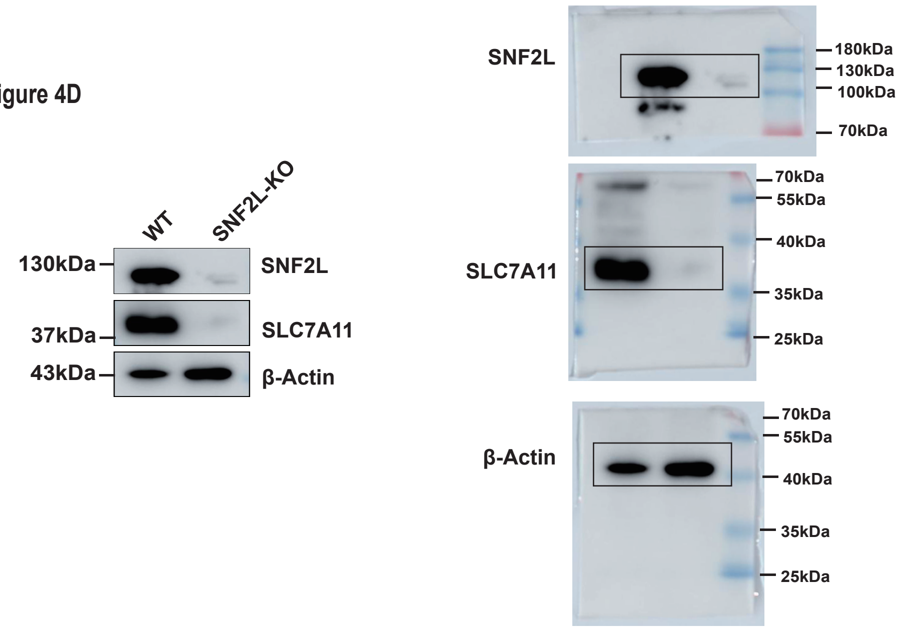

Figure 4E

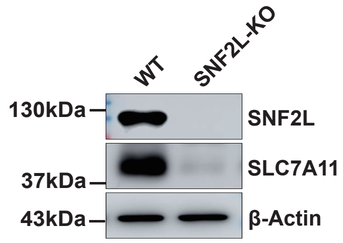
WT
SNF2L-KO
130kDa
37kDa
43kDa
SNF2L
SLC7A11
β-Actin

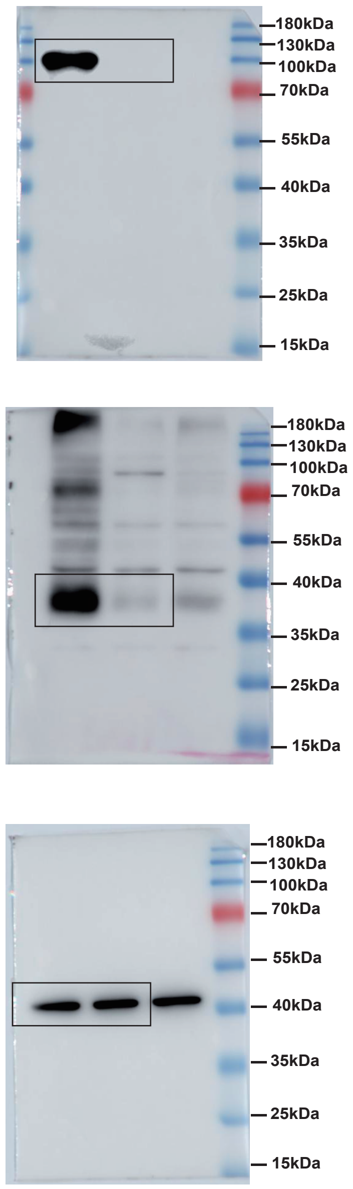
180kDa
130kDa
100kDa
70kDa
55kDa
40kDa
35kDa
25kDa
15kDa
180kDa
130kDa
100kDa
70kDa
55kDa
40kDa
35kDa
25kDa
15kDa
180kDa
130kDa
100kDa
70kDa
55kDa
40kDa
35kDa
25kDa
15kDa

Figure 4F

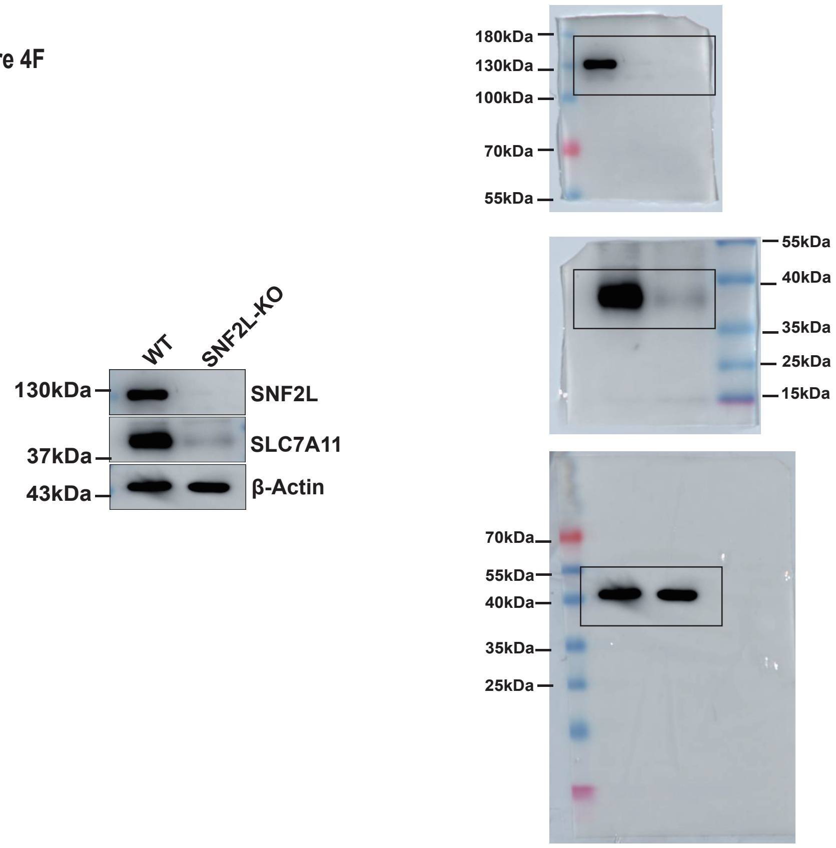

Figure 4G

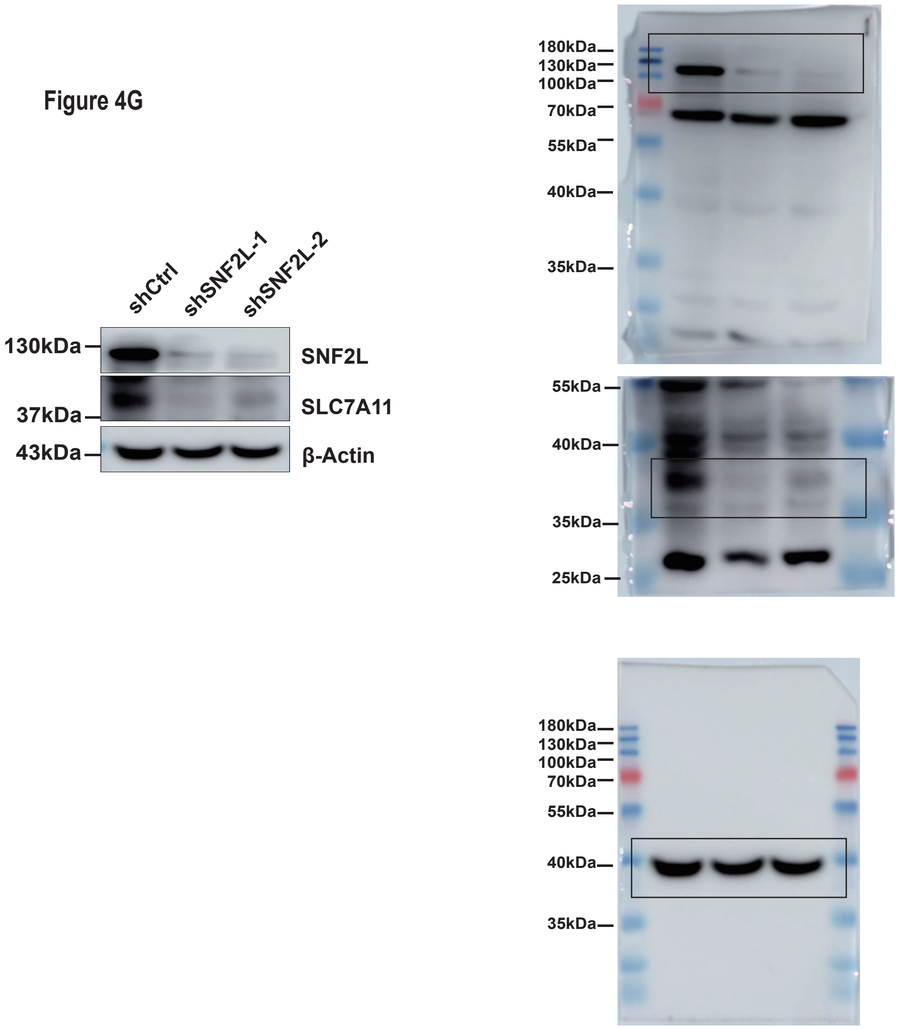

Figure 4H

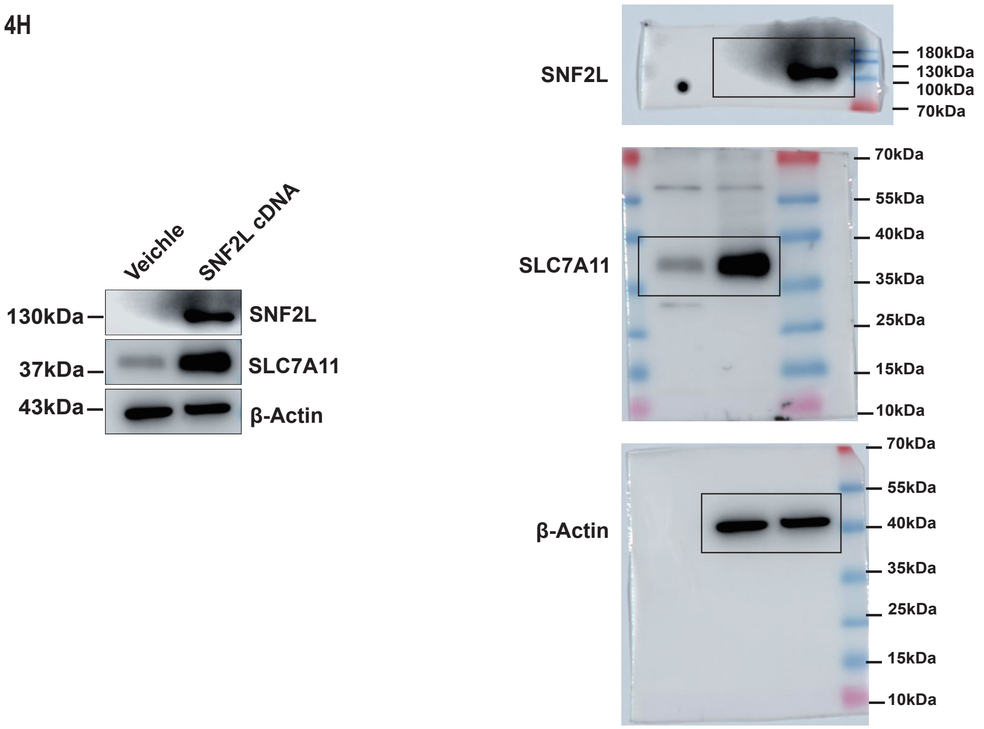

Figure 5A

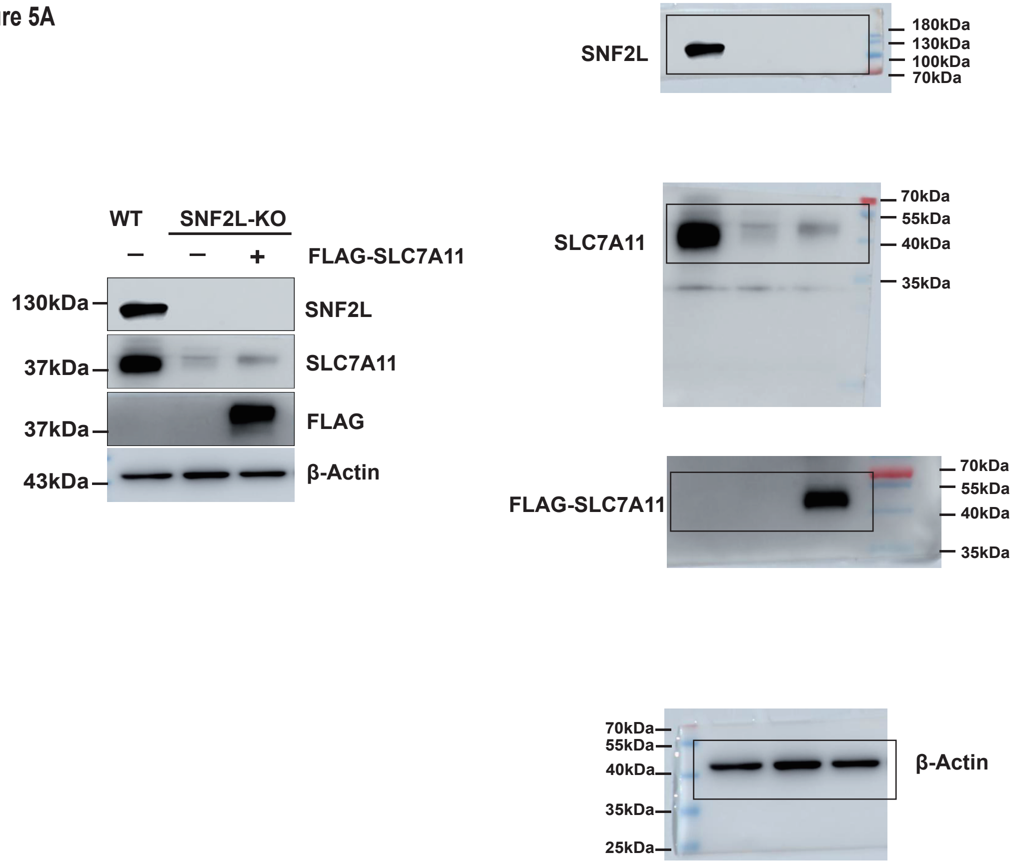

Figure 5C

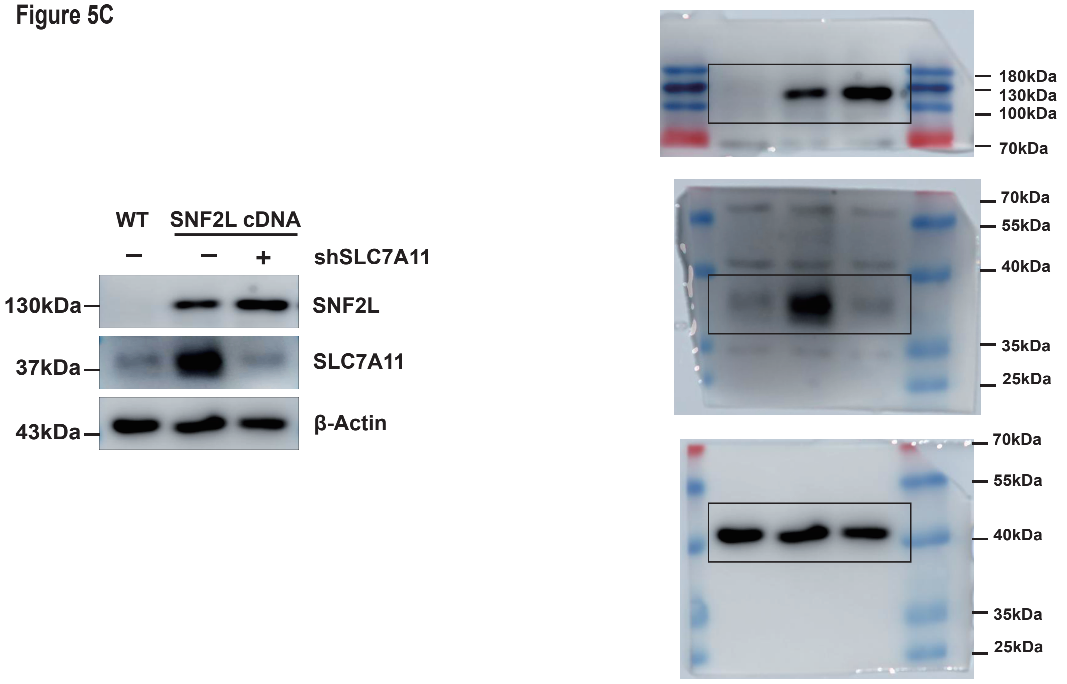

Figure 6D

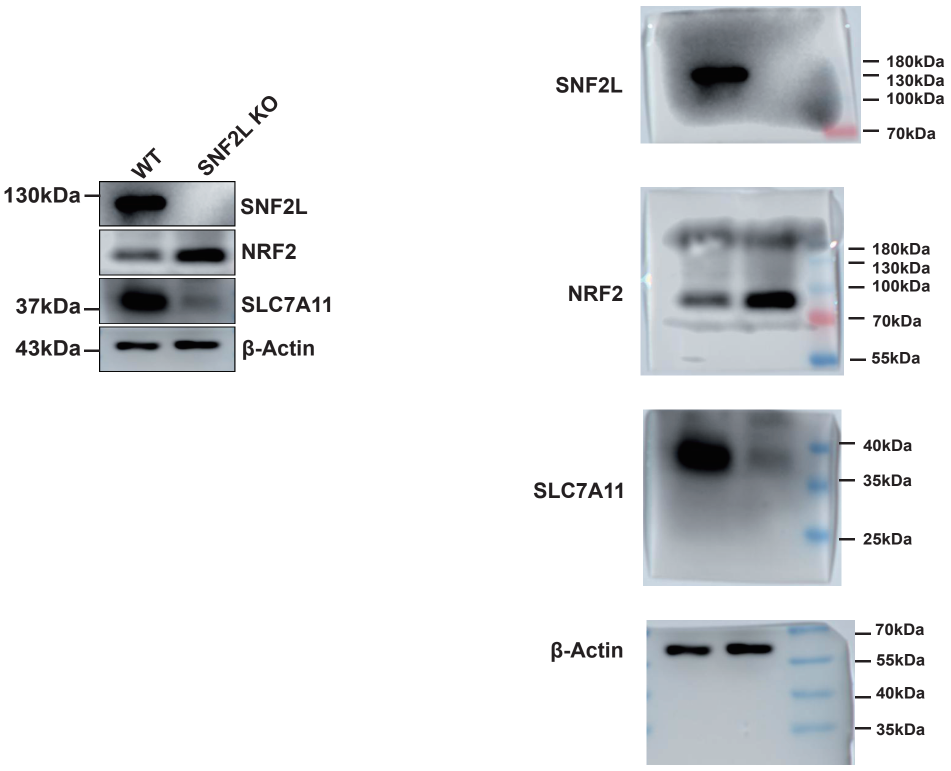

Figure 6E

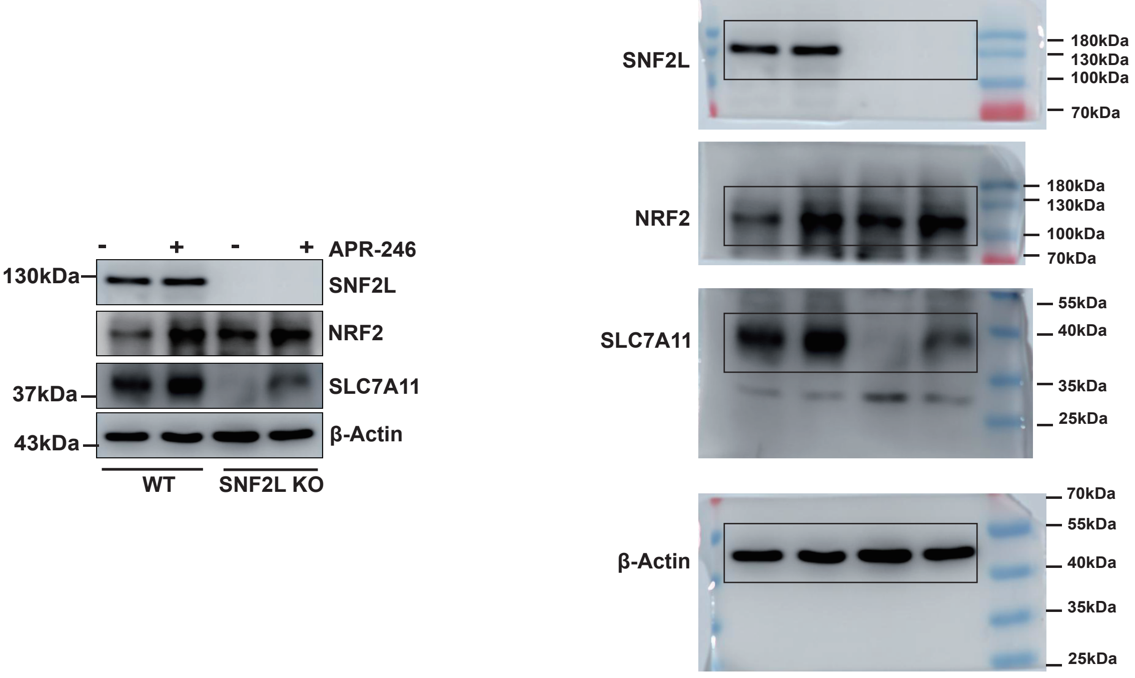

Figure S1B

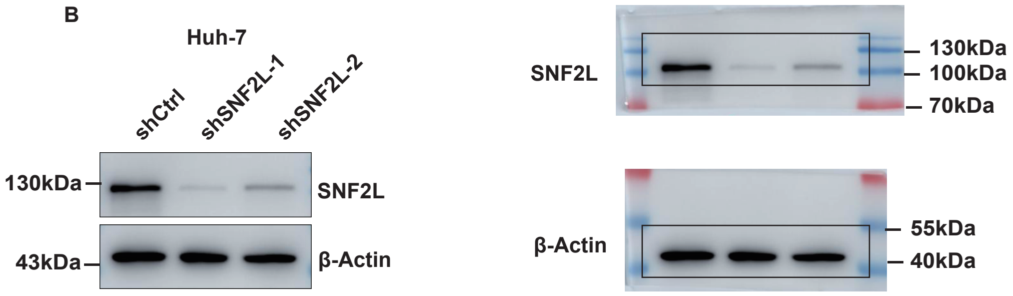

Figure S2A

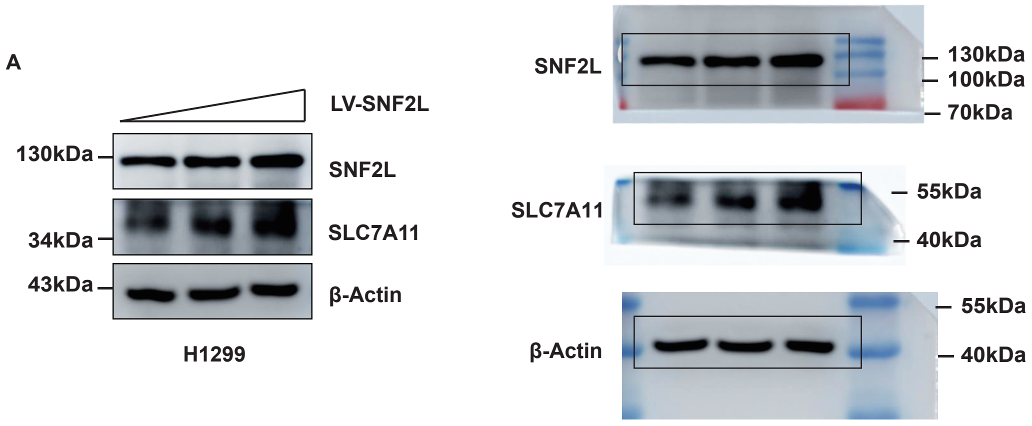

Figure S3A (the two lanes on the left)

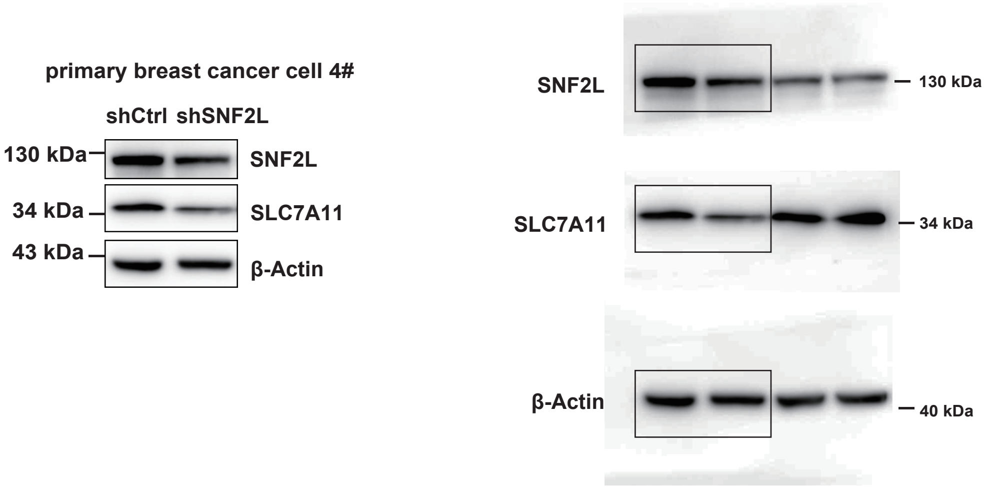

Figure S4E

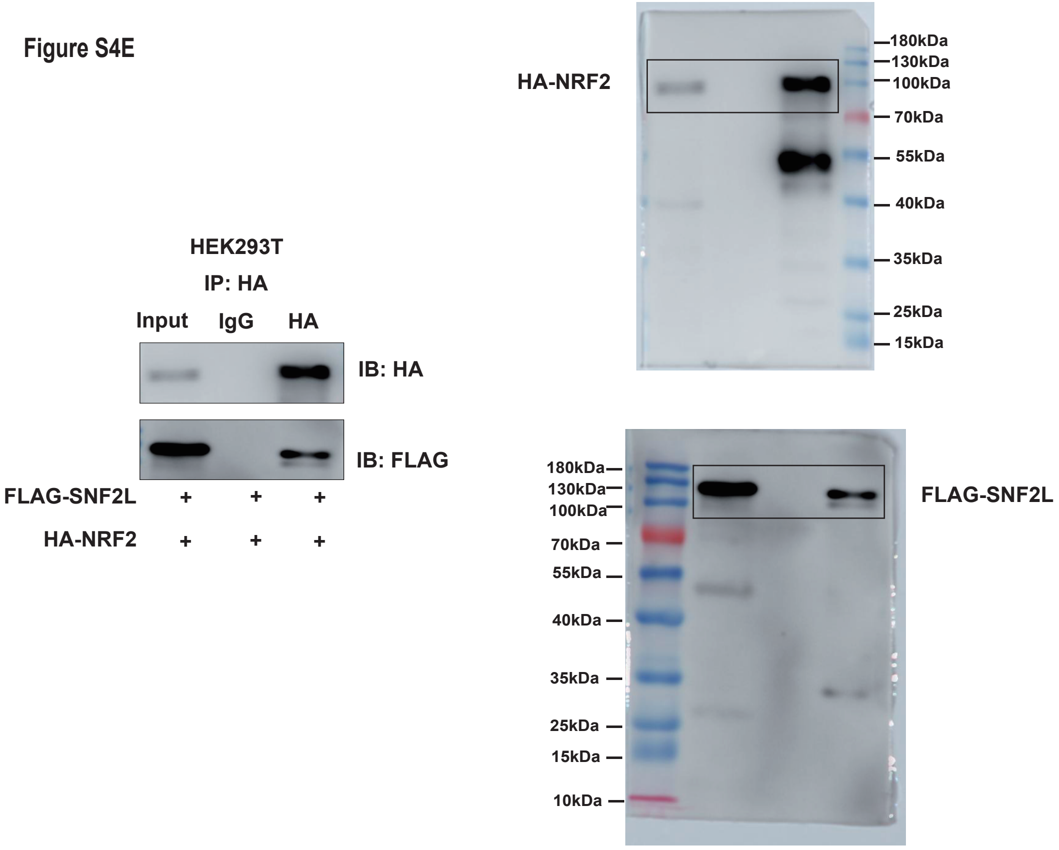

Figure S4F

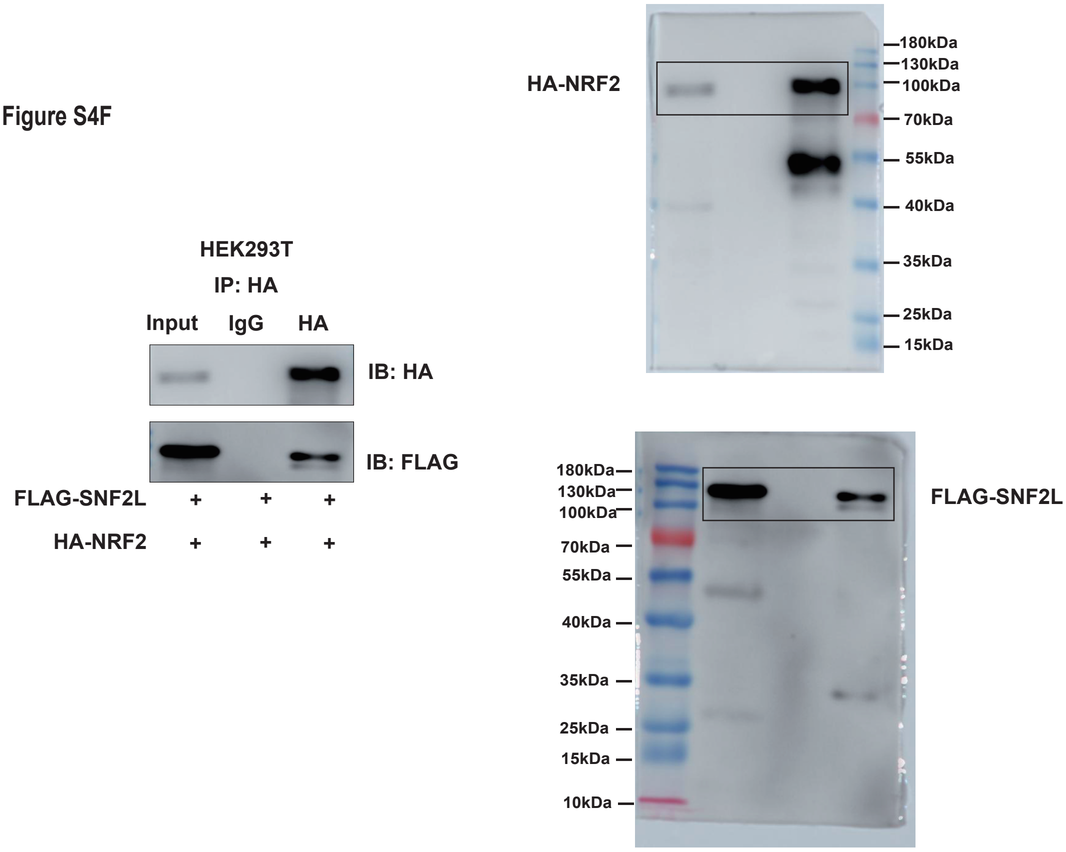

Figure S5B

B

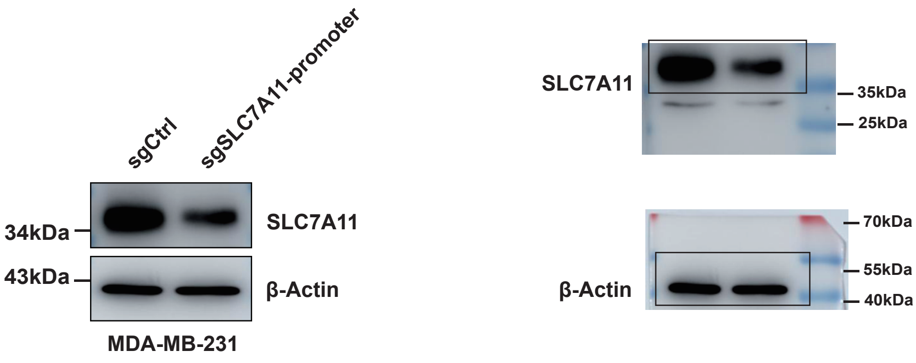

Supplement: Supplementary file 8 — uncropped original western blots [file 41419_2024_7221_MOESM8_ESM.pdf]
